# Evaluation of the Performance and Utility of Global Gridded Precipitation Products for Health Applications and Impact Assessments in South America

**DOI:** 10.1029/2024GH001260

**Published:** 2025-06-18

**Authors:** Sally Jahn, Katy A. M. Gaythorpe, Caroline M. Wainwright, Neil M. Ferguson

**Affiliations:** ^1^ MRC Centre for Global Infectious Disease Analysis School of Public Health Imperial College London London UK; ^2^ University of Leeds Leeds UK

## Abstract

Globally gridded precipitation products (GGPPs) are commonly used in impact assessments as substitutes for weather station data, each with unique strengths and limitations. Reanalysis products are among the most widely used for driving impact models, evaluating climate models, or bias‐correcting and downscaling model outputs to generate climate change projections. However, they are often outperformed in accuracy by other GGPPs, particularly in tropical regions, including areas of the Global South. Therefore, we assessed the utility and suitability of GGPPs for climate and health research by examining how differences and uncertainties in these products affect area‐level precipitation estimates, often used in health studies when epidemiological data are linked to administrative units. We compared reanalysis (ERA5/‐Land) with satellite‐based (CHIRPS, PERSIANN‐CDR) and interpolated gauge‐based products (CRUTS, GPCC), each a viable candidate to serve as reference climatology in climate change impact assessments. We focused on seasonal patterns, disease‐related bioclimatic variables, and climate change‐relevant indices, such as the number of wet or dry periods. Our findings revealed substantial variation in the accuracy of local precipitation estimates across GGPPs, with differences in maximum pixel precipitation values exceeding 75% between ERA5‐Land and CHIRPS. These differences in GGPPs translated into area‐level precipitation and, consequently, in vector carrying capacity estimates, demonstrating their impact on health assessments. Our analysis focused on Brazil and Colombia, two diverse countries differing for example, in orography, climate, and size. Each product was evaluated against national station data. Our results indicate that estimating tropical precipitation is particularly challenging for reanalysis, while CHIRPS demonstrated the best overall performance.

## Key Points

- Global gridded precipitation products vary in performance, affecting their utility for decision‐ and health‐relevant climate services
- Uncertainties in the spatially resolved products translate into aggregated area‐level precipitation estimates across heterogeneous regions
- Careful product selection can be crucial for accurate assessments of climate impacts on health, especially in data‐scarce areas

## 1 Introduction and Background

Reliable, continuous, and homogeneous records of ground‐based observations from weather stations often form the basis for impact assessments at the intersection of climate, weather, and health. However, assessments relying on station data and requiring high‐quality, long‐term coverage to evaluate climate change effects are hindered by the uneven distribution of stations, a challenge particularly pronounced in low‐ and middle‐income countries (LMICs) due to sparse networks, poor spatiotemporal coverage, limited documentation, non‐standardized quality control protocols, and restrictive data sharing policies and fees (Auffhammer et al., 2013; Bliefernicht et al., 2021; Schunke et al., 2021). (Quasi‐) global gridded (observational) climate data sets (GCDs), including AI‐generated data sets, remote sensing and satellite‐based data, as well as advanced model‐based data assimilation products such as reanalysis, might serve as substitutes. However, different GCDs are generally tailored toward specific applications, with each product having its own set of strengths and limitations. GCDs are prone to errors and biases, with the accuracy of their estimates often difficult to quantify and their performance exhibiting high temporal and spatial variability, particularly in regions with complex topography and pronounced climatic heterogeneity (Beck et al., 2017; Donat et al., 2014; Hassler & Lauer, 2021; Zandler et al., 2019).

Multiple GCDs from diverse sources are hence crucial for assessing observational uncertainty in climate impact assessments, as they serve as inputs for data‐driven impact models and can substantially influence their modeled outcomes throughout the observational period (Gebrechorkos et al., 2024; Tarek et al., 2020). Moreover, uncertainties in GCDs might also strongly affect future climate change projections (Baker et al., 2016; Tarek et al., 2021), as they serve as the reference climatology for the bias‐correction & downscaling (BC&D) of global and/or regional climate model (GCM/RCM) outputs. Respective BC&D methods are highly sensitive to the choice of GCD, with observational uncertainty being particularly important for precipitation and extreme precipitation indices (Iizumi et al., 2017; Kotlarski et al., 2017; Ludwig et al., 2019), as it can match or even exceed uncertainties from other major sources such as Representative Concentration Pathways and GCM spread. Nonetheless, it remains standard practice to evaluate, statistically downscale, and bias‐correct climate model outputs using a single GCD, even in the context of precipitation (Prein & Gobiet, 2017).

Reanalysis products like ERA5 (Hersbach et al., 2020) and ERA5‐Land (Muñoz‐Sabater et al., 2021) offer global spatial coverage and long temporal records, making them valuable GCDs for climate change and health research. However, various previous studies have conducted comparative analyses and performance evaluations of GCDs in tropical areas, including parts of the Global South, revealing substantial performance variations and generally lower accuracy of reanalysis data sets compared to other (quasi‐) global precipitation products (GGPPs) (Ahmed et al., 2024; Ayugi et al., 2024; Valencia et al., 2023). In general, reviews of GGPPs generally focused on assessing their overall performance and quality or quantifying their differences, often at a global level (Hassler & Lauer, 2021; Sun et al., 2018), with some specifically focusing on particular applications, such as hydrological analysis (Gebrechorkos et al., 2024; Voisin et al., 2008). Lavers et al. (2022) recommended using ERA5 precipitation data primarily for extratropical climate monitoring. ERA5 and ERA5‐Land are based on weather models designed for extratropical climates and perform poorly in regions with low station density, with their limited accuracy in tropical regions, including parts of the Global South, partly due to these differences between tropical and extratopical weather patterns and a lack of observational data. In contrast, CHIRPS (Funk et al., 2014, 2015), which integrates satellite‐based and weather station precipitation data, has demonstrated superior accuracy across diverse regions, elevation zones, and temporal scales (Ahmed et al., 2024; Dinku et al., 2018; Gebrechorkos et al., 2018; Valencia et al., 2023), though it still has representation limitations compared to station observations (Cavalcante et al., 2020; López‐Bermeo et al., 2022). Despite their known limitations, ERA5 and ERA5‐Land remain widely used and accepted in impact assessments (Hajat et al., 2022; He et al., 2024; Ortega‐Lenis et al., 2024) and frequently serve to evaluate climate models or correct model output biases in order to generate downscaled projections for climate impacts research worldwide (Gergel et al., 2024; Noel et al., 2021; Xu et al., 2021).

While research focusing on specific countries or regions often evaluated GGPPs against ground‐based station data from national or regional hydrometeorological services (Ahmed et al., 2024; Amjad et al., 2020; Gebrechorkos et al., 2018; Navidi Nassaj et al., 2022; Valencia et al., 2023; Wu et al., 2023), comparative assessments of GCDs against weather station data in the context of environmental epidemiology and health impact assessments remains limited (Colston et al., 2018; Mistry et al., 2022). Moreover, only few studies have extended beyond pixel‐scale analyses to consider administrative geographical units or evaluated the performance of spatially aggregated area‐level estimates, which are commonly used in health‐related impact assessments, and the limited research available has primarily focused on high‐income countries (de Schrijver et al., 2021; Spangler et al., 2019). Additionally, most studies have focused on mean responses, with limited attention to observational uncertainties in deriving indices for assessing extremes, such as the climate change indices recommended by the Expert Team on Climate Change Detection and Indices (ETCCDI, 2020; Zhang et al., 2011) in the context of climate change and health (Donat et al., 2014; Zhao et al., 2020). A similar gap exists for bioclimatic variables (BCVs), which are physiologically significant for species distribution and widely applied in vector‐borne disease research (Lim et al., 2023; Whittaker et al., 2022), yet few studies have examined observational uncertainty in BCV derivation (Merkenschlager et al., 2023; Morales‐Barbero et al., 2018).

Despite the growing literature on GGPPs comparison and evaluation, the question of identifying the most suitable and best‐performing GGPP for health applications and impact assessments as well as for driving impact models for climate change studies remains unanswered, particularly for the Global South. To our knowledge, no study has critically and comprehensively assessed the application of GGPPs from different sources and with varying spatial resolutions in the Global South, particularly with a focus on spatially aggregated area‐level precipitation estimates across administrative units. To address this gap, we focus on reanalysis, specifically ERA5 and ERA5‐Land, which are among the most widely applied and accepted products in climate change studies and impact assessments. We use CHIRPS as the main reference due to its demonstrated good performance in tropical regions, including parts of the Global South, alongside four additional GGPPs, each spanning a sufficiently long time period to define climate and potentially serve as reference data sets for bias‐correcting climate model outputs in climate change impact research. Since observational uncertainty is a particular concern in regions with sparse weather station coverage, and research on the Global South remains limited, this study focuses on South America. Brazil and Colombia are chosen for in‐depth analysis due to their distinct differences in size, population distribution, orography, and climate.

The main objective of our work extends beyond simply assessing data quality to evaluating GGPPs at their native resolution for direct application in impact and health research, where these products are typically utilized at their original spatial scale. To further examine the effect of spatial resolution on observational uncertainty, we include comparisons on a common 0.5° grid where applicable. Secondary objectives include evaluating GGPP performance in capturing seasonal patterns, ETCCDI, and BCVs, as shifts in seasonality and extremes are often key drivers of the most severe consequences of climate and climate change, for example, in the context of tropical diseases such as malaria (WHO, 2023). We conduct a comparative analysis between GGPPs and station data from Brazil and Colombia to confirm the lower performance of reanalysis and the superior performance of CHIRPS in our study domain. Additionally, we examine how GGPP differences translate into spatially aggregated area‐level precipitation estimates and, subsequently, test the sensitivity of estimates of rainfall‐dependent environmental vector carrying capacity used as an essential parameter in ecological models of mosquito populations in malaria research (White et al., 2011). Furthermore, we provide recommendations on the utilization of GGPPs based on spatial aggregation, regional context, and physiographic characteristics. In particular, we emphasize that careful product selection is essential for accurate impact assessments. GGPPs should not be chosen solely based on familiarity with the data provider, general acceptance, or spatial resolution, but rather on their suitability and impact within the specific research context.

## 2 Study Setting and Data

Here we introduce our study domain in South America. We present the station data, GGPPs and administrative boundaries for Brazil and Colombia that will be utilized in our study.

### 2.1 Study Domain and Station Data

#### 2.1.1 Precipitation in South America

In addition to typical precipitation patterns common to the Subtropics and Tropics, such as the increased rainfall associated with the Intertropical Convergence Zone (ITCZ), South America's precipitation is notably diverse. The continent includes some of the wettest regions on Earth, such as the Amazon or the western Andean slopes in Colombia, which experience exceptionally high rainfall, as well as some of the driest areas, like the Atacama Desert. Given the focus of our work on climate impact assessments in the health sector and the evaluation of reanalysis, we concentrated our analysis on South American regions north of 40° South, as these model‐based data sets are known to face challenges in accurately estimating tropical precipitation (Lavers et al., 2022). This domain is also particularly relevant as it encompasses regions with a high prevalence of tropical diseases, and, consequently, a substantial current and future population at risk for illnesses such as dengue, malaria, and yellow fever (Gaythorpe et al., 2021; Messina et al., 2019; Sinka et al., 2012).

#### 2.1.2 Validation Areas and Weather Stations

**Figure 1:**
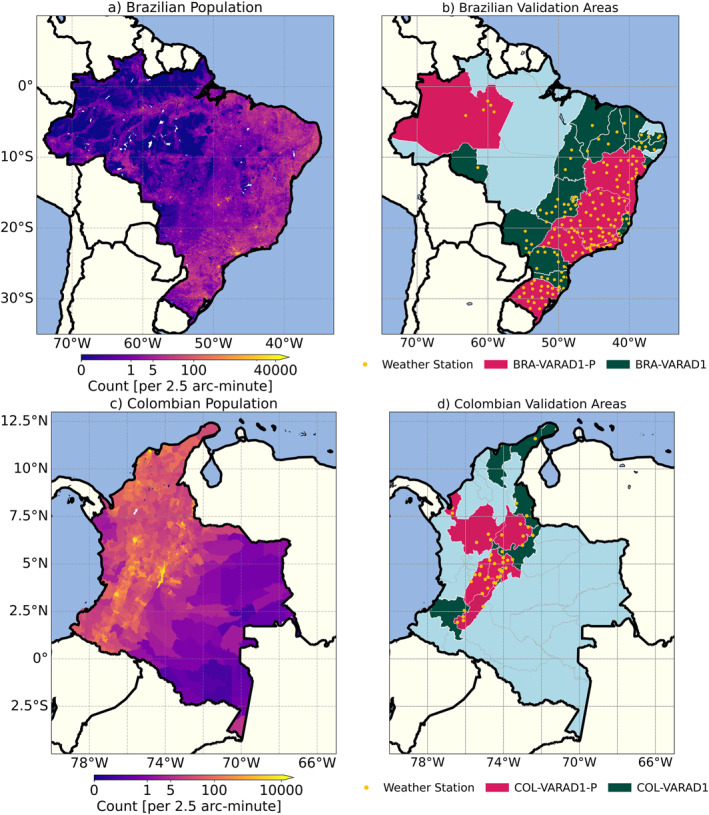
Maps illustrating population distribution and validation areas in Brazil and Colombia. Panels (a, c) display the Brazilian and Colombian population (inhabitants per 2.5 arc‐minute for each corresponding grid cell) in 2010, respectively. Panels (b, d) show the validation areas for each country, with primary validation areas highlighted in pink and secondary validation areas in green. Yellow dots indicate the locations of the selected weather stations. The boundaries of Brazil and Colombia are outlined using the Database of Global Administrative Areas, GADM (version 4.1). Note the different scales used to depict population in each country.

We selected, collated and processed station data to generate a homogenized database with consistent and reliable monitoring data. Precipitation data were provided by the Brazilian National Institute of Meteorology (INMET, Instituto Nacional de Meteorologia (2024)) and the Colombian Institute of Hydrology, Meteorology, and Environmental Studies Instituto de Hidrología & Meteorología & Estudios Ambientales (2024), respectively. To achieve consistent and sufficient station coverage across the study domain, we selected the years 2011–2020 as our evaluation period. Only stations that provided data for each year within this period were included. For Brazil, we obtained daily resolution data directly from INMET. For Colombia, IDEAM data were originally reported at hourly and sub‐hourly time steps. The evaluation of GGPPs and aggregated area‐level estimates was conducted for selected administrative units at subdivision level 1 (AD1) in Brazil and Colombia, referred to as validation areas below (see Figure 1). The selection of validation areas was based on availability, quality, and density of weather stations.

### 2.2 Global Gridded Precipitation Products

Table 1 summarizes the main characteristics of the selected GGPPs for this study. These GGPPs were selected since they are regularly and continuously updated, well‐established, and widely used in climate and environmental sciences. We defined a 30‐year base period (1991–2020) for GGPPs‐only analyses. We aimed to include GGPPs that are as independent as possible and derived from various sources, incorporating different and often mixed methods. We included GGPPs of the following types: (a) Interpolated data sets generated through geostatistical and spatial interpolation of ground‐based observations (b) Reanalysis created using data assimilation of historical periods, merging observations with model outputs (c) Satellite‐based data sets that estimated values from remotely sensed information combined with station data through various algorithms. All GGPPs were downloaded with a sub‐daily or daily time resolution, except CRUTS, for which only monthly estimates were available. Daily estimates were derived from the sub‐daily data sets. The different products are also briefly outlined and presented (Text S1 in Supporting Information S1).

**Table 1:** Summary of the Analyzed Globally Gridded Precipitation Products

Data set	Provider	Version	Type	Native resolution and coverage	Time range (starting from)	Main reference
CHIRPS*	Climate Hazards Center (CHC) at UC Santa Barbara	2.0	B	0.05° × 0.05° quasi‐global (approximately 50°S–50°N)	1981	Funk et al. (2014)
CRUTS*	University of East Anglia Climatic Research Unit	v.4.07	A	0.5° × 0.5° global	1901	Harris et al. (2020)
ERA5	European Center for Medium‐Range Weather Forecasts	Accessed 2023	C	0.25° × 0.25° global	1940	Hersbach et al. (2020)
ERA5‐Land*	European Center for Medium‐Range Weather Forecasts	Accessed 2023	C	0.1° × 0.1° global	1950	Muñoz‐Sabater et al. (2021)
GPCC*	Global Precipitation Climatology Center at Deutscher Wetterdienst (DWD)	v2022	A	1° × 1° global	1982	Ziese et al. (2022)
PERSIANN‐CDR	NOAA's National Climatic Data Center (NCDC) and the Center for Hydrometeorology and Remote Sensing (CHRS) at the University of California, Irvine	v01r01	B	0.25° × 0.25° quasi‐global (approximately 60°S–60°N)	1983	Ashouri et al. (2015)

*Note.* The data sets are listed in alphabetical order. Land‐only products are marked with an asterisk. All data sets were downloaded at their native resolution and with a (sub‐) daily time resolution, except CRUTS, for which only monthly data were provided. We included GGPPs of the following types: (A) Interpolated data sets generated through geostatistical and spatial interpolation of ground‐based observations (B) Reanalysis created using data assimilation of historical periods, merging observations with model outputs (C) Satellite‐based data sets that estimated values from remotely sensed information combined with station data through various algorithms.

### 2.3 Administrative Boundaries and Population Data

The boundaries of first level (AD1) administrative areas of Brazil and Colombia were downloaded from the Global Administrative Area Database (GADM), version 4.1 (Hijmans et al., 2022). We used the Gridded Population of the World, Version 4 (GPWv4, Revision 11) for the year 2010 as our population data set (CIESIN & SEDAC, 2018). We downloaded population count data at a resolution of 2.5 arc‐minutes (approximately 5 km) to best match the resolution of the selected GGPPs.

## 3 Methods

Here, we present the preparation of station and GGPP data, as well as the selection of some AD1 as validation areas (VARAD1). We also introduce the evaluation methods and metrics applied in this study. While the primary focus was on evaluating GGPPs at their native resolution, we also assessed the impact of changing spatial resolution on the uncertainty of the selected products. To do so, the GGPPs were re‐gridded to a coarser resolution, specifically a common 0.5° grid.

In particular, unless stated otherwise, we aimed to incorporate an evaluation of GGPPs both at their native resolution and on a common 0.5° grid in all analyses, including the comparison of GGPPs with station data. GGPP‐only analyses could only be conducted on the common 0.5° grid when GGPPs were directly compared to one another.

### 3.1 Station and Area Subsets

We checked the quality of the provided station data from Brazil and Colombia. Data for both countries were transformed into a uniform format, including converting missing values, and checked for the availability and completeness of dates. To create valid daily timeseries for Colombia, we aggregated the (sub‐)hourly information and created daily sums if more than 70% of the values were available for a given day. To minimize the influence of missing values, we only selected stations with coverage of more than 70% of daily values per month within the evaluation period. We excluded values that fell outside the physical limits for daily precipitation amounts (exceeding 1,000 mm per day). Overall, we retained 199 stations in Brazil and 49 stations in Colombia.

Figure 1 shows the population distribution and the locations of the selected weather stations for Brazil (a, b) and Colombia (c, d). The figure highlights the sites of all stations used in the analysis along with their assigned validation areas in Brazil and Colombia, referred to as BRA‐VARAD1 and COL‐VARAD1. An AD1 was designated as a validation area if at least one selected weather station was located within it. Hence, we selected 20 BRA‐VARAD1 and 11 COL‐VARAD1 areas (also refer to Table S1 in Supporting Information S1). From those, areas with the highest number of weather stations were chosen as primary validation areas (VARAD1‐P). Since weather stations tend to be situated in or near populated areas, the primary validation areas have high population density for each country (Figure 1). For Brazil, the BRA‐VARAD1‐P set included Bahia (2), Minas Gerais (9), Rio Grande do Sul (14), Rio de Janeiro (15), and São Paulo (19). Additionally, the Amazon region (1) was included due to its remote location and relatively high population. For Colombia, the COL‐VARAD1‐P set included Antioquia (1), Cundinamarca (5), Huila (6), Norte de Santander (9), Santander (10), and Tolima (11).

### 3.2 Spatial Aggregation and Precipitation Timeseries

We aggregated GGPPs spatially to create precipitation timeseries for all validation areas in Brazil and Colombia. This was done by at first extracting the daily values for each grid cell within or intersecting the boundaries of the respective AD1. We then averaged the precipitation data from all these extracted grid cells across the VARAD1. As we calculated these area‐level averages using data from a regular latitude‐longitude grid, we applied weights based on the cosine of the latitude to account for meridian convergence at higher latitudes. In total, we generated six daily precipitation timeseries for each BRA‐/COL‐VARAD1 sets, corresponding to each of the selected GGPPs at their native resolution. Equally, we also generated area‐level estimates for each validation area in Brazil and Colombia by using GGPPs on a common 0.5° grid, with GGPPs being resized to a coarser resolution using bilinear interpolation. We aggregated monthly information from the daily precipitation timeseries. Here, we focused in general on monthly resolution, but also considered daily timescales for specific analyses, reflecting the importance of shorter time scales for certain health‐related outcomes. We also provide an example from the health sector (Section 3.3.3 and 4.2.3) based on daily temporal resolution, to highlight the relevance of our findings and to aid in their illustration and visualization.

### 3.3 Evaluation Methods and Metrics

#### 3.3.1 Statistical Metrics

The performance of the GGPPs considered was primarily evaluated using standard statistical methods. These included the Pearson correlation coefficient (PCC), relative bias (Rbias), which indicates average differences and systematic bias between time series from different sources, and root mean square error (RMSE). PCC was reported only when statistically significant at the 95% confidence level. Additional details on the metrics can be found (Text S2 in Supporting Information S1).

#### 3.3.2 Evaluation of Precipitation Timeseries

The most common approach for comparing ground‐based observations with gridded estimates is a point‐to‐pixel comparison. Accordingly, we compared individual station timeseries with corresponding pixel values using the three metrics. To evaluate the overall performance and accuracy of each product for each validation area, we then calculated the average of the individual point‐to‐pixel values for each VARAD1. Thus, we derive a summary statistic that represents how well each product performs across a whole area by averaging the results from multiple individual comparisons. For a few stations, some GGPPs at their native resolution, specifically ERA5‐Land, did not provide pixel‐based information, especially for locations along the coast. Hence, we excluded some stations to account for discrepancies between station locations and grid points (totaling nine in Brazil and one in Colombia). However, it is important to note that pixel values represent estimates over large areas, and the grid resolution of the GGPP directly influences the representativity of these estimates. GGPPs with coarser resolutions are generally expected to be less precise in capturing localized conditions, that is, in the vicinity of weather stations. While the primary objective of our work was to evaluate the impact of both data quality and resolution on the accuracy of the GGPPs–acknowledging that GGPPs are often used at their native resolution in impact assessments–we also conducted the same analysis for GGPPs re‐gridded to a common 0.5° grid. This allows for a more thorough evaluation of data quality independent of the influence of spatial resolution. We hence conducted respective point‐to‐pixel analysis by using GGPPs at their native resolution as well as for comparison on a common 0.5° grid.

The spatial aggregation of GGPPs might involve averaging grid cells that represent very different conditions across administrative areas, depending on factors such as size, specific orography, and climate. Hence, averaging them together may not provide any meaningful interpretation and may reduce the useful skill and representativity of the applied information. Given precipitation can vary substantially over small spatial scales, we also compared GGPP aggregated up to AD1 level with each pixel‐based timeseries. Here, results are only shown for GGPP at their native resolution.

#### 3.3.3 Seasonal Representation of Area‐Level Estimates and Carrying Capacity

We further evaluated the annual cycles of precipitation estimates derived from the aggregated area‐level precipitation timeseries for each GGPPs over the base period. This analysis covered Brazil and Colombia as a whole, as well as VARAD1‐P sets within each country. These comparisons allowed for a more accurate assessment of data set biases resulting from underlying climatological differences. Additionally, they helped to identify whether these biases exhibited a specific seasonal pattern or remained relatively constant throughout the year, and whether the annual cycle's shape was consistent across the various GGPPs.

We subsequently assessed the sensitivity of estimates from an aggregated precipitation function, commonly used in models to represent the seasonality of vector carrying capacity, to variations in GGPP input, based on spatially aggregated, area‐level precipitation estimates. For details and an example of an ecological model of *Anopheles gambiae sensu lato* populations incorporating rainfall‐dependent carrying capacity equation, refer to White et al. (2011). In their study, three models for calculating environmental carrying capacity were tested, and the model in which carrying capacity was proportional to exponentially weighted past rainfall was found to be the best fit. Accordingly, we employed this model to calculate environmental carrying capacity, *K*(*t*), assuming it to be proportional to the aggregated rainfall of preceding days (White et al., 2011; Ye et al., 2009). The formula is presented below, where rain(*t*) is the daily rainfall, τ denotes the number of preceding days considered, and λ is a fitted scaling factor:

$$K\left(t\right)=\lambda \frac{1}{\tau \left(1-e^{-t/\tau }\right)}\int_{0}^{t}e^{-\left(t-t^{\prime }\right)/\tau }\text{rain}\left(t^{\prime }\right)dt^{\prime }$$

In modeling vector‐borne diseases, particularly malaria, the parameter λ serves as a scaling factor that adjusts the carrying capacity function to align with observed ecological conditions. Since λ is specific to each study area, it must be calibrated to accurately reflect local environmental factors. While λ is not fixed and would typically require calibration for each study site in South America, we adopted a simplified value of 1 across our analysis. This approach was chosen because the precise value of λ has no impact on our assessment of the carrying capacity's sensitivity to GGPP input. Similarly, we set *τ* = 4, a commonly used value in malaria research (White et al., 2011). CRUTS was excluded from the analysis, as K(t) is derived using daily values.

We conducted both analysis, the annual cycle representation and the sensitivity analysis of environmental vector carrying capacity, using GGPPs at their native resolution and for comparison on a common 0.5° grid.

### 3.4 Climate Change Indices and Bioclimatic Variables

We examined how uncertainties in GGPPs influence the derivation of ETCCDI and a selection of BCVs focusing on precipitation. Daily data were used to generate ETCCDI (excluding information from CRUTS), while the input for calculating BCVs was based on climatological monthly means. We calculated the ETCCDI as implemented in the CDO software (Schulzweida & Quast, 2015) to derive climatic indicators of precipitation extremes. We focused on a subset of four indices: simple daily intensity index (SDII), very heavy precipitation days index (R20 mm), consecutive wet (CWD) and consecutive dry days (CDD). There exist multiple computational schemes to generate BCVs. Here, we used the function BIOVARS from the dismo R package to generate BCV estimates. We focused on a subset of six BCVs: total annual precipitation (BCV12), precipitation of the wettest and driest month or quarter (BCV13‐14 and BCV16‐17), and precipitation seasonality (BCV15). For detailed definitions and calculations of the selected ETCCDI and BCVs, refer to Table S2 in Supporting Information S1. In this context, while ETCCDI and BCVs were extracted based on their native resolutions and on a common 0.5° grid, we compared GGPPs to each other using a common grid.

## 4 Results

### 4.1 Differences and Uncertainty in GGPPs

We first examine variation between the GGPPs in terms of the geographical distribution of precipitation totals and derived ETCCDI and BCV across the South American study domain.

#### 4.1.1 Geographical Distributions and Observational Data Uncertainty

**Figure 2:**
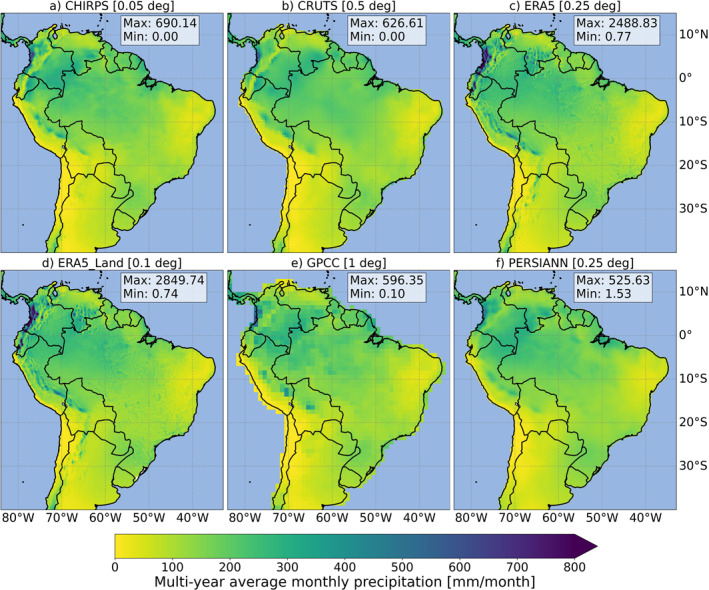
Multi‐year average monthly precipitation [mm/month] across South America for each globally gridded precipitation product (GGPP) (a–f), averaged over the base period (1991–2020). The top row presents CHIRPS (a), CRUTS (b), and ERA5 (c), while the bottom row features ERA5‐Land (d), GPCC (e), and PERSIANN (f). The maps display precipitation data at their native spatial resolution. Within each map, boxes indicate the pixel‐based minimum (min) and maximum (max) precipitation values for each GGPP across the study domain.

Figure 2 presents the geographical distribution of the multi‐year monthly mean of total precipitation from each GGPPs, calculated over the base period. All gridded precipitation data sets consistently captured the major geographical patterns of precipitation, such as the high rainfall observed in parts of Colombia and the Amazon Basin, as well as the aridity of the Atacama Desert. However, notable differences in the estimated precipitation amounts emerged, particularly in western mountainous and coastal regions of South America. These differences were especially pronounced for ERA5 and ERA5‐Land, also highlighted by the pixel‐based maximum (max) and minimum (min) values (depicted in boxes). For reanalysis products, the multi‐year monthly precipitation reached a max of approximately 2,850 mm/month (d, ERA5‐Land) and 2,490 mm/month (c, ERA5), compared with values ranging from 525 mm/month (f, PERSIANN) to 690 mm/month (a, CHIRPS) for other GGPPs. Reanalysis products showed unrealistically high monthly precipitation amounts primarily in western regions of the study domain, particularly in parts of Colombia. Refer to Figure S1 in Supporting Information S1 for a more detailed representation of Colombia.

**Figure 3:**
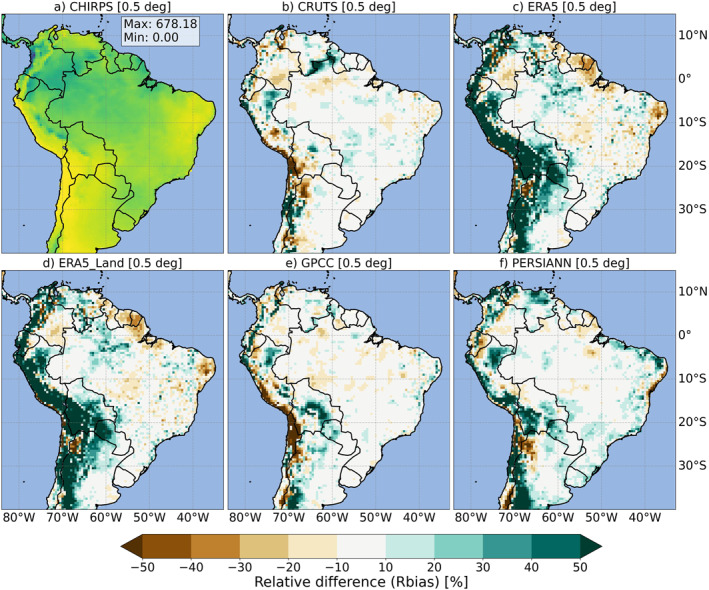
Multi‐year average monthly precipitation [mm/month] for CHIRPS (a, legend as in Figure 2) and the relative difference (Rbias in %) from CHIRPS for all other globally gridded precipitation products (b–f), averaged over the base period (1991–2020), across the South American study domain. The top row shows CHIRPS (a), CRUTS (b), and ERA5 (c), while the bottom row displays ERA5‐Land (d), GPCC (e), and PERSIANN (f). All maps are presented at a common 0.5° spatial resolution.

Figure 3 further illustrates the relative differences (Rbias in %) in precipitation across the study domain, comparing each GGPP against CHIRPS as the reference (comparison based on all GGPPs on a common 0.5° grid). Notably, several GGPPs, especially reanalysis products (c, d), exhibited a pronounced wet bias over large portions of the western study domain, particularly in Andes‐dominated regions and coastal areas, and generally in zones typically characterized by extreme humidity or aridity (however, especially the CRUTS and GPCC in b and e showed a tendency toward a dry bias in some parts of this region). This means that the model‐based data sets overestimated precipitation over these areas compared to CHIRPS (a) during the base period. Dry biases in reanalysis were particularly evident in the northeastern coastal regions of South America, including areas surrounding Suriname, French Guiana, and some coastal parts of Northeast Brazil. Refer to Figure S2 in Supporting Information S1 for a more detailed representation of Colombia.

#### 4.1.2 Differences in ETCCDI and BCVs

**Figure 4:**
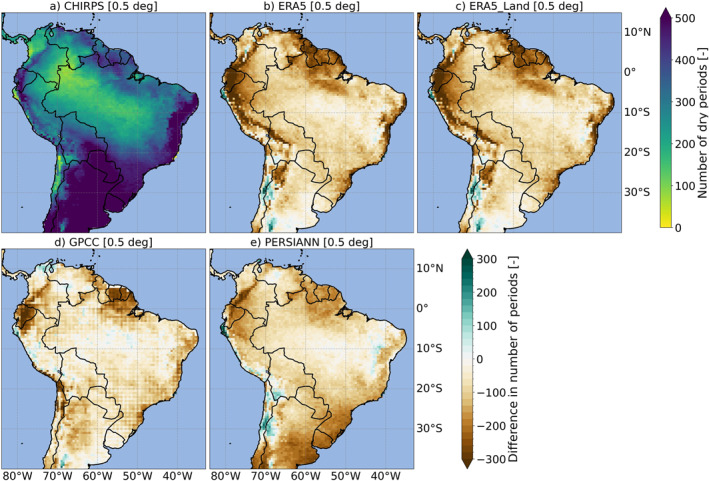
Consecutive dry days (CDD)–the number of dry periods lasting more than 5 days [−] for CHIRPS (a). For all other globally gridded precipitation products (b–e), the difference [−] compared to CHIRPS is shown. All values represent averages over the base period (1991–2020). Note that CRUTS was excluded from the analysis, as CDD was calculated using daily values. The top row includes CHIRPS (a), ERA5 (b), and ERA5‐Land (c), while the bottom row shows GPCC (d), and PERSIANN (e). The maps are presented on a common 0.5° grid.

**Figure 5:**
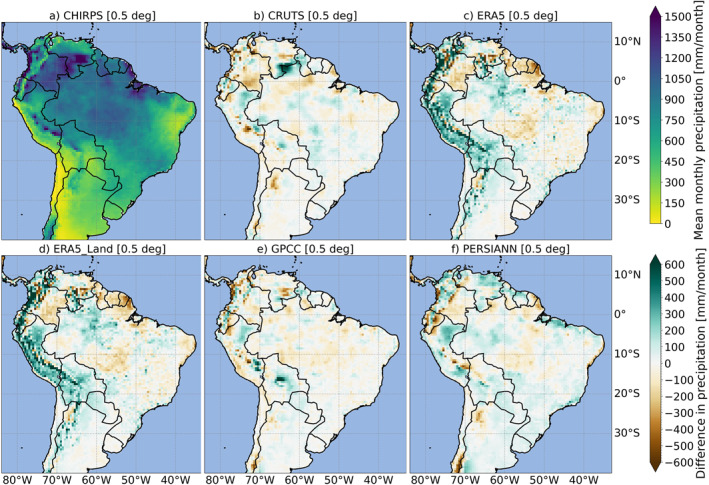
BCV16–the mean monthly total precipitation [mm] of CHIRPS (a) of the wettest quarter and, for all other globally gridded precipitation products (b–f), the difference [mm] compared to CHIRPS is shown. All values are based on climatological monthly averages calculated over the base period (1991–2020). The top row shows CHIRPS (a), CRUTS (b), and ERA5 (c), while the bottom row displays ERA5‐Land (d), GPCC (e), and PERSIANN (f). The maps are presented on a common 0.5° grid.

Differences in GGPPs, such as those based on varying resolutions and interpolation schemes, can impact not only the total precipitation amounts but also the representation of spatial and temporal patterns. These variations hence might influence the calculation of extreme indices and variables like ETCCDI and BCVs. Figures 4 and 5 illustrate our findings for CDD and BCV16, while results for the other ETCCDI and BCVs are provided (see Figures S3–S11 in Supporting Information S1). For BCV13 and BCV16 (BCV14 and BCV17), we also evaluated the climatological months and quarters of the wettest and driest month and quarter (shown exemplarily for BCV16 in Figure S10 in Supporting Information S1).

In terms of the spatial representation of BCVs based on climatological monthly means averaged over the base period, similar patterns to those observed in Figure 3 became evident for BCV12 and, to a lesser extent, for BCV13, BCV14, BCV16, and BCV17. Differences were again more pronounced in coastal areas and regions with complex terrain (e.g., Andes‐dominated areas). Notably, these discrepancies were more prominent in reanalysis compared to CHIRPS. For example, Figure 5 illustrates that mean monthly precipitation differences (wettest quarter) from CHIRPS were especially pronounced in regions like these for ERA5 (c) and ERA5‐Land (d). The seasonality of precipitation is illustrated by BCV15. Unsurprisingly, due to its geographic location, Colombia exhibited a relatively weak seasonal precipitation signal, while certain areas of Brazil showed a pronounced seasonal variation in their climatological annual cycle, as depicted for CHIRPS (Figure S9a in Supporting Information S1). Notably, GGPPs generally underrepresented the seasonality signal of precipitation compared to CHIRPS, particularly in the mountainous and arid coastal western regions of the study area (however, some exceptions were observed, where specific GGPPs, especially CRUTS, overestimated seasonality in localized areas). In these regions, precipitation often displayed a stronger climatological seasonal cycle.

The analysis of the climatological months or quarters for the wettest and driest periods generally showed consistency across GGPPs when examining major large‐scale patterns in South America. However, some variation appeared on smaller scales. These differences might be influenced, though not determined, by variations in spatial resolution. For instance, as illustrated for BCV16 (wettest quarter) in Figure S10 in Supporting Information S1, PERSIANN and ERA5 (both at 0.25° resolution) revealed different quarterly precipitation maxima in some mountainous and southwestern regions of Colombia, with PERSIANN highlighting summer and ERA5 identifying winter maxima.

### 4.2 Comparison of Area‐Level Timeseries

We now describe how the six GGPPs translated into varying country‐ and unit‐specific aggregated area‐level precipitation estimates. Since the country‐specific timeseries were generally consistent across each GGPPs, whether averaged from products on a common 0.5° grid or at their native spatial resolution, the country‐level analyses are presented only based on the latter.

#### 4.2.1 Differences in Country‐Level Estimates

Given the size of South American countries, one might expect the different GGPPs to produce similar annual seasonal precipitation distributions. This was generally observed for Brazil, with precipitation distributions being comparable across all precipitation timeseries, with mean values ranging from about 145 mm (GPCC) to 152 mm (ERA5). The interquartile range (IQR) was between 132 and 135 mm, though slightly lower for CRUTS (about 126 mm) and GPCC (about 118 mm). However, being smaller in size, generally wetter, and characterized by complex terrain, Colombia exhibited noticeable differences in annual precipitation distributions across the examined GGPPs. The reanalysis‐based country‐specific series for Colombia showed a pronounced wet bias, with a noticeable shift toward higher precipitation amounts. This shift was evident in the higher mean and max values, which exceeded those of all other GGPPs by approximately 40 mm or more. The IQR varied from about 87 mm (CRUTS) to 97 mm (ERA5‐Land) and reached 104 mm (CHIRPS). See Table S3 in Supporting Information S1 for further details.

We analyzed how the choice of GGPPs affected ETCCDI and BCV estimates in Brazil and Colombia using the aggregated precipitation timeseries. Noticeable differences were observed, particularly in Colombia (see Table S4 in Supporting Information S1 for details). Reanalysis products exhibited discrepancies (relative to CHIRPS) exceeding 500 mm for BCV12. This equated to relative differences of approximately 18% (19%) between ERA5‐Land (ERA5) compared with CHIRPS. Similar relative differences, indicating an overestimation of precipitation in the reanalysis, were observed for BCV13 (around 16%), BCV14 (32%–35%), BCV16 (approximately 17%), and BCV17 (30%–32%). As a result, while the wet bias in reanalysis impacted both the wettest and driest months (or quarters), differences were more pronounced during periods of low precipitation and dryness. Deviations were also evident in the identification of CDD, as reanalysis failed to capture any dry spells. SDII showed higher daily precipitation amounts, while R20 mm indicated fewer days with heavy rainfall for reanalysis. CWD revealed a very strong underrepresentation of CWD in ERA5 and ERA5‐Land, compared to all other precipitation timeseries. Thus, while reanalysis presented a mixed picture when GGPPs were compared, with an increased number of wet periods across most of the country and fewer in some coastal areas, the spatial aggregation of reanalysis products at country level resulted in fewer identified prolonged rainfall episodes compared to other GGPPs.

**Figure 6:**
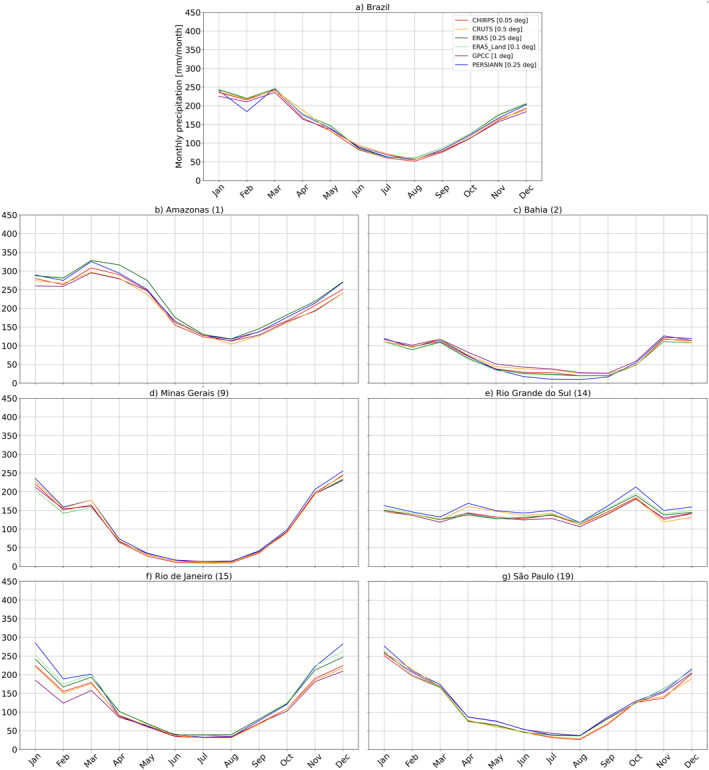
Climatological annual cycles of monthly precipitation (mm/month) for Brazil (a) and BRA‐VARAD1‐P regions (b–g), calculated over the base period (1991–2020). The uppermost figure (a) displays the precipitation amounts for Brazil as a whole. The subsequent figures are organized as follows: the top row includes (b) Amazonas (1) and (c) Bahia (2); the middle row presents (d) Minas Gerais (9) and (e) Rio Grande do Sul (14); and the bottom row features (f) Rio de Janeiro (15) and (g) São Paulo (19). These climatological annual cycles are based on area‐level precipitation timeseries derived from globally gridded precipitation products at their native spatial resolution.

**Figure 7:**
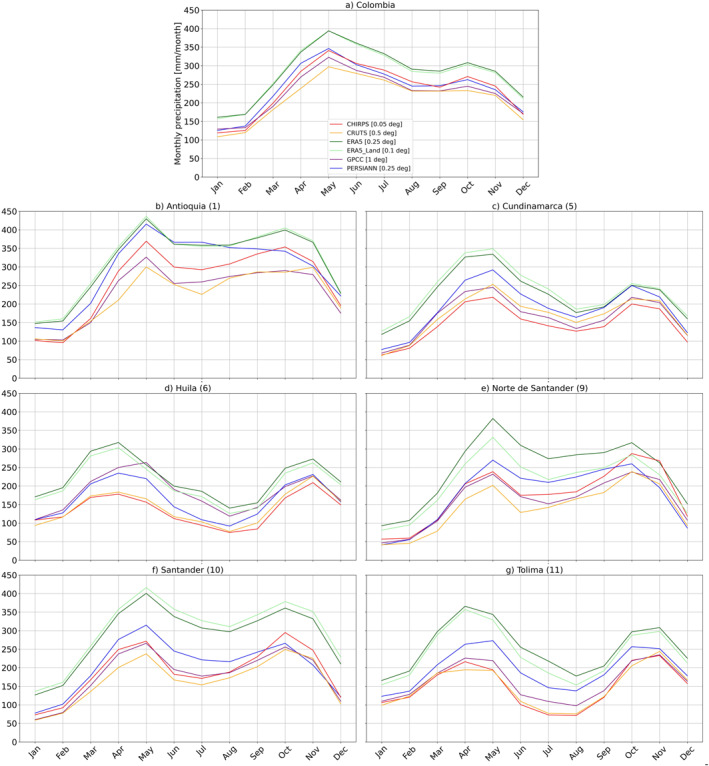
Climatological annual cycles of monthly precipitation (mm/month) for Colombia (a) and COL‐VARAD1‐P (b–g), calculated over the base period (1991–2020). The uppermost figure (a) displays the precipitation amounts for Colombia as a whole. The subsequent figures are organized as follows: the top row includes (b) Antioquia (1) and (c) Cundinamarca (2); the middle row presents (d) Huila (6) and (e) Norte de Santander (9); and the bottom row features (f) Santander (10) and (g) Tolima (11). These climatological annual cycles are based on area‐level precipitation timeseries derived from globally gridded precipitation products at their native spatial resolution.

At the country level, Figure 6 shows that Brazilian climatological annual cycles based on aggregated precipitation estimates showed no major differences and were mainly consistent throughout the year. For Colombia (Figure 7), the cycles revealed notable discrepancies across the GGPPs. However, they generally followed a similar pattern, and the differences remained relatively constant throughout the year. The most pronounced discrepancies were characterized by a clear offset and a shift toward wetter conditions in reanalysis products compared with other GGPPs. Refer to Figures S12 and S13 in Supporting Information S1 for climatological annual cycles from GGPPs on a common 0.5° grid.

#### 4.2.2 Differences Based on Administrative Units

For the BRA‐VARAD1‐P and COL‐VARAD1‐P sets of AD1 areas, we compared area‐level precipitation estimates from GGPPs at their native resolution (see Figures 6 and 7) with those interpolated to a common 0.5° grid (Figures S12 and S13 in Supporting Information S1). Figures S14–S17 in Supporting Information S1 display the corresponding monthly area‐specific precipitation distributions. It is important to interpret these results within the context of each country's specific characteristics, including the varying sizes of administrative units (see Table S1 in Supporting Information S1), with Colombian AD1s generally being of smaller size.

In Brazil, aggregated AD1 area‐level precipitation estimates were generally more consistent when derived from GGPPs re‐gridded to a common 0.5° grid, both regarding monthly distributions as well as climatological annual cycles, with notable (seasonal) deviations being evident in some BRA‐VARAD1‐P areas, such as Amazonas (1) and during winter in Rio de Janeiro (15). Figure S12 in Supporting Information S1 highlights that differences in climatological timeseries are mostly further reduced when based on GGPPs interpolated to a common 0.5° grid. This reduction may indicate two factors: first, it suggests that spatial resolution, rather than differences between the products themselves, plays a larger role in determining variations in aggregated area‐level estimates in Brazil. Second, as the products exhibit stronger variations and some enhanced biases at specific pixels, bilinear interpolation may smooth out and reduce extreme values and pronounced variations, potentially minimizing differences between data products.

In Colombia, larger differences were again observed between GGPPs. Reanalysis products consistently exhibited a wet bias across all COL‐VARAD1‐P for reanalysis, with ERA5 and ERA5‐Land showing a shift toward higher precipitation amounts compared to other GGPPs. Differences between data sets in climatological annual cycles either remained consistent throughout the year or varied by season, depending on the area. For example, in Cundinamarca (5) and Tolima (11), the biases in reanalysis‐based estimates were less pronounced in autumn. The impact of interpolation was more evident as well, leading overall to shifts in precipitation distributions, but especially influencing the discrepancies between precipitation timeseries in certain COL‐VARAD1‐P areas. In Tolima (11), for instance, the annual cycles showed seasonally varying offsets in reanalysis‐based estimates compared with CHIRPS, with stronger differences observed when area‐level precipitation estimates were based on GGPPs on a common 0.5° grid (refer to Figures 7 and S13 in Supporting Information S1).

#### 4.2.3 Translation Into Vector Carrying Capacity Estimates

**Figure 8:**
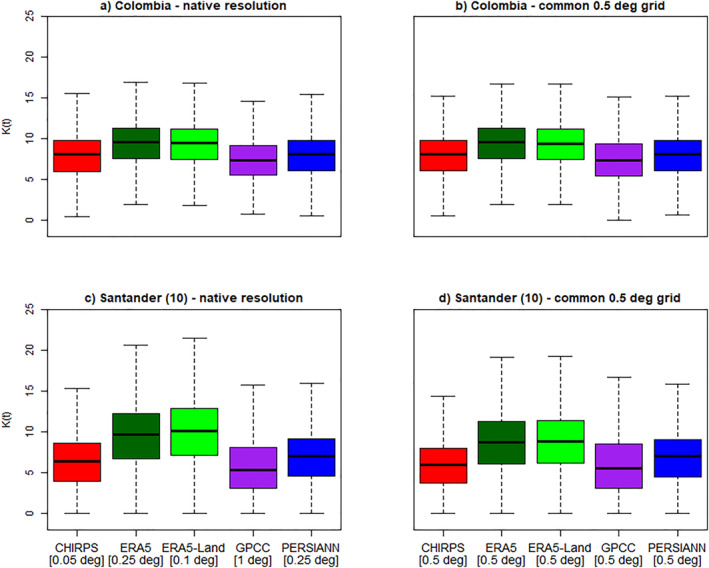
Boxplots showing the distribution of the environmental carrying capacity *K*(*t*) values derived based on daily precipitation timeseries (mm/month) for Colombia (a, b) and the COL‐VARAD1‐P Santander (10) (c, d), calculated over the base period (1991–2020). Panel (a) shows *K*(*t*) for Colombia as a whole, derived from area‐level precipitation time series based on globally gridded precipitation product (GGPP) data at its native spatial resolution, while panel (b) shows the same evaluation but based on GGPPs re‐gridded to a common 0.5° grid. Panels (c, d) display the same values for the Santander (10), with (c) representing results for GGPPs at their native resolution and (d) showing for GGPPs on a common 0.5° grid. Note that CRUTS was excluded from the analysis, as *K*(*t*) was calculated using daily values.

To illustrate the impact of observational uncertainty in a health‐related context, we focused on Colombia, specifically the densely populated region of COL‐VARAD1‐P in Santander (10), an area showing some of the most pronounced differences in precipitation estimates. The country of Colombia and this COL‐VARAD1‐P hence serve as an example to test the sensitivity of the carrying capacity, implemented as being proportional to exponentially weighted past rainfall. Figure 8 presents *K*(*t*) calculated based on different GGPPs at their native resolutions, along with a comparison to *K*(*t*) calculated using the GGPPs on a common 0.5° grid.

The five GGPPs yielded substantially different spatially averaged area‐level precipitation estimates in Colombia and Brazil, and these deviations also led to pronounced variations in the calculation of the environmental carrying capacity *K*(*t*) aligning with the expected patterns observed in Figure 7. In particular, *K*(*t*) values based on ERA5‐Land and ERA5 show a marked positive shift toward higher *K*(*t*) values across all panels compared to the other GGPPs. This shift is especially pronounced in Santander (10), where the differences between the reanalysis and all other GGPPs were particularly notable.

### 4.3 Comparison With Weather Station Data

The evaluation of GGPPs against station data followed the point‐to‐pixel approach described in Section 3.3. It can be challenging to achieve reasonable agreement using a point‐to‐pixel approach for precipitation, especially in regions with complex terrain. As demonstrated in previous sections, Colombia's orography and high precipitation contributed to substantial differences between GGPPs. This section therefore focuses on results for Colombia, with more detailed results for Brazil being provided in the Supplement. Note that differences in total precipitation affect RMSE values, which should be considered when comparing values between countries, as overall precipitation is higher in Colombia than in Brazil.

#### 4.3.1 Point‐Scale Precipitation

Station altitudes varied between 2 and 1663 m (mean 501 m) across Brazil and between 7 and 3,624 m (mean 1533 m) across Colombia. Table S5 in Supporting Information S1 shows the precipitation distributions based on aggregated monthly timeseries from each weather station in the evaluation period. The station's average for each BRA‐/COL‐VARAD1 area was calculated from the mean values of all stations within that area. In Colombia, the mean of the stations' average precipitation ranged from 11.74 mm in La Guajira (2 stations), with a min of 0 mm and a max of 239.9 mm, to 135.05 mm in Boyacá (2 stations), where the min was 0 mm and the max was 397.8 mm. For comparison, in Brazil, the mean of the average monthly precipitation across stations varied from 64.06 mm in Piauí (3 stations), with a min of 0 mm and a max of 370.8 mm, to 194.41 mm in Amazonas (5 stations), where the min was 23.2 mm and the max was 409.56 mm. Single monthly station values amounted to a max of 803.0 mm (Min87) and 580.1 mm (Nor36) in Brazil and Colombia, respectively. For the point‐level evaluation, we selected the nearest pixel for each station. Stations that needed to be excluded due to their location and lack of coverage by some GGPPs are marked with an asterisk (*) in Table S5 in Supporting Information S1.

#### 4.3.2 Comparison and Validation

**Figure 9:**
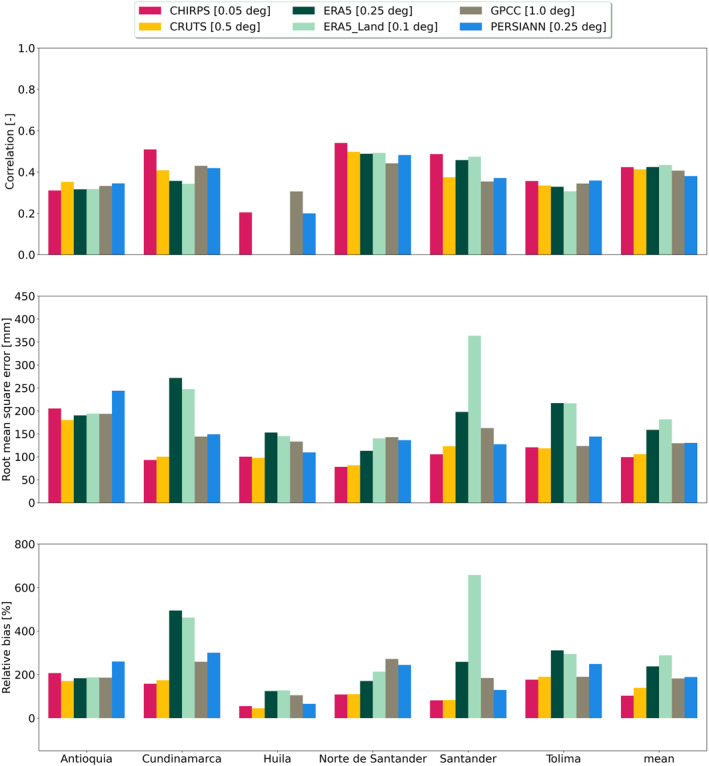
Statistical evaluation of monthly precipitation timeseries from globally gridded precipitation product (GGPP) pixels against ground observations: The evaluation presents the Pearson correlation (top), root mean square error (center), and relative bias (bottom) during the period 2011–2020 for each GGPP, averaged across each COL‐VARAD1‐P area. The COL‐VARAD1‐P areas are depicted from left to right: Antioquia (1), Cundinamarca (2), Huila (6), Norte de Santander (9), Santander (10), and Tolima (11). Mean values represent averages across all COL‐VARAD1 areas. Note that the number of weather stations evaluated varies across the different validation areas, as detailed Tables S1 and S5 in Supporting Information S1. Only stations with significant correlations were included in the analysis. GGPPs in areas with no significant point‐to‐pixel correlations are not shown (e.g., for reanalysis in Huila (6)). Results are provided for all GGPPs at their native spatial resolution.

The comparisons between station data and GGPPs across validation areas are summarized in Figure 9, as well as in Figures S18–S20 in Supporting Information S1 (including the same evaluations for GGPPs re‐gridded to a common 0.5° grid). The evaluation of each GGPP at its native resolution revealed varying degrees of agreement with station data. In Colombia, timeseries from individual stations typically exhibited a more pronounced divergence from the corresponding grid cells compared to findings in Brazil. On average, across all validation areas in Colombia, CHIRPS and ERA5‐Land showed similar PCC values (0.42 for CHIRPS and 0.43 for ERA5‐Land, with the lowest value of 0.38 seen for PERSIANN). However, CHIRPS (ERA5‐Land) had a by far lower (higher) Rbias of 102.88% (288.83%) and RMSE of 99.24 mm (181.43 mm), indicating that CHIRPS had the highest performance, while ERA5‐Land performed the worst across COL‐VARAD1 areas (see mean in Figure 9). On a common grid, GGPPs showed reduced overall agreement between point and pixel, with an increase in Rbias of approximately 5 mm for CHIRPS and 55 mm for ERA5‐Land across the validation areas (see mean in Figure S20 in Supporting Information S1). When considering areas individually, the average point‐to‐pixel agreement varied noticeably within Colombia. Among the 10 COL‐VARAD1‐P areas (with station Mag32, and thus Magdalena (8), excluded from the analysis), CHIRPS exhibited the lowest RMSE and Rbias in 6 areas. Notably, reanalysis generally showed one of the weakest agreements with station data, with the highest RMSE (Rbias) in 7 (8) VARAD1‐P regions, respectively, for either ERA5 or ERA5‐Land. For PCC, the variation between validation areas was less pronounced than the differences observed for RMSE and Rbias.

In some areas, discrepancies between reanalysis and other GGPPs were particularly pronounced. This was especially evident in Santander (10) and Cundinamarca (5), the latter of which had the highest number of weather stations. In Cundinamarca, reanalysis consistently showed the poorest performance across all metrics (with CHIRPS performing the best), while in Santander, large deviations between ERA5 and ERA5‐Land were particularly evident for RMSE and Rbias.

These findings suggest that, despite similar temporal correlation between all products and station data, reanalysis products might often exhibit a strong relative and absolute overestimation of precipitation than other GGPPs. Furthermore, in some COL‐VARAD1‐P, the impact of spatial resolution on the results was particularly evident. For example, in Santander (10), interpolation appeared to smooth out pixel‐based biases, leading to a convergence of absolute precipitation estimates from reanalysis to levels more consistent with the other GGPPs. However, in other areas such as Norte de Santander (9), RMSE and Rbias increased while PCC decreased. By comparison, in Brazil, across all VARAD1, CHIRPS and ERA5‐Land exhibited a mean Rbias of 13.02% and 14.09%, respectively, and a mean RMSE of 64.44 and 69 mm, with mean PCC values of 0.74 and 0.73 (see Figure S18 in Supporting Information S1). At daily resolution, the agreement of all products with station data decreased compared with monthly resolution (not shown). However, the overall results remained consistent across both countries, with CHIRPS being the most accurate product in most VARAD1‐P. Overall, ERA5 and ERA5‐Land were outperformed by other GGPPs.

#### 4.3.3 Spatial Variation in Performance

As anticipated, spatial patterns showed stronger agreement between monthly station data and pixel‐level estimates across Brazil than Colombia, with CHIRPS generally outperforming ERA5‐Land. In Colombia, no clear spatial pattern emerged. In Brazil, some weak spatial patterns became evident (see spatial maps of PCC, RMSE and Rbias provided in Figures S21 for Brazil in the Supporting Information S1). For example, high PCC values were clustered in Minas Gerais, Bahia, and surrounding areas for both CHIRPS and ERA5‐Land, where most stations also displayed a comparably lower RMSE and Rbias. In remote areas, such as the Amazon region, while there was generally good correlation between pixel‐level and station timeseries, GGPPs showed a pronounced relative overestimation of rainfall and large deviations in total amounts compared to local measurements, particularly pronounced in ERA5‐Land across all stations. In these areas, CHIRPS proved to be the more accurate data set, demonstrating lower systematic bias. Overall, the Amazon region is still generally characterized by poor accuracy in total precipitation estimates compared to most other regions in Brazil. Again, spatial analysis on monthly time scales generally yielded similar patterns but better GGPP performance compared with daily resolution (not shown).

### 4.4 Comparison of Area‐ and Pixel‐Level Precipitation Estimates

**Figure 10:**
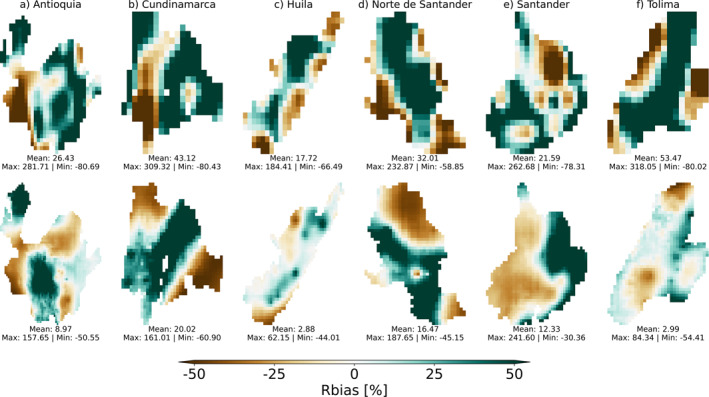
Relative bias (Rbias) derived by comparing the monthly precipitation in the base period observed at 0.1° resolution (ERA5‐Land, top) and at 0.05° resolution (CHIRPS, bottom) against the aggregated area‐level exposure estimates for each COL‐VARAD1‐P. The top and bottom rows, from left to right, show (a) Antioquia (1), (b) Cundinamarca (5), and (c) Huila (6), (d) Norte de Santander (9), (e) Santander (10), and (f) Tolima (11). Within each map, text‐based values indicate the minimum (min), mean, and maximum (max) Rbias values for each COL‐VARAD1‐P.

We mapped PCC, RMSE, and Rbias between the aggregated BRA‐VARAD1‐P and COL‐VARAD1‐P area‐level and pixel‐based timeseries for CHIRPS and ERA5‐Land (both at their native resolution). Figure 10 (Figure S24 in Supporting Information S1) shows the spatial map of Rbias between the area‐level precipitation estimates and each timeseries at every 0.1 and 0.05° pixel in ERA5‐Land and CHIRPS for the VARAD1‐P in Colombia (Brazil), respectively. Figures S22–S23 and S25–S26 in Supporting Information S1 show similar spatial maps for PCC and RMSE in Brazil and Colombia. The comparisons based on these three metrics did not directly assess the accuracy of the estimates from the two GGPPs. Instead, they focused on how well the aggregated precipitation estimates reflected pixel‐based values within each area for each GGPPs separately. However, differences between GGPPs in this context could still be analyzed. When interpreting these findings, it is important to consider the varying sizes of the areas, as shown in Table S1 in Supporting Information S1, with Brazilian VARAD1 areas generally being much larger.

The average area‐level correlation coefficients were generally high across all regions in Colombia, ranging from a mean of 0.71 (ERA5‐Land) in Cundinamarca (5) to 0.89 (CHIRPS) in Tolima (11). However, within a COL‐VARAD1‐P, some areas exhibited lower correlations, for example, notably in southern Huila (6), for both GGPPs. The evaluation of Rbias and RMSE indicated that aggregated estimates often struggled to accurately capture the spatial distribution of precipitation. The area‐averaged Rbias values ranged from a mean of 2.88% (CHIRPS) in Huila (6) to 53.47% (ERA5‐Land) in Tolima (11). Similarly, the mean area‐averaged RMSE values varied from 43.20 mm (CHIRPS) in Huila (6) to 170.75 mm (ERA5‐Land) in Tolima (11). In general, CHIRPS demonstrated superior performance for each COL‐VARAD1‐P, based on both RMSE and Rbias, particularly in areas such as Huila (6) and Tolima (11). This is consistent with ERA5‐Land showing greater spatial variability and notable biases in some pixels compared to other GGPPs (see Section 4.1.1. for details).

For comparison, similar area‐averaged correlation values were observed in Brazil, with PCC values ranging from a mean of 0.75 (CHIRPS) in Amazonas (1) to a man of 0.93 (ERA5‐Land) in Rio de Janeiro (15) and São Paulo (19). Respective area‐averaged RMSE values in Brazil ranged from a mean of 39.01 mm (CHIRPS) in São Paulo (19) to a mean of 72.01 mm (CHIRPS) in Amazonas (1), while respective Rbias values ranged from a mean of 1.47% (ERA5‐Land) in Amazonas (1) to a mean of 13.13% (CHIRPS) in Bahia (2). Overall, mean area‐averaged Rbias and RMSE values, were in general hence lower in Brazil than in Colombia, with the GGPPs showing closer agreement across BRA‐VARAD1‐P. Thus, despite VARAD1 areas being larger in Brazil, more spatially homogeneous rainfall patterns seemed to allow area‐level estimates to better capture localized total precipitation.

Results indicate that areas with consistently high correlations displayed regionally homogeneous precipitation variability, often influenced by large‐scale atmospheric drivers rather than localized factors. Conversely, areas with lower and spatially diverse correlations showed more localized precipitation variability, with complex spatial patterns often shaped by orographic effects. Regions with notable discrepancies in Rbias and RMSE frequently overlap with zones of lower correlation; however, significant relative biases and absolute differences between aggregated and pixel‐based values were also observed in areas with high PCC values. Similar patterns were observed for daily timeseries (not shown), however, PCC (RMSE/Rbias) decreased (increased) with higher temporal resolution.

## 5 Discussion

This study evaluated six state‐of‐the‐art GGPPs across South America, offering insights into the performance of each GGPP in disease‐relevant, complex climatic and topographic settings. We selected interpolated data sets, satellite‐based products, and reanalysis products to provide a comprehensive statistical comparison and utility evaluation for health applications and impact assessments. Our main objective was to determine whether different types of products give similar aggregated area‐level precipitation estimates, as commonly used in environmental epidemiology, across diverse regions, with a particular focus on a comparison between reanalysis and CHIRPS. We examined differences between GGPPs and how these affected area‐level precipitation estimates in inhabited regions, where accurate precipitation estimates are particularly essential for health studies. Our analysis focused on Brazil and Colombia because of their heterogeneity in size, population density, orography, and rainfall patterns. Both countries represent climatically, ecologically, and epidemiologically important regions for (tropical) disease and health studies.

### 5.1 Pixel‐Based Differences in GGPPs

Overall, the most prominent climatological features and major precipitation patterns in the South American study domain, including those associated with the ITCZ, were consistently represented across all GGPPs. However, a range of comparative analyses focusing on geographic distributions, extreme precipitation indices, and indicators relevant for species distribution modeling, revealed distinct regional variations in GGPP estimated precipitation when compared to CHIRPS as reference. While GGPPs demonstrated strong agreement in certain areas, such as inner‐continental regions (e.g., northwestern Brazil), pronounced differences became evident in Andes‐dominated regions and coastal areas, and generally in zones typically characterized by extreme humidity or aridity.

While relative differences from CHIRPS were evident across all GGPPs in various regions of South America, ERA5 and ERA5‐Land stood out, exhibiting the most spatially extensive and pronounced wet and dry biases in large parts of the study domain. Reanalysis products especially faced challenges to accurately represent precipitation in western mountainous regions, coastal zones, and extreme humid and arid areas. The inconsistencies observed in GGPPs impacted all evaluated indices (ETCCDI and BCVs), indicating that these biases stem from factors beyond systematic errors or constant offsets. Our findings specifically highlight the limitations of reanalysis in accurately representing rainfall distributions and characteristics. For example, ERA5 and ERA5‐Land not only overestimated precipitation (relative wet bias) over large areas of western South America but also showed an enhanced frequency of CWD and heavy precipitation days (R20 mm) in these areas. Despite this overestimation, the total precipitation amounts for wet days (SDII) in reanalysis were generally comparable to, or slightly lower than, those from CHIRPS.

Notably, there was no large fundamental difference between ERA5 and ERA5‐Land, as ERA5‐Land precipitation rates are interpolated from ERA5 onto a finer grid. The pronounced inconsistencies in reanalysis products compared to CHIRPS and other GGPPs might arise for example, from differences in production methods, incorporated data sets, data collection and assimilation schemes, and underlying model structure. Identifying which factors contributed most to GGPP inconsistencies remains challenging; however, our findings suggest that ERA5 and ERA5‐Land need to be evaluated with care before utilization in health‐related impact assessments and reanalysis products might not be suitable for all analyses or applications, especially in certain geographic regions. For instance, our analysis showed relatively moderate differences among GGPPs in Brazil, while in contrast, Colombia, representing one of the world's wettest countries, presented a particular challenge for reanalysis, due to its complex terrain and localized and large precipitation variability.

### 5.2 Evaluation of GGPPs Using Weather Station Data

When comparing GGPPs against ground‐based station data, our analysis showed that CHIRPS and reanalysis‐based data sets exhibited the highest and lowest performance in Brazil and Colombia, respectively. The results for both countries thus confirmed previous evaluations of data set quality products (see the Introduction and references for further details). The agreement between pixel‐based data and station observations was notably higher for Brazil than for Colombia. Since pixels values are themselves relatively large area estimates (depending on the specific grid resolution of the product), they may not accurately capture conditions in the local vicinity of a weather station. Therefore, it is unsurprising that Colombia, which includes many regions characterized by high spatial variability in rainfall, exhibited less overall point‐to‐pixel agreement when compared to a country like Brazil with more homogenous patterns.

### 5.3 Impact of Spatial Aggregation and Sensitivity Analysis

The inconsistencies in GGPPs, particularly the significant and fundamental differences between reanalysis and CHIRPS, were evident not only in the spatial analysis and evaluation of the gridded products (including their validation with ground‐based station data), but also in the examination of aggregated precipitation estimates derived from GGPPs at both country and administrative unit levels. Spatial variation in GGPPs influenced derived precipitation timeseries; despite the relatively high spatial resolutions of 0.25° and 0.1° for ERA5 and ERA5‐Land, timeseries from other GGPPs with lower native resolutions often displayed more similar distributions and only minor differences in climatological annual cycles compared to CHIRPS. This effect was noticeable even when averaging over entire countries, as observed in Colombia. In our evaluation of respective validation areas, differences were particularly pronounced in regions influenced by factors such as terrain, size, or the presence of water bodies. Additionally, the size of administrative units affected the results, with smaller areas, for which a smaller number of pixels are generally used for spatial aggregation, generally showing larger differences between GGPPs.

As an illustrative example, we demonstrated that observational uncertainty impacts the rainfall‐dependent vector carrying capacity equation, a standard approach to incorporating the influence of precipitation in vector‐borne disease research, particularly malaria. This simple and practical example highlighted the importance of considering the impact of different GGPPs, as they strongly influence modeled capacity. We hence anticipate that, in the end, they might also strongly contribute to uncertainty in the simulated malaria disease risk.

Overall, our findings suggest that the accuracy of GGPPs is the primary factor determining their suitability for health applications and impact assessments, rather than horizontal grid resolution, which is often a key criterion for data set selection in impact studies. This is especially true in regions characterized by pronounced localized temporal and spatial precipitation variability. Although the impact of GGPP resolution–whether aggregation used GGPPs at native resolution or re‐gridded to a common grid–was apparent in our comparative analysis, no consistent pattern emerged. This further suggests that resolution, familiarity with the data provider, or general acceptance and widespread use of a GGPP should not be the primary criteria for data set selection.

### 5.4 Local Representativity of Area‐Level Estimates

Spatial aggregation of GGPP pixel‐level estimates up to administrative unit level risks by averaging over areas with very different precipitation patterns, can potentially reduce the representativeness of the area‐level estimates. While spatial aggregation may be necessary for a specific application–depending on the health application or impact model considered–it comes with the obvious disadvantage of losing representativity of local conditions, as spatially aggregated precipitation estimates cannot capture variations below the scale of aggregation. In regions with spatially homogeneous rainfall patterns (for instance, because of the influence of a large‐scale driver), aggregated estimates may still provide useful inputs for impact assessments and health applications. However, in areas with heterogeneous rainfall, aggregation can lead to substantial losses in local representativity, limiting the utility of area‐level estimates for certain analyses and applications. For example, while high correlations were observed in many VARAD1‐P of Brazil and Colombia for CHIRPS and ERA5‐Land, area‐level averages could not capture localized variations, particularly in areas with pronounced spatial heterogeneity.

Therefore, the appropriate scale of aggregation for a given application should consider both the health impact in question and the “regime” of spatial variability, referring to the degree of local precipitation variability. Evaluating the representativeness of area‐level precipitation estimates with respect to spatial variations is essential before using GGPP in scientific applications. This is essential to avoid overly simplistic assessments of health outcomes that may be shaped by conditions at finer spatial scales than the aggregation level, particularly in regions where area‐level precipitation estimates showed poor skill in capturing localized variations. However, these variations and local conditions can significantly impact specific health outcomes.

## 6 Limitations

Some of our results may be influenced by limitations that should be acknowledged. To start with, administrative units are not fixed entities–boundaries change over time. Consequently, the presented results should be interpreted in the context of the version of GADM utilized. This limitation should be taken into account when interpreting our findings, particularly when applying them over time or comparing them across different studies, as changes in boundaries could affect the analysis and the conclusions drawn from the data.

Limitations might arise from the selection of weather stations used for our data set evaluation, also setting the focus of our study on specific administrative units in Brazil and Colombia. First, the validation of GGPPs against stations, the core element of our data set evaluation, is fraught with difficulties. Ground‐based observations are limited to a few points in space and time. GGPP performance might vary in locations where our analysis lacked site‐specific information. Second, grid cells represent area estimates that depend on the specific grid resolution of each GGPP and, therefore, do not reflect point‐scale conditions in the local vicinity of a weather station. While this generally affects our evaluation, the point‐to‐pixel analysis was conducted on a native and common 0.5° grid–the former to evaluate data set suitability for applied research that exclusively incorporates GGPPs at their native spatial resolution, and the latter to improve our simple data set quality assessment by minimizing the impact of varying spatial resolution. Third, weather stations are also commonly included in the generation of various GGPPs (including CHIRPS and GPCC–refer to Text S1 in Supporting Information S1), albeit ground‐based stations are not consistently incorporated into the development of most GGPPs. However, any overlap between our choice of stations and those used to develop various GGPPs would limited the independence and statistical validity of our validation analysis.

In this context, our results could have been in general affected by temporal and spatial inconsistencies in the provided GGPPs. We did not investigate how temporal inconsistencies in the development of GGPPs–such as the significant differences in the incorporated station data within gauge‐based products over time, as described for GPCC–impact the reliability and accuracy of these data sets, which is crucial for long‐term climate analysis and impact assessments. Evidence suggests that limited station data can significantly affect the performance of various gridded precipitation products in complex terrains. Many GGPP rely on observation networks, leading to increased errors during periods of low data availability and highlighting the need to assess the temporal patterns of integrated observation data. Additionally, we did not explore the impact of lakes and other water bodies on precipitation estimates across GGPPs, especially important in areas with mixed land and water surfaces. However, for example, CHIRPS includes precipitation data over lakes and other water bodies, whereas ERA5‐Land excludes them due to its land‐sea mask, which only considers land areas. This discrepancy might lead to differences in precipitation estimates, particularly in regions with large lakes or coastal areas where precipitation patterns are strongly influenced by nearby water bodies.

Last, while GGPPs can be a valuable alternative to weather stations, especially in regions with sparse monitoring networks, caution is needed when transferring results from our validation areas to regions with limited data coverage. In such regions, GGPPs‐estimated precipitation may exhibit greater bias compared to areas with more extensive monitoring. However, we argue that our assessment went beyond a simple comparison of precipitation data sources against ground‐based data. We specifically evaluated the performance of GGPPs for health‐related impact assessments, which typically focus on inhabited regions. The selected weather stations in both Brazil and Colombia were located in or near densely populated areas. This made the validation areas meteorologically and epidemiologically relevant, as they represent regions where human populations and disease vectors, such as mosquitoes that transmit infections, interact closely and are affected by the surrounding environment. Thus, while our findings may not be fully independent or universally applicable to all regions, we believe they are robust and informative for health‐related impact assessments, as our evaluation targeted areas of specific relevance to health studies.

## 7 Summary and Conclusion

The evaluation of six (quasi‐) global gridded precipitation products revealed pronounced differences in the accuracy of spatial precipitation estimates. While GGPPs can be promising alternatives to ground‐based weather station data, differences in GGPPs translated into variations in spatially aggregated area‐level precipitation estimates commonly used in health studies. Given our analysis, such differences are likely to affect modeled health outcomes, especially if impact models and their underlying assumptions are sensitive to data input. Our findings suggest that CHIRPS is the most suitable product for climate impact assessments and health applications in tropical regions. Conversely, the performance and utility of reanalysis‐based products in tropical regions were found to be poor, suggesting that alternative products are better suited for decision‐making and health‐related climate services.

Researchers and practitioners utilizing publicly available precipitation products, whether model‐based, satellite‐derived, or interpolated, should be aware of each product's specific strengths and limitations in both analysis and interpretation of results. It is crucial to carefully evaluate and select a GGPP before applying it in health research and impact assessments, as well as to test the sensitivity of each impact model to data input. This especially recommended for research in regions with complex terrain, including areas with significant altitude variations, as well as coastal, mountainous, and regions characterized by extreme humidity or aridity. Our findings and recommendations are in general valuable to infectious disease modelers and epidemiologists working on climate impact assessments, as well as practitioners engaged in broader hydrological and climate change impact analyses, particularly those focused on administrative or geographical units.

## Conflict of Interest

The authors declare no conflicts of interest relevant to this study.

## Supporting information

Supporting Information S1

## Data Availability

All data sets utilized for the analysis and presented in this manuscript are publicly available. The ERA5 (representing the fifth generation ECMWF reanalysis) and ERA5‐Land (based on replaying the land component of the ECMWF ERA5 climate reanalysis) data sets were downloaded from the Copernicus Climate Change Service available at Hersbach et al. (2023) and Muñoz Sabater (2019). CHIRPS (version 2.0, global_daily, p05) and PERSIANN‐CDR (full globe, daily) data are available at Climate Hazards Group (2015) and at Center for Hydrometeorology and Remote Sensing (2020). CRUTS (version 4.07) is available at Climate Research Unit (2023) and GPCC (Full Data Daily Version 2022) is available at Global Precipitation Climatology Centre (2022). INMET station data were downloaded at Instituto Nacional de Meteorologia (2024) and IDEAM (Instituto de Hidrología & Meteorología & Estudios Ambientales, 2024) provided station data upon request (contacto@ideam.gov.co, enquiries received on 29‐01‐2024 and 09‐02‐2024). We downloaded spatial data of the GADM database (version 4.1) at GADM (2018‐2022) as well as extracted GPWv4 (revision 11) population data at Socioeconomic Data and Applications Center (2018). Most of the data download, processing, and preparation were conducted using the R statistical (version 4.3.2, IDE RStudio) and the Python (Python 3.11.7, IDE PyCharm) programming languages as well as Climate Data Operators (CDO) (Schulzweida et al., 2009), based on the provided scripting language package for Python (which is a wrapper around the CDO binary).
