# Supplementary material for: Evaluation of the Performance and Utility of Global Gridded Precipitation Products for Health Applications and Impact Assessments in South America
Source: Geohealth. 2025 Jun 18;9(6):e2024GH001260. doi: 10.1029/2024GH001260 (PMC12174871; doi:10.1029/2024GH001260)
Supplement: Supplementary file 1 — Supporting Information S1 [file GH2-9-e2024GH001260-s001.docx]

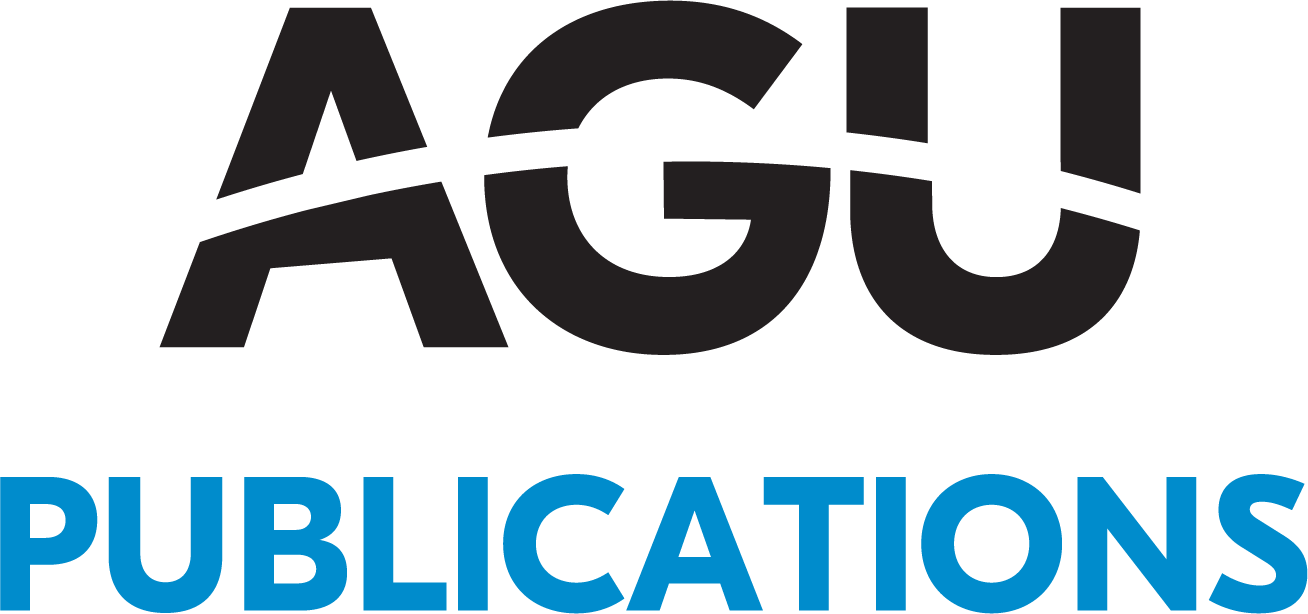


*GeoHealth*

Supporting Information for

**Evaluation of the Performance and Utility of Global Gridded Precipitation Products for Health Applications and Impact Assessments in South America**

Sally Jahn^1^, Katy A. M. Gaythorpe^1^, Caroline M. Wainwright^2^ and Neil M. Ferguson^1^

^1^MRC Centre for Global Infectious Disease Analysis, School of Public Health, Imperial College London

^2^University of Leeds, Leeds, United Kingdom

Corresponding author: Sally Jahn (s.jahn@imperial.ac.uk)

**Contents of this file**

Text S1 to S2

Figures S1 to S26

Tables S1 to S5

**Text**

**Text S1. Global Gridded Precipitation Products (GGPPs)**

The different GGPPs used in our analysis are briefly summarized below. More detailed descriptions of each product can be found in the respective references listed in Table 1.

1. **Interpolated Datasets**

The CRUTS dataset, provided by the Climatic Research Unit (CRU) at the University of East Anglia, includes various climate variables, such as precipitation, and covers Earth's land areas. This dataset is generated by interpolating monthly climate anomalies from extensive weather station networks using angular-distance weighting (Harris et al., 2020). The dataset integrates data from over 4000 weather stations into a consistent format. While many inputs have been homogenized, the dataset is not fully homogeneous. Note that the global dataset is only available and was downloaded at a monthly resolution.

The Global Precipitation Climatology Centre, operated by the German Weather Service (DWD), provides a dataset of daily totals of global land-surface precipitation. This GPCC dataset is based on data from around 80,000 stations worldwide, sourced from national meteorological and hydrological services, global and regional data collections, and WMO GTS data (Ziese et al., 2022). Coverage varies over time, leading to potential inhomogeneities. Relative precipitation anomalies at the stations are interpolated and then superimposed on the respective full monthly GPCC product. The global dataset was available for download at a daily resolution.

1. **Reanalysis**

The ERA5 and ERA5-Land datasets are documented and freely available from the Copernicus Climate Data Storage provided by the European Centre for Medium-Range Weather Forecasts (ECMWF) (ECMWF & Copernicus, 2023). These datasets include a variety of atmospheric variables, such as temperature, humidity as well as other variables such as snow cover. For generating spatially resolved reanalysis, a data assimilation scheme and corresponding models are utilized, hence incorporating modeled forecasts corrected by observations to provide historical gridded estimates across the full geographic extent.

ERA5 is based on four-dimensional variational (4D-Var) data assimilation using Cycle 41r2 of the Integrated Forecasting System (IFS), which is coupled to a soil model and an ocean wave model (Hersbach et al., 2020). ERA5-Land, which covers only land surfaces, is an enhanced reprocessing of the land component using a higher resolution model version and taking ERA5 as an input, with missing values explicitly marked (Muñoz-Sabater et al., 2021). Precipitation data from ERA5 and ERA5-Land (on singe levels) were downloaded at their native spatial resolution and on an hourly time step.

1. **Satellite-based Products**

The Precipitation Estimation from Remotely Sensed Information using Artificial Neural Networks-Climate Data Record (PERSIANN-CDR) is a retrospective precipitation dataset developed by the National Climate Data Center (NCDC) of the National Oceanic and Atmospheric Administration NOAA and the Center for Hydrometeorology and Remote Sensing (CHRS) at the University of California, Irvine. This dataset provides global precipitation estimates by integrating high-quality passive microwave (PMW) and high-frequency infrared (IR) observations from both low Earth orbit and geostationary satellites (Ashouri et al., 2015). The PERSIANN algorithm uses an artificial neural network (ANN) and is applied to maintain high spatial resolution over four decades, especially addressing gaps in PMW data prior to 1997. The GPCP monthly product is employed to correct rain-rate estimates and align them with the GPCP data, introducing some dependency on it. We aggregated daily estimates from the provided and downloaded sub-daily dataset.

Climate Hazards Group InfraRed Precipitation with Station data (CHIRPS) is a quasi-global dataset with nearly 40 years of data. It merges satellite-based rainfall estimates with gauge-based observations, incorporating CHC’s climatology (CHPclim), to provide a gridded timeseries (Funk et al., 2015; Funk et al., 2014). CHPclim uses over 27,000 monthly stations from the Agromet Group of the Food and Agriculture Organization of the United Nations (FAO) and approximately 21,000 stations from the Global Historical Climate Network (GHCN) to construct CHPclim. The updated second version of CHIRPS offers an improved daily rainfall timeseries, incorporating more and additional contemporary station observations from global initiatives such as the daily GTS archive from NOAA’s Climate Prediction Center CPC, along with additional data collected from national and sub-national sources. Global data were available for download at a daily resolution.

**Text S2. Evaluation Metrics**

We applied common statistical methods, such as the Pearson Correlation Coefficient (PCC), Relative Bias (Rbias), and Root Mean Square Error (RMSE), to evaluate e.g., each individual GGPP (P) against station observations (O), which were considered the ground truth. More information on the metrics is provided in the examples below.

1. Pearson correlation coefficient (PCC): Values range between −1 and +1. A value of PCC close to +1 showed a perfect positive fit and linear relationship between GGPP and field-based station data. Correlations were only provided where they were statistically significant at the 95% level.

$$PCC=\frac{\sum_{i=1}^{N} (P_{i}-\bar{P})\times(O_{i}-\bar{O})}{\sqrt{{\sum_{i=1}^{N} (P_{i}-\bar{P})}^{2}}\times\sqrt{{\sum_{i=1}^{N} (O_{i}-\bar{O})}^{2}}}$$

2. Bias and Relative bias (Rbias): The values depicted the average differences and systematic bias of each GGPP in comparison to the station data. Bias and Rbias can be positive (overestimation) or negative (underestimation).

$$Bias=\frac{\sum{(P_{i}-O}_{i})}{N}$$

$$R_{Bias}=\frac{\sum_{i=1}^{N} (P_{i}-O_{i})}{\sum_{i=1}^{N} O_{i}}\times100$$

3. Root mean square error (RMSE): The RMSE is a well-established and accepted indicator of goodness of fit, i.e., it indicates the accuracy of modelled or estimated values. In this context, RMSE evaluated how closely the values of a GGPP matched the ground observations.

$$RMSE=\sqrt{\frac{{\sum_{i=1}^{N} (O_{i}-P_{i})}^{2}}{N}}$$

**Figures**


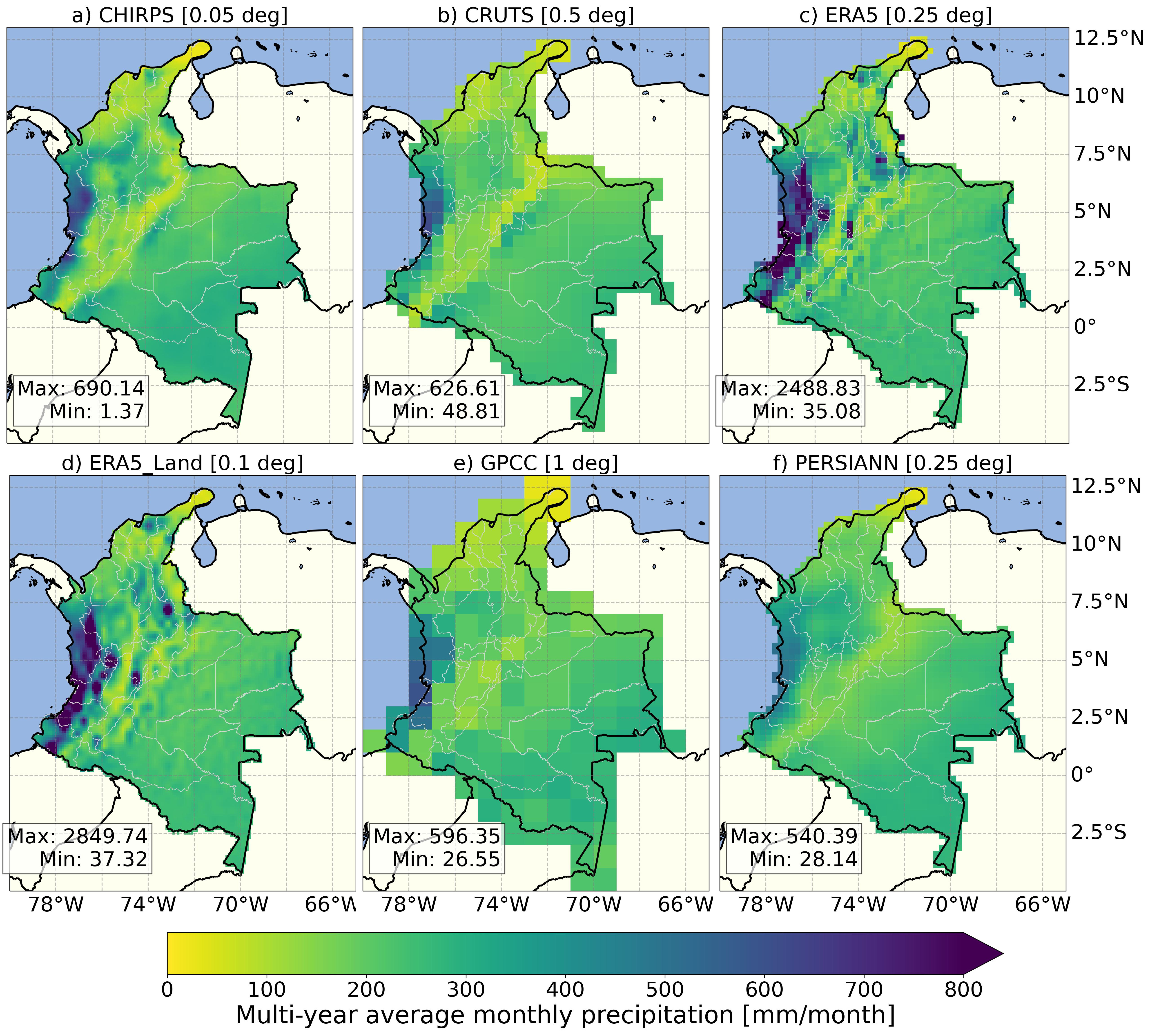
**Figure S1.** Multi-year average monthly precipitation [mm/month] across Colombia for each GGPP (a-f), averaged over the base period (1991-2020). The top row presents CHIRPS (a), CRUTS (b), and ERA5 (c), while the bottom row features ERA5-Land (d), GPCC (e), and PERSIANN (f). The maps display precipitation data at their native spatial resolution. Within each map, boxes indicate the pixel-based minimum (min) and maximum (max) precipitation values for each GGPP across Colombia.


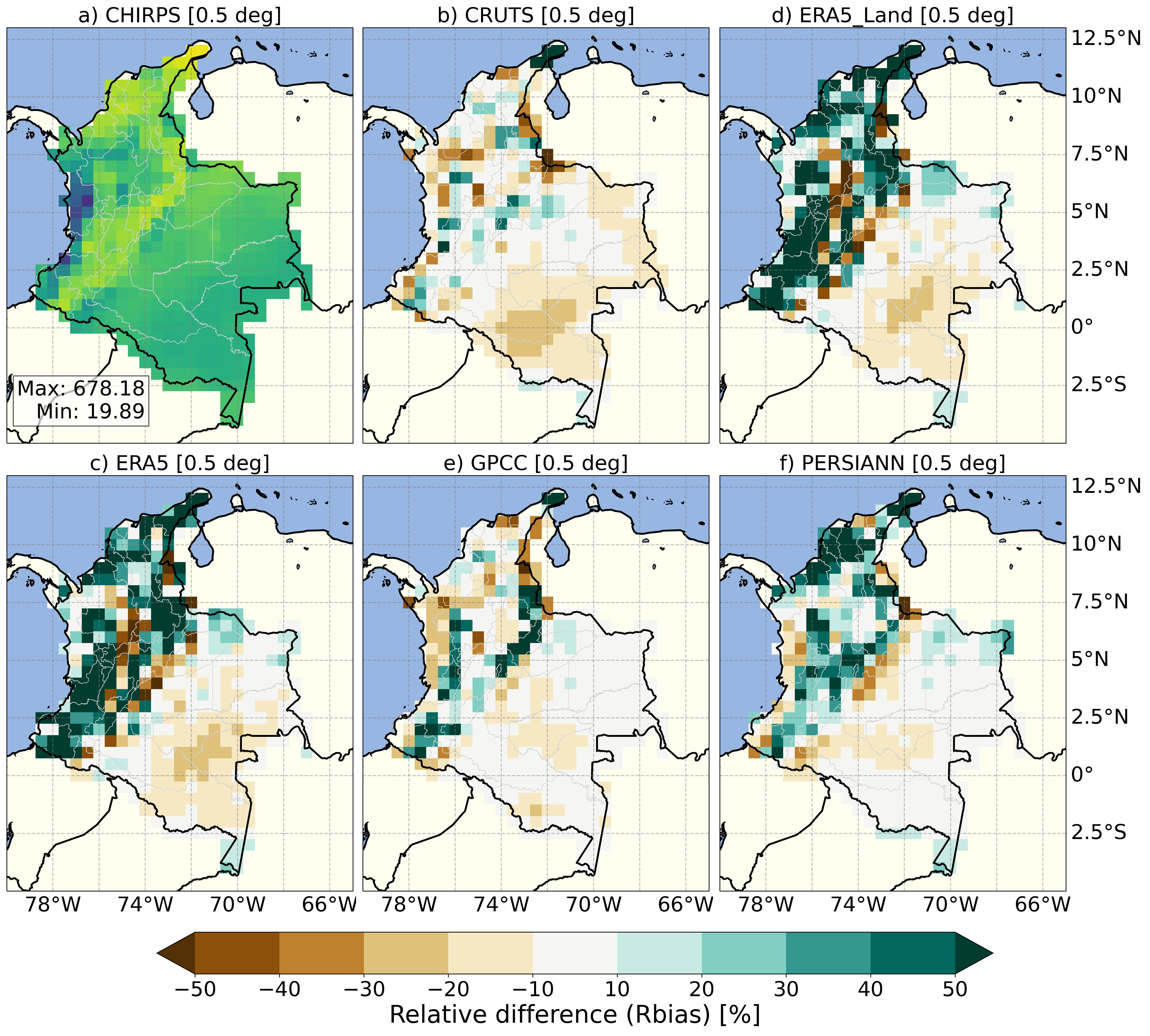
**Figure S2.** Multi-year average monthly precipitation [mm/month] for CHIRPS (a, legend as in Figure S1) and the relative difference (Rbias in %) from CHIRPS for all other GGPPs (b-f), averaged over the base period (1991-2020), across Colombia. The top row shows CHIRPS (a), CRUTS (b), and ERA5 (c), while the bottom row displays ERA5-Land (d), GPCC (e), and PERSIANN (f). All maps are presented at a common 0.5 deg spatial resolution.


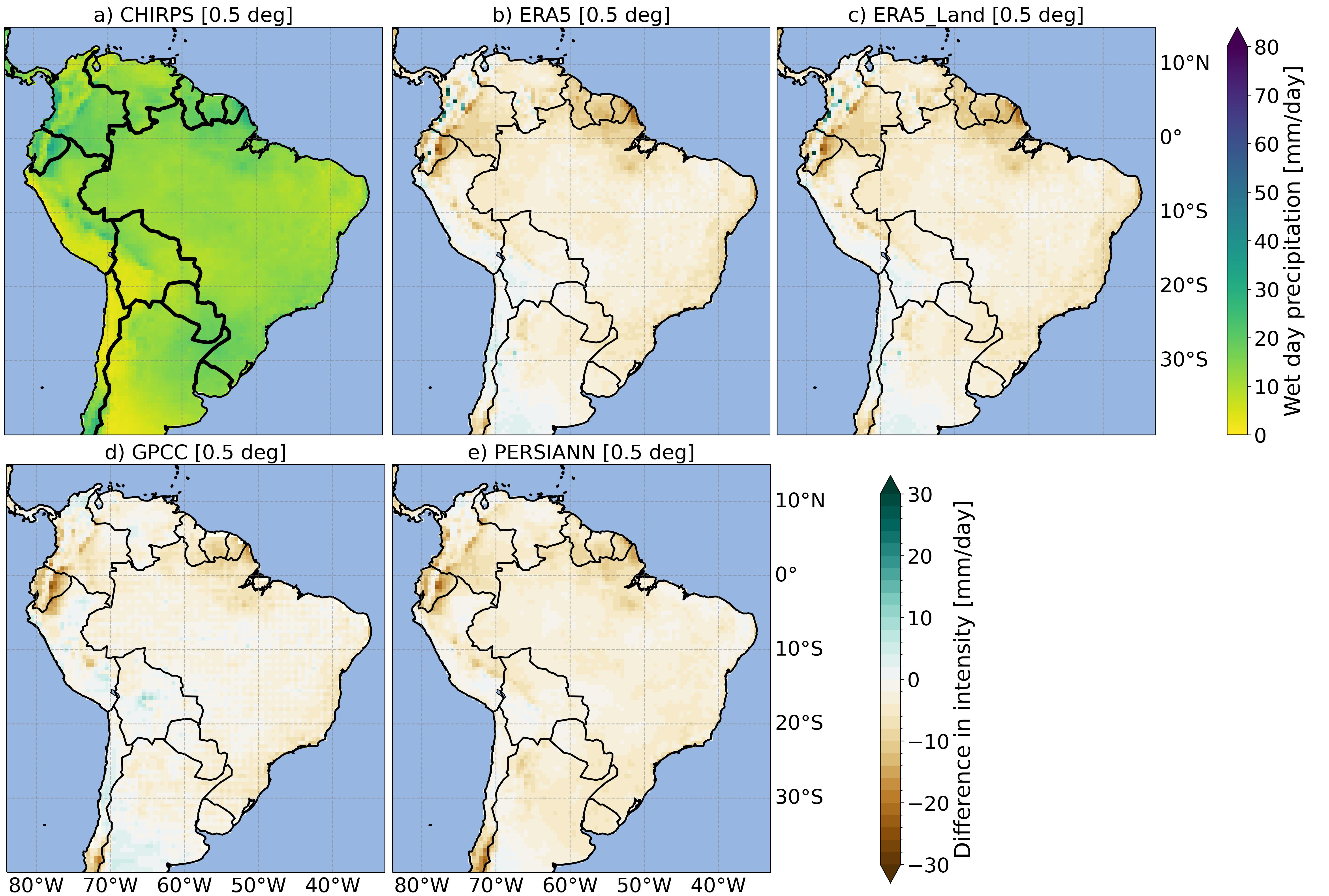
**Figure S3.** SDII - the mean precipitation amount [mm/day] on wet days for CHIRPS (a). For all other GGPPs (b-e), the difference [mm/day] compared to CHIRPS is shown. All values represent averages over the base period (1991-2020). Note that CRUTS was excluded from the analysis, as SDII was calculated using daily values. The top row includes CHIRPS (a), ERA5 (b), and ERA5-Land (c), while the bottom row shows GPCC (d), and PERSIANN (e). The maps are presented on a common 0.5 deg grid.


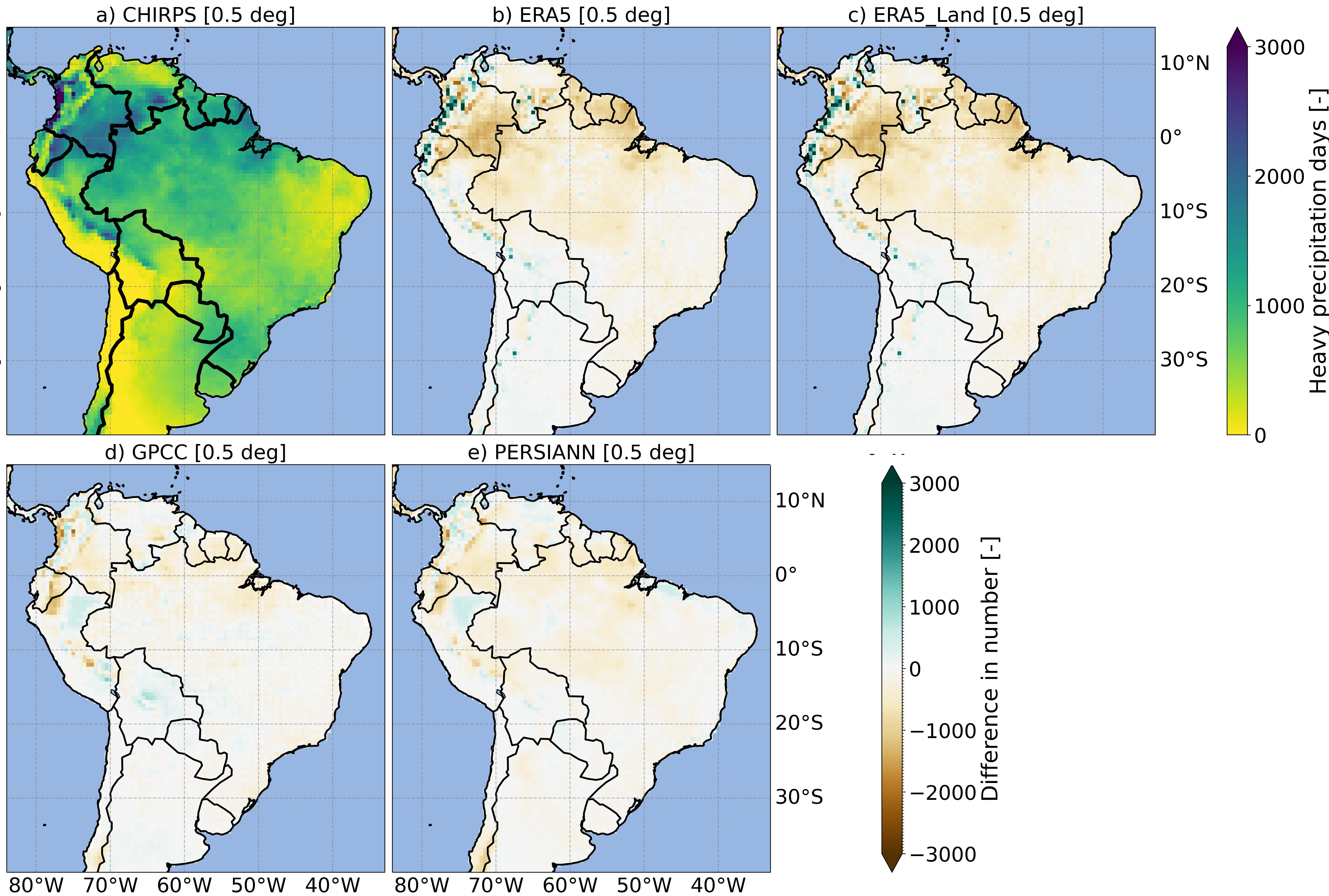
**Figure S4.** R20mm - the number of days [-] with precipitation exceeding 20 mm for CHIRPS (a). For all other GGPPs (b-e), the difference [-] compared to CHIRPS is shown. All values represent averages over the base period (1991-2020). Note that CRUTS was excluded from the analysis, as R20mm was calculated using daily values. The top row includes CHIRPS (a), ERA5 (b), and ERA5-Land (c), while the bottom row shows GPCC (d), and PERSIANN (e). The maps are presented on a common 0.5 deg grid.


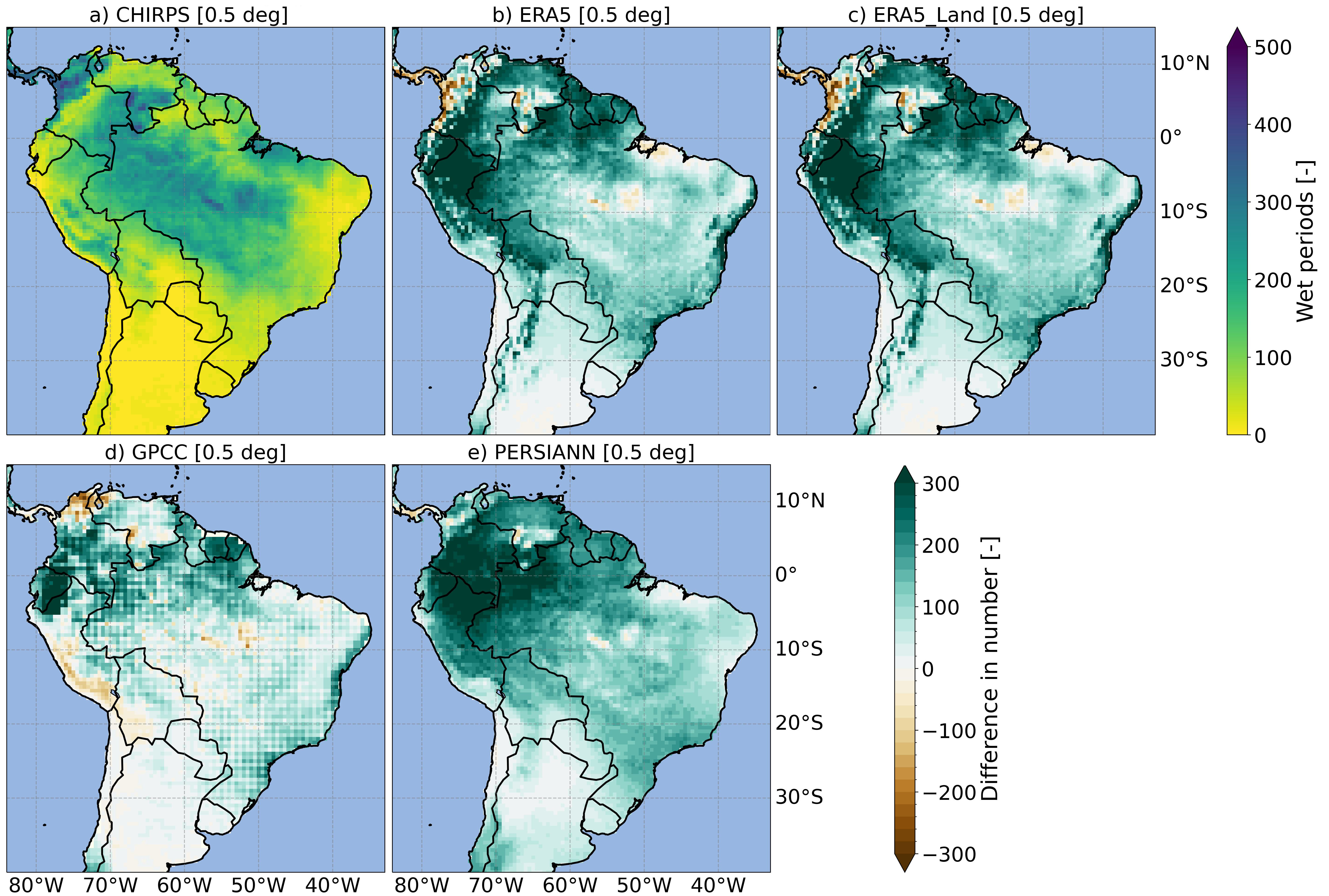
**Figure S5.** CWD - the number of wet periods lasting more than 5 days [-] for CHIRPS (a). For all other GGPPs (b-e), the difference [-] compared to CHIRPS is shown. All values represent averages over the base period (1991-2020). Note that CRUTS was excluded from the analysis, as CWD was calculated using daily values. The top row includes CHIRPS (a), ERA5 (b), and ERA5-Land (c), while the bottom row shows GPCC (d), and PERSIANN (e). The maps are presented on a common 0.5 deg grid.


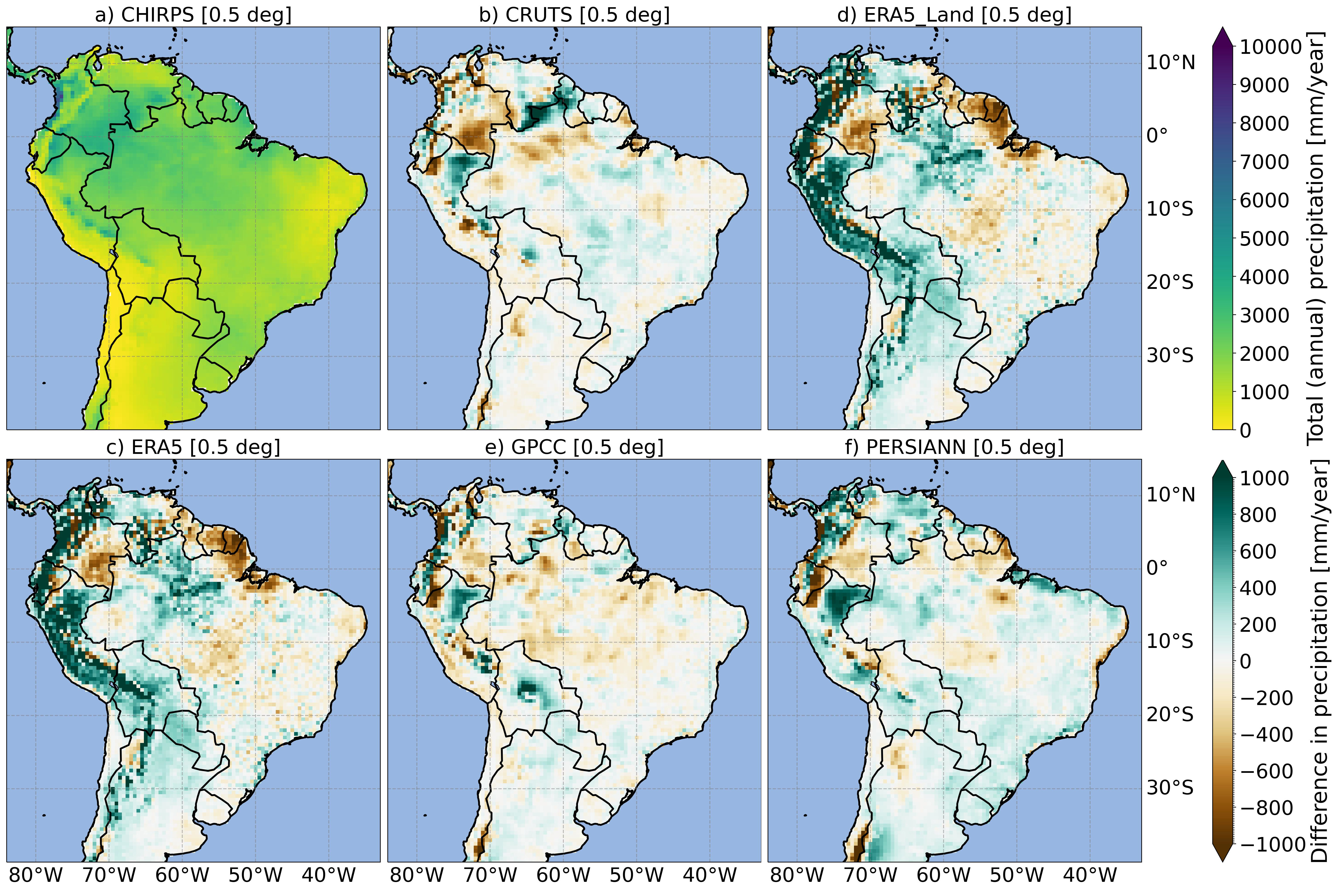
**Figure S6.** BCV12 - the total annual precipitation [mm/year] of CHIRPS (a). For all other GGPPs (b-f), the difference [mm/year] from CHIRPS is shown. All values are based on climatological monthly averages calculated over the base period (1991-2020). The top row shows CHIRPS (a), CRUTS (b), and ERA5 (c), while the bottom row displays ERA5-Land (d), GPCC (e), and PERSIANN (f). The maps are presented on a common 0.5 deg grid.


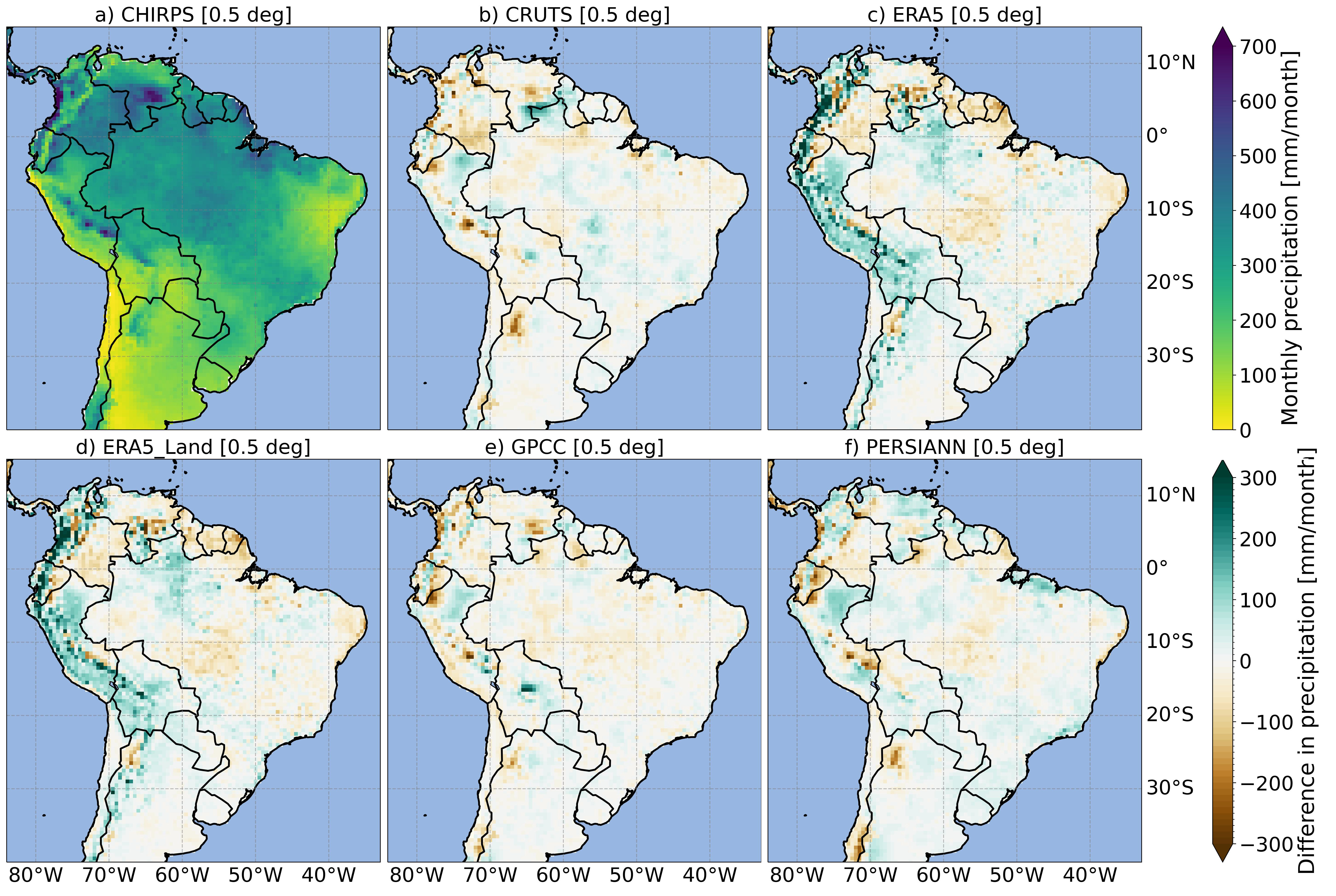
**Figure S7**. BCV13 - the total precipitation [mm/month] of CHIRPS (a) of the wettest month and, for all other GGPPs (b-f), the difference [mm/month] from CHIRPS is shown. All values are based on climatological monthly averages calculated over the base period (1991-2020). The top row shows CHIRPS (a), CRUTS (b), and ERA5 (c), while the bottom row displays ERA5-Land (d), GPCC (e), and PERSIANN (f). The maps are presented on a common 0.5 deg grid.


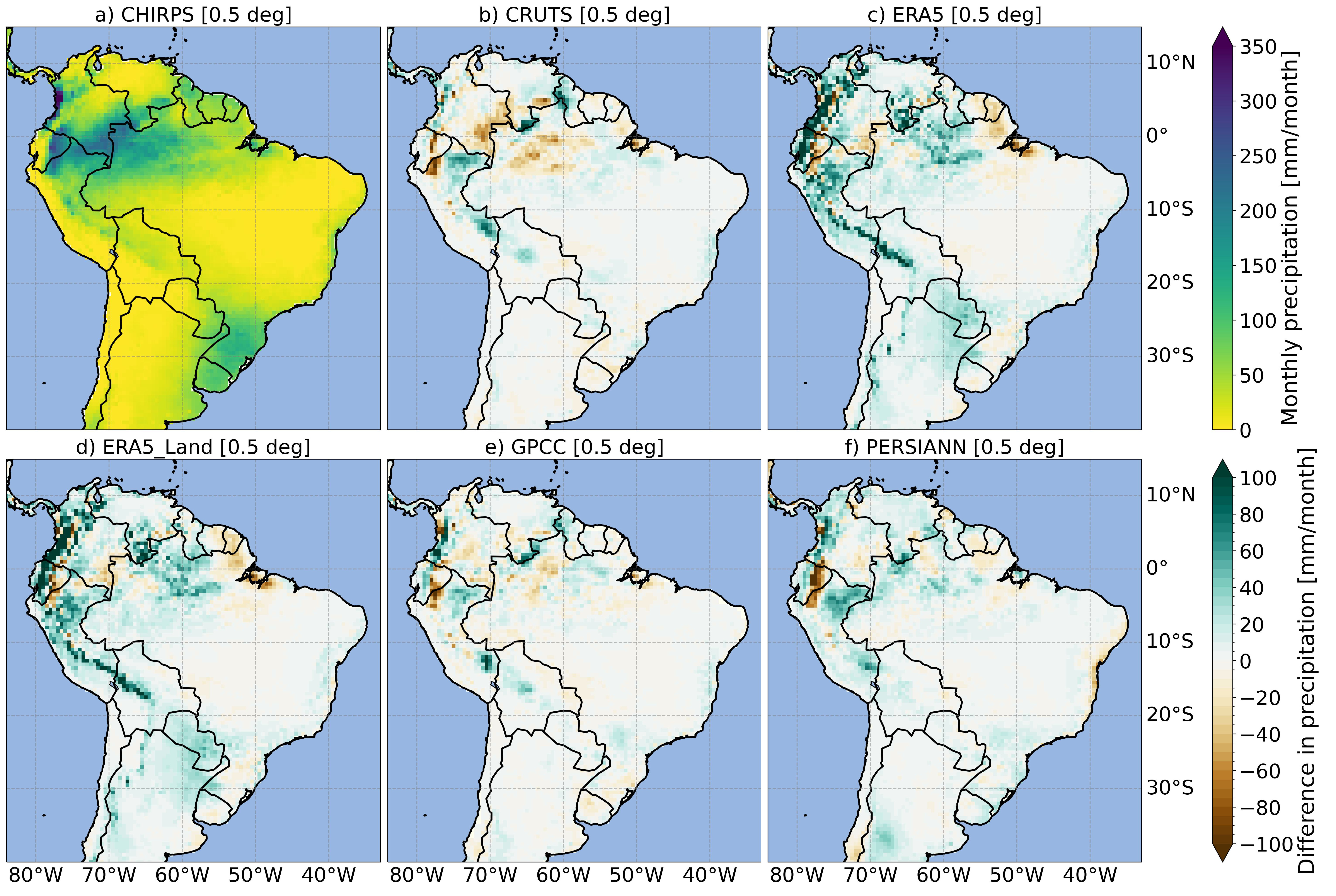
**Figure S8.** BCV14 - the total precipitation [mm/month] of CHIRPS (a) of the driest month and, for all other GGPPs (b-f), the difference [mm/month] from CHIRPS is shown. All values are based on climatological monthly averages calculated over the base period (1991-2020). The top row shows CHIRPS (a), CRUTS (b), and ERA5 (c), while the bottom row displays ERA5-Land (d), GPCC (e), and PERSIANN (f). The maps are presented on a common 0.5 deg grid.


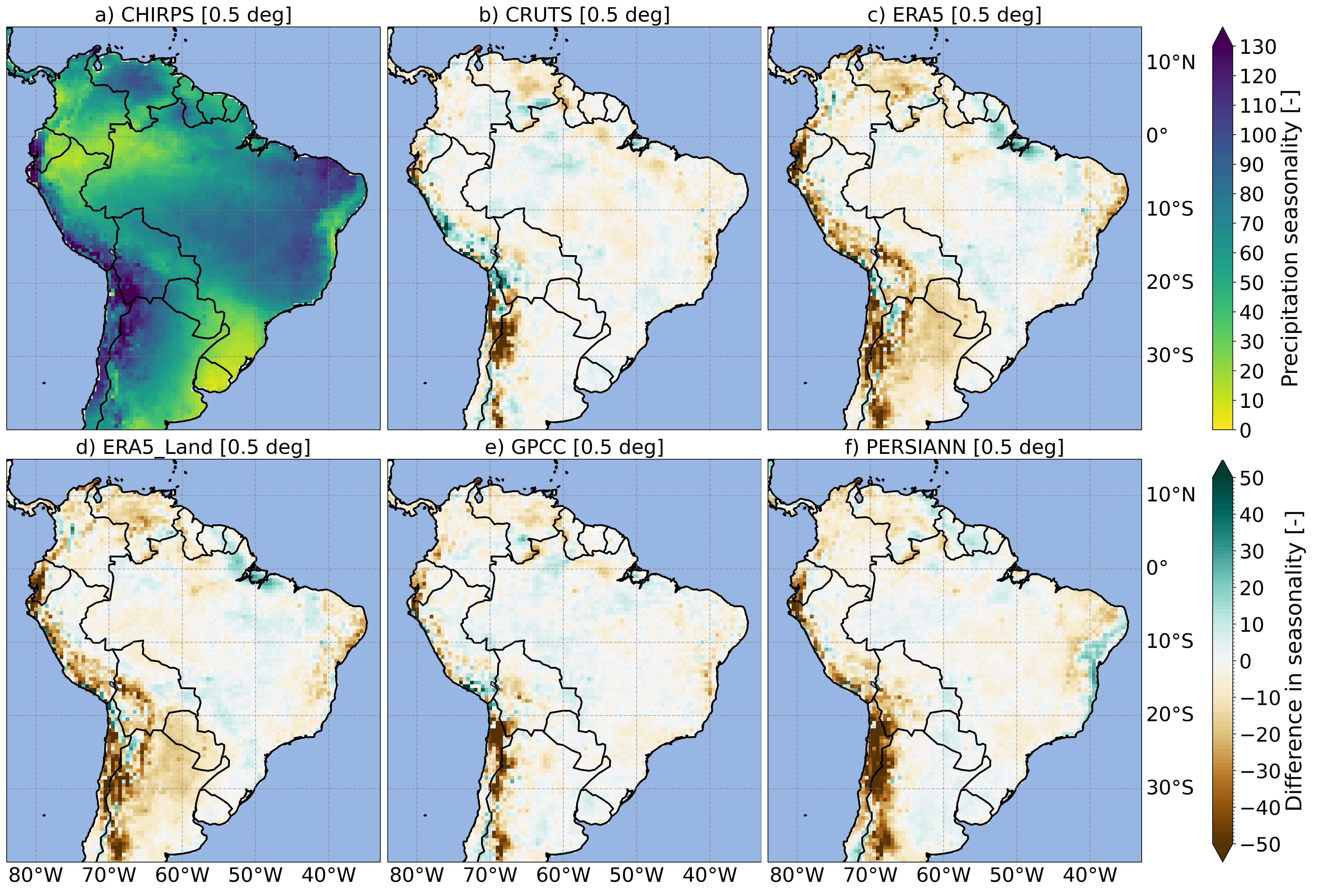
**Figure S9.** BCV15 - the seasonality of total precipitation [-] of CHIRPS (a) and, for all other GGPPs (b-f), the difference in seasonality [-] from CHIRPS is shown. All values are based on climatological monthly averages calculated over the base period (1991-2020). The top row shows CHIRPS (a), CRUTS (b), and ERA5 (c), while the bottom row displays ERA5-Land (d), GPCC (e), and PERSIANN (f). The maps are presented on a common 0.5 deg grid.


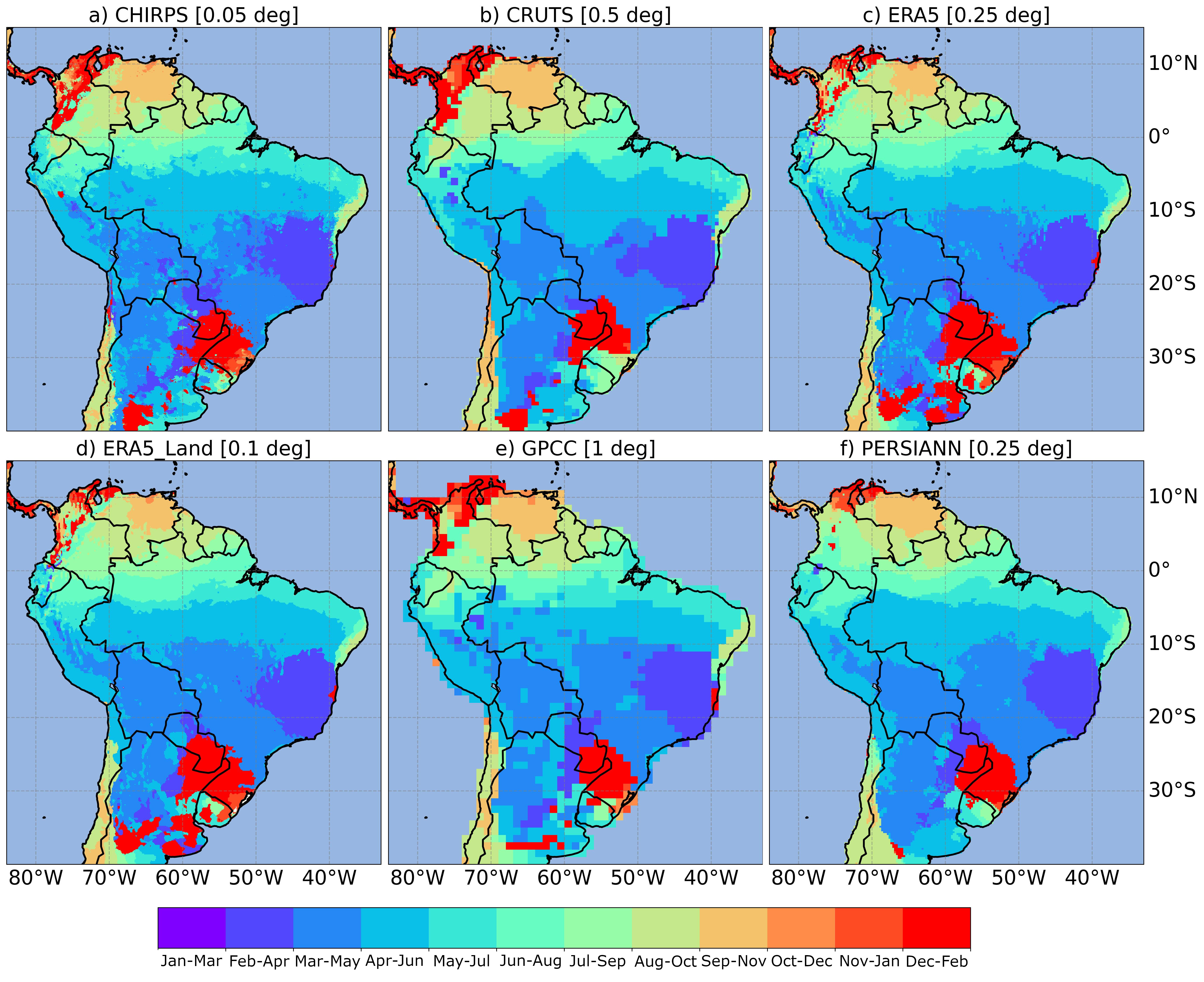


**Figure S10.** The climatological quarters of the wettest quarter derived from BCV16 (Figure 5) for all GGPPs. The top row shows CHIRPS (a), CRUTS (b), and ERA5 (c), while the bottom row displays ERA5-Land (d), GPCC (e), and PERSIANN (f). Data are shown at their native spatial resolution.


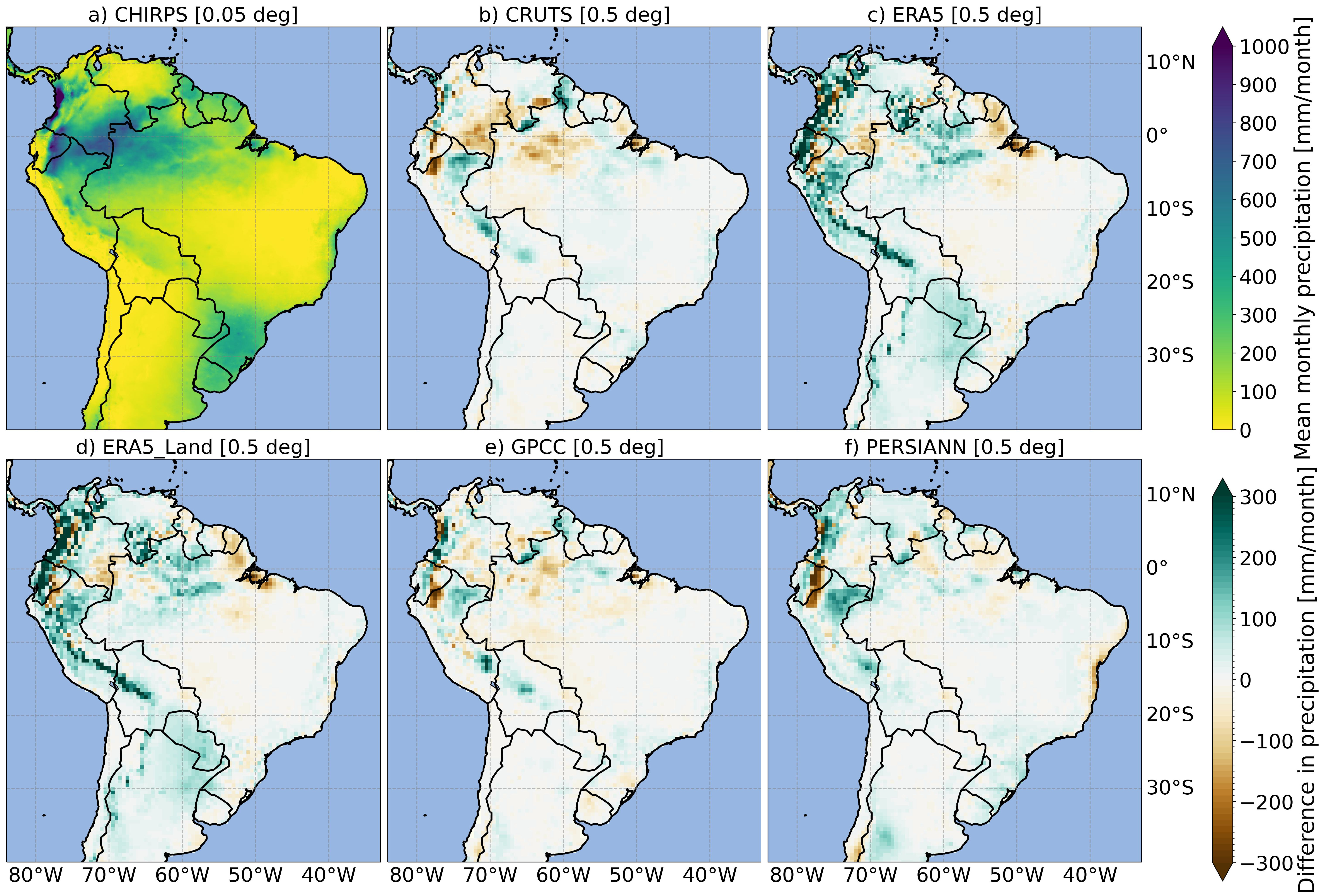
**Figure S11.** BCV17 – the mean monthly precipitation [mm/month] of CHIRPS (a) of the driest quarter and, for all other GGPP (b-f), the difference [mm] from CHIRPS is shown. All values are based on climatological monthly averages calculated over the base period (1991-2020). The top row shows CHIRPS (a), CRUTS (b), and ERA5 (c), while the bottom row displays ERA5-Land (d), GPCC (e), and PERSIANN (f). The maps are presented on a common 0.5 deg grid, except for CHIRPS, which is shown at its native spatial resolution.



**Figure S12.** Climatological annual cycles of monthly precipitation (mm/month) for Brazil (a) and BRA-VARAD1-P (b-g), calculated over the base period (1991-2020). The uppermost figure (a) displays the precipitation amounts for Brazil as a whole. The subsequent figures are organized as follows: the top row includes (b) Amazonas (1) and (c) Bahia (2); the middle row presents (d) Minas Gerais (9) and (e) Rio Grande do Sul (14); and the bottom row features (f) Rio de Janeiro (15) and (g) São Paulo (19). These climatological annual cycles are based on area-level exposure timeseries derived from GGPPs on a common 0.5 deg grid.



**Figure S13.** Climatological annual cycles of monthly precipitation (mm/month) for Colombia (a) and COL-VARAD1-P (b-g), calculated over the base period (1991-2020). The uppermost figure (a) displays the precipitation amounts for Colombia as a whole. The subsequent figures are organized as follows: the top row includes (b) Antioquia (1) and (c) Cundinamarca (2); the middle row presents (d) Huila (6) and (e) Norte de Santander (9); and the bottom row features (f) Santander (10) and (g) Tolima (11). These climatological annual cycles are based on area-level exposure timeseries derived from GGPPs on a common 0.5 deg grid. Please note the scale on the y-axis in comparison to Figure 7.


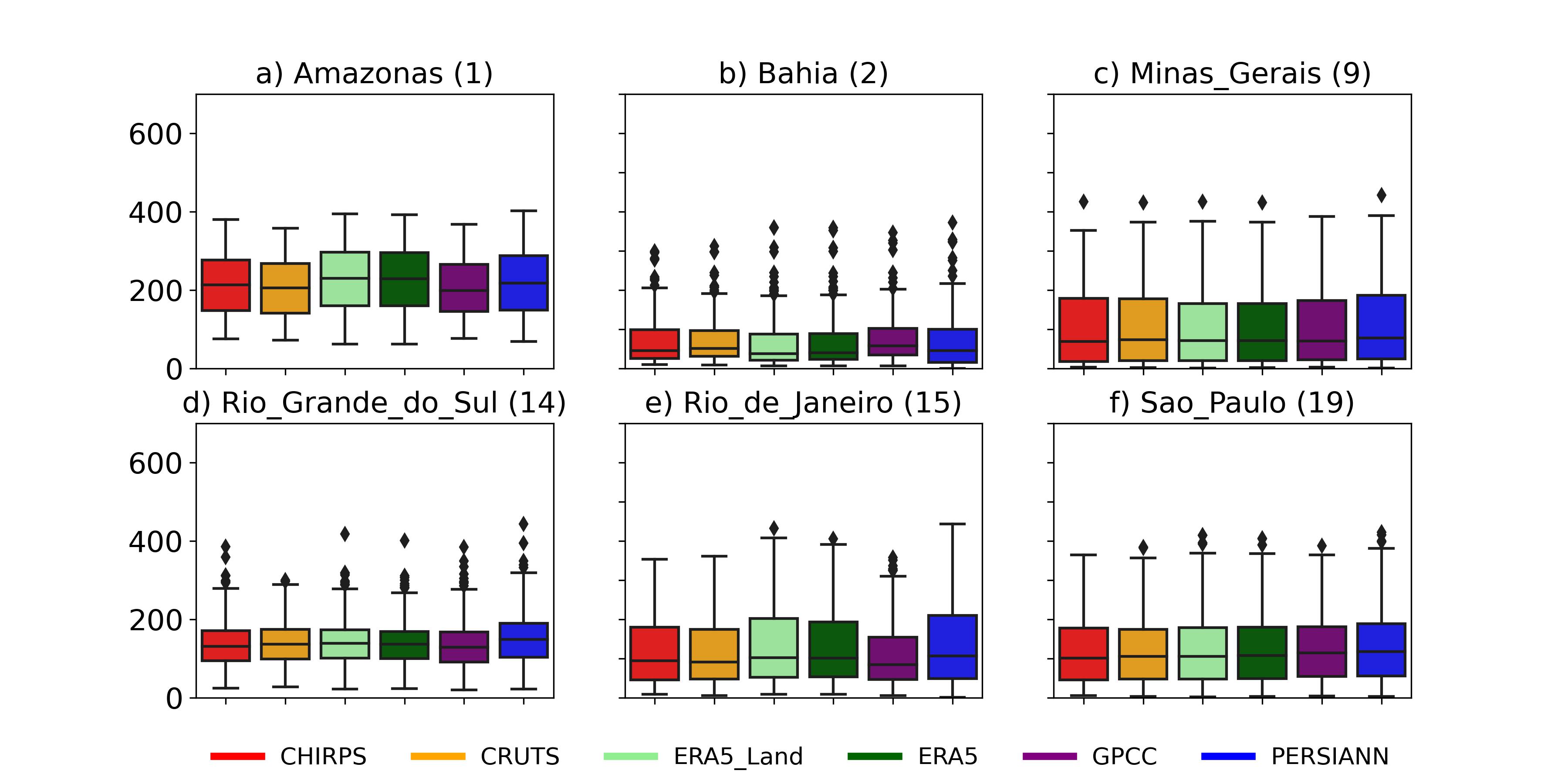
**Figure S14**. Boxplots comparing the precipitation distributions of monthly timeseries based on different GGPPs, averaged across each BRA-VARAD1-P, for the base period (1991-2020). The top row, from left to right, shows (a) Amazonas (1), (b) Bahia (2), (c) Minas Gerais (9), while the bottom row, from left to right, displays (d) Rio Grande do Sul (14), (e) Rio de Janeiro (15), and (f) São Paulo (19). The results are presented using area-level exposure estimates derived from GGPPs at their native spatial resolution.


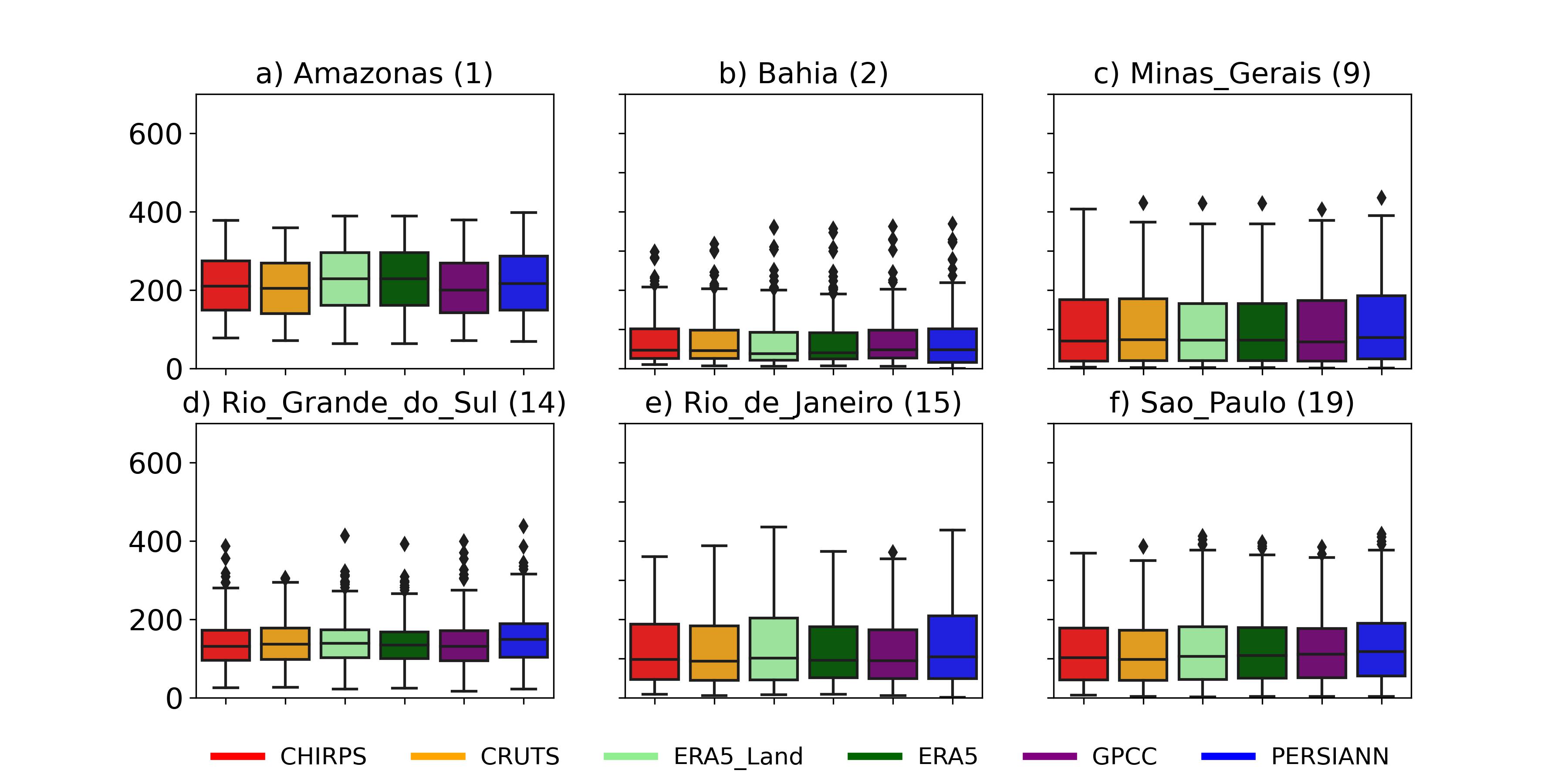
**Figure S15.** Boxplots comparing the precipitation distributions of monthly timeseries based on different GGPPs, averaged across each BRA-VARAD1-P, for the base period (1991-2020). The top row, from left to right, shows (a) Amazonas (1), (b) Bahia (2), (c) Minas Gerais (9), while the bottom row, from left to right, displays (d) Rio Grande do Sul (14), (e) Rio de Janeiro (15), and (f) São Paulo (19). The results are presented using area-level estimates derived from GGPPS on a common 0.5 deg grid.


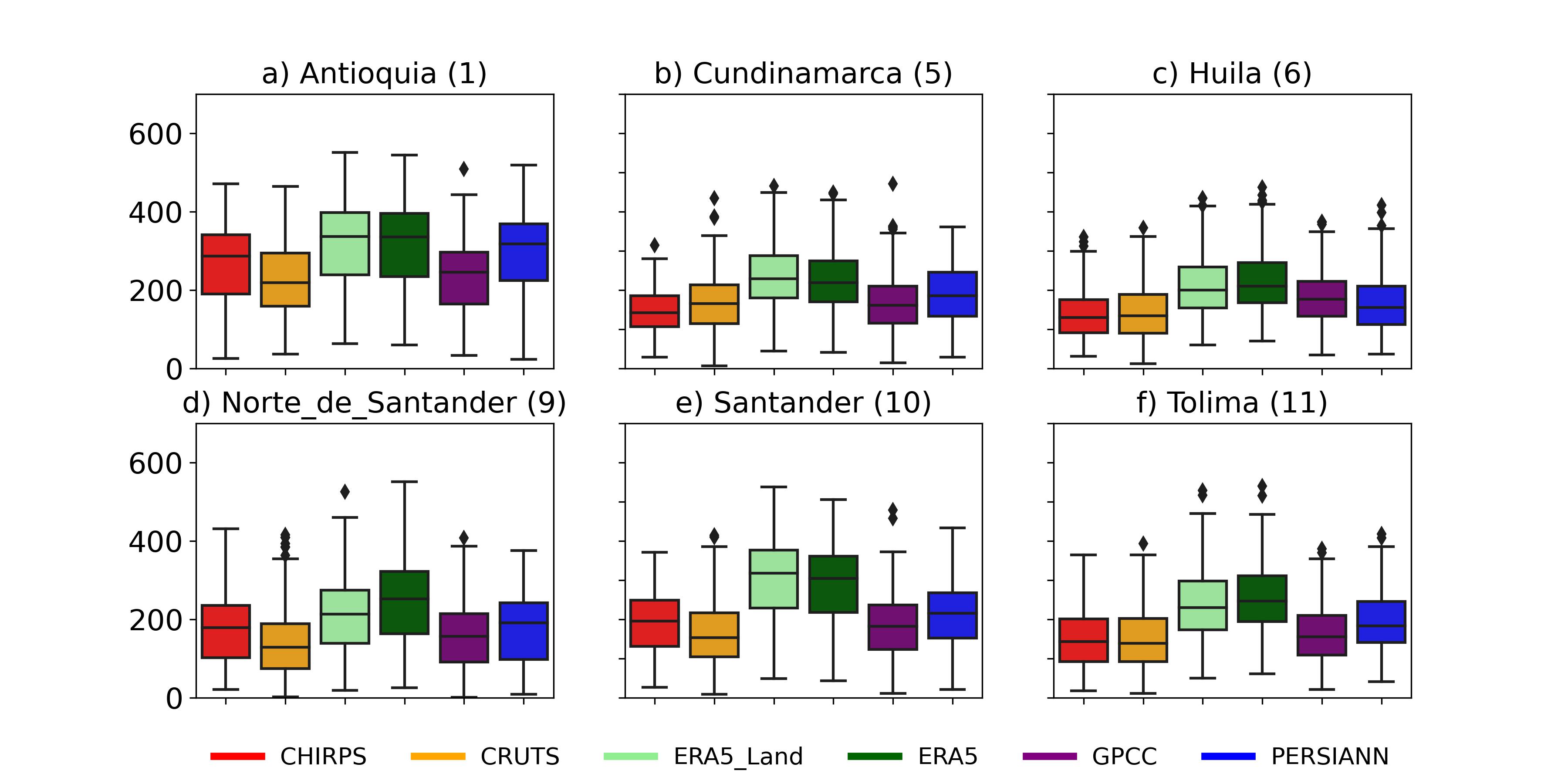
**Figure S16**. Boxplots comparing the precipitation distributions of monthly timeseries based on different GGPPs, averaged across each COL-VARAD1-P, for the base period (1991-2020). The top row, from left to right, shows (a) Antioquia (1), (b) Cundinamarca (5), and (c) Huila (6), while the bottom row, from left to right, displays (e) Norte de Santander (9), (f) Santander (10), and (g) Tolima (11). The results are presented using area-level exposure estimates derived from GGPPs at their native spatial resolution.


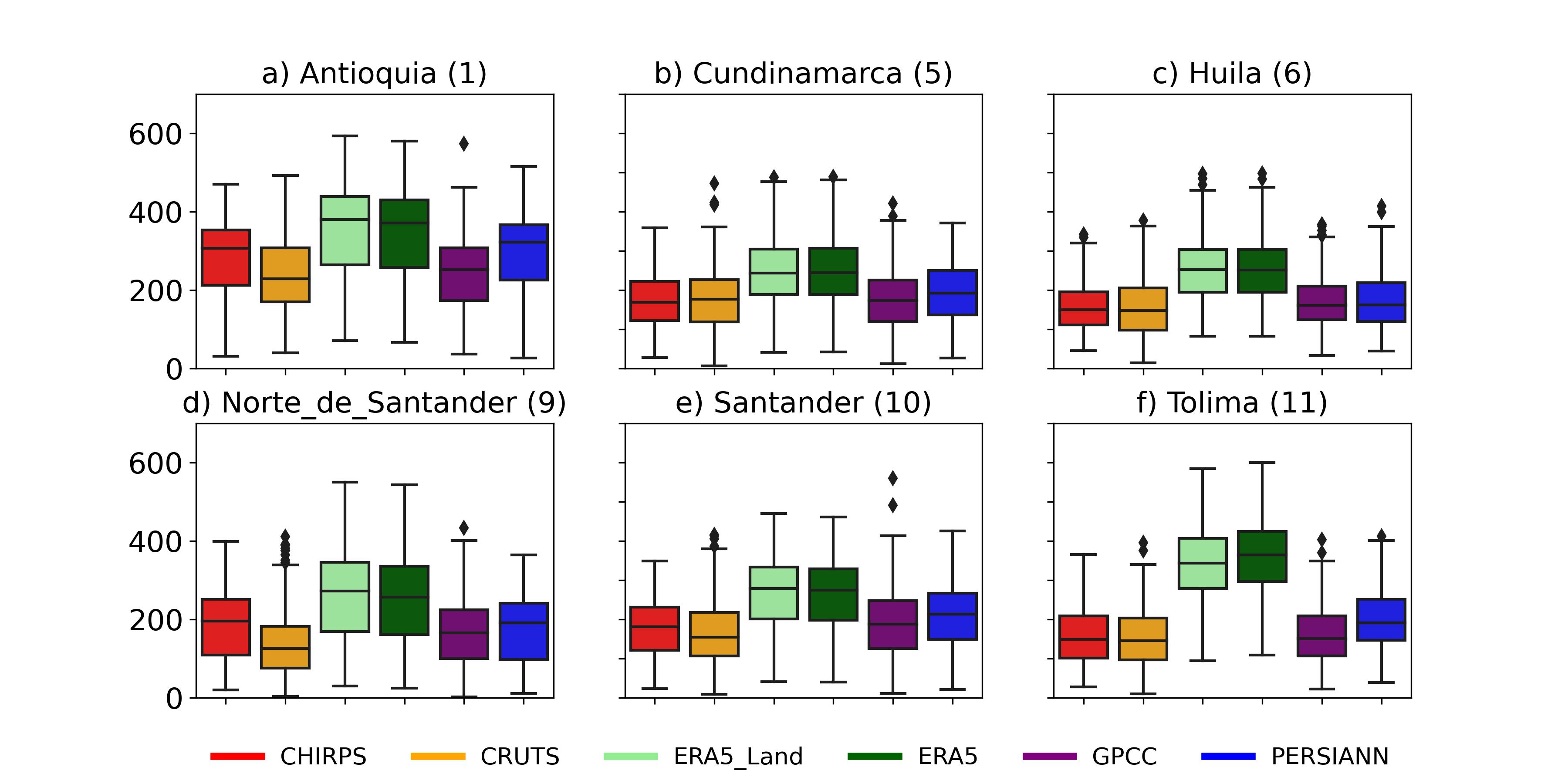
**Figure S17**. Boxplots comparing the precipitation distributions of monthly timeseries based on different GGPP, averaged across each COL-VARAD1-P, for the base period (1991-2020). The top row, from left to right, shows (a) Antioquia (1), (b) Cundinamarca (5), and (c) Huila (6), while the bottom row, from left to right, displays (e) Norte de Santander (9), (f) Santander (10), and (g) Tolima (11). The results are presented using area-level exposure estimates derived from GGPPs on a common 0.5 deg grid.


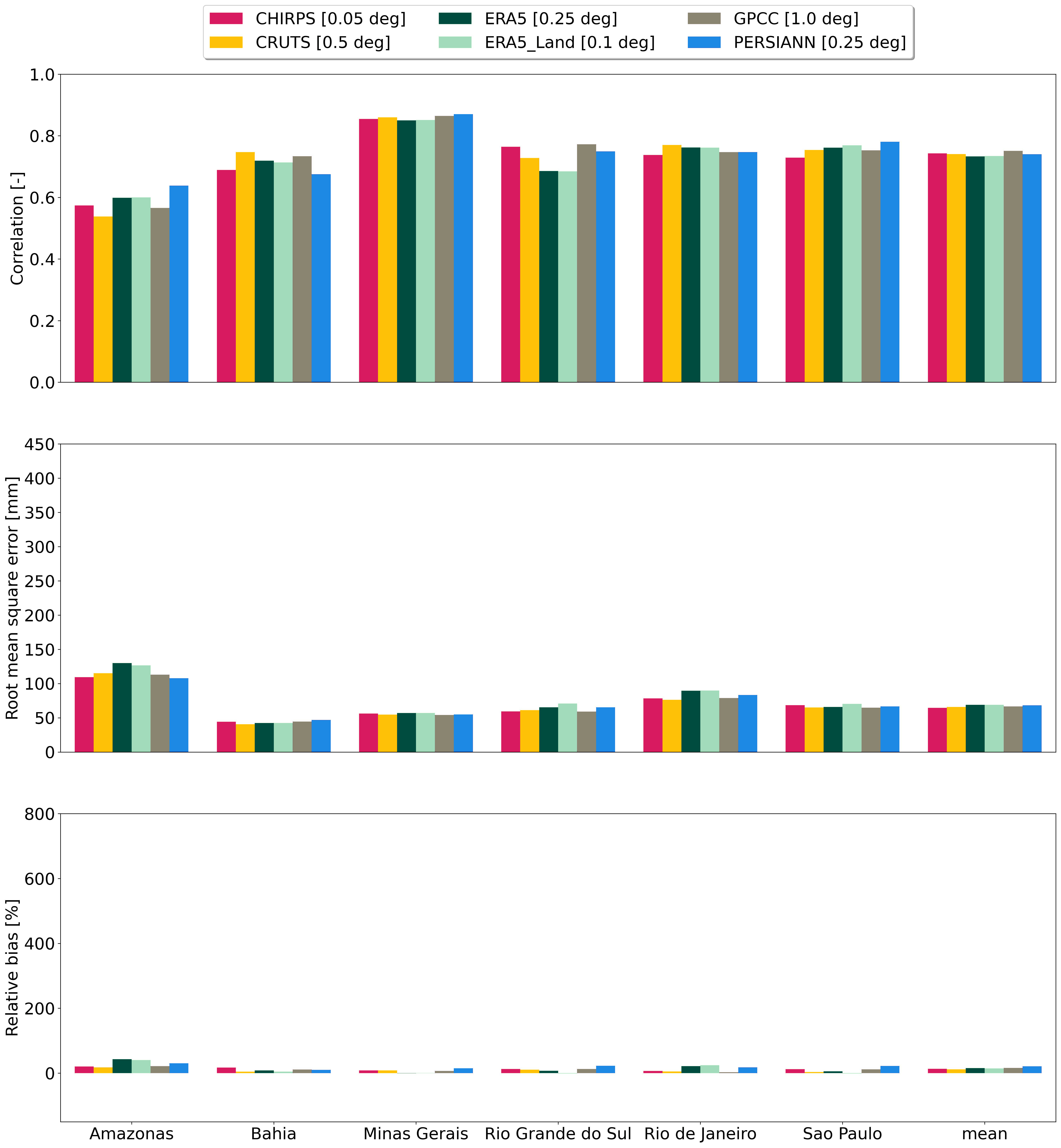
**Figure S18.** Statistical evaluation of monthly precipitation timeseries retrieved from GGPP pixels against ground observations: The evaluation presents the Pearson correlation (top), root mean square error (center), and relative bias (bottom) during the period 2011-2020 for each GGPP, averaged across each BRA-VARAD1-P. The BRA-VARAD1-P are depicted from left to right: Amazonas (1), Bahia (2), Minas Gerais (9), Rio Grande do Sul (14), Rio de Janeiro (15), and São Paulo (19). Mean values represent averages across all BRA-VARAD1. Please note the different number of weather stations evaluated across the different validation areas, as depicted in Tables S1 and S5. Only stations with significant correlations were included in the analysis. GGPPs in validation areas with no significant point-to-pixel correlations are not depicted. Results are shown for all GGPPs at their native spatial resolution.


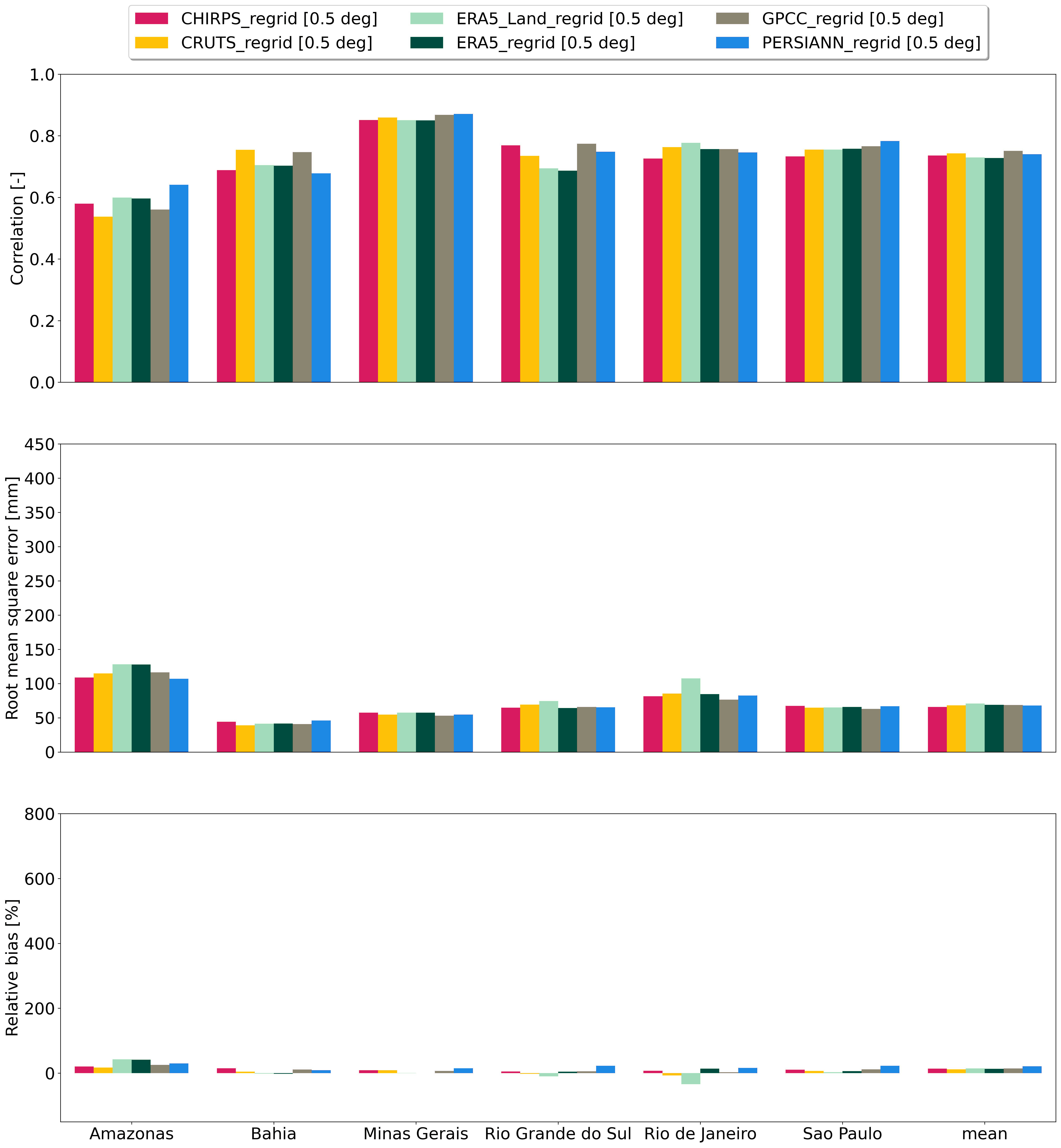


**Figure S19.** Statistical evaluation of monthly precipitation timeseries retrieved from GGPP pixels against ground observations: The evaluation presents the Pearson correlation (top), root mean square error (center), and relative bias (bottom) during the period 2011-2020 for each GGPP, averaged across each BRA-VARAD1-P. The BRA-VARAD1-P are depicted from left to right: Amazonas (1), Bahia (2), Minas Gerais (9), Rio Grande do Sul (14), Rio de Janeiro (15), and São Paulo (19). Mean values represent averages across all BRA-VARAD1. Please note the different number of weather stations evaluated across the different validation areas, as depicted in Tables S1 and S5. Only stations with significant correlations were included in the analysis. GGPPs in validation areas with no significant point-to-pixel correlations are not depicted. Results are shown for all GGPPs on a common 0.5 deg grid.


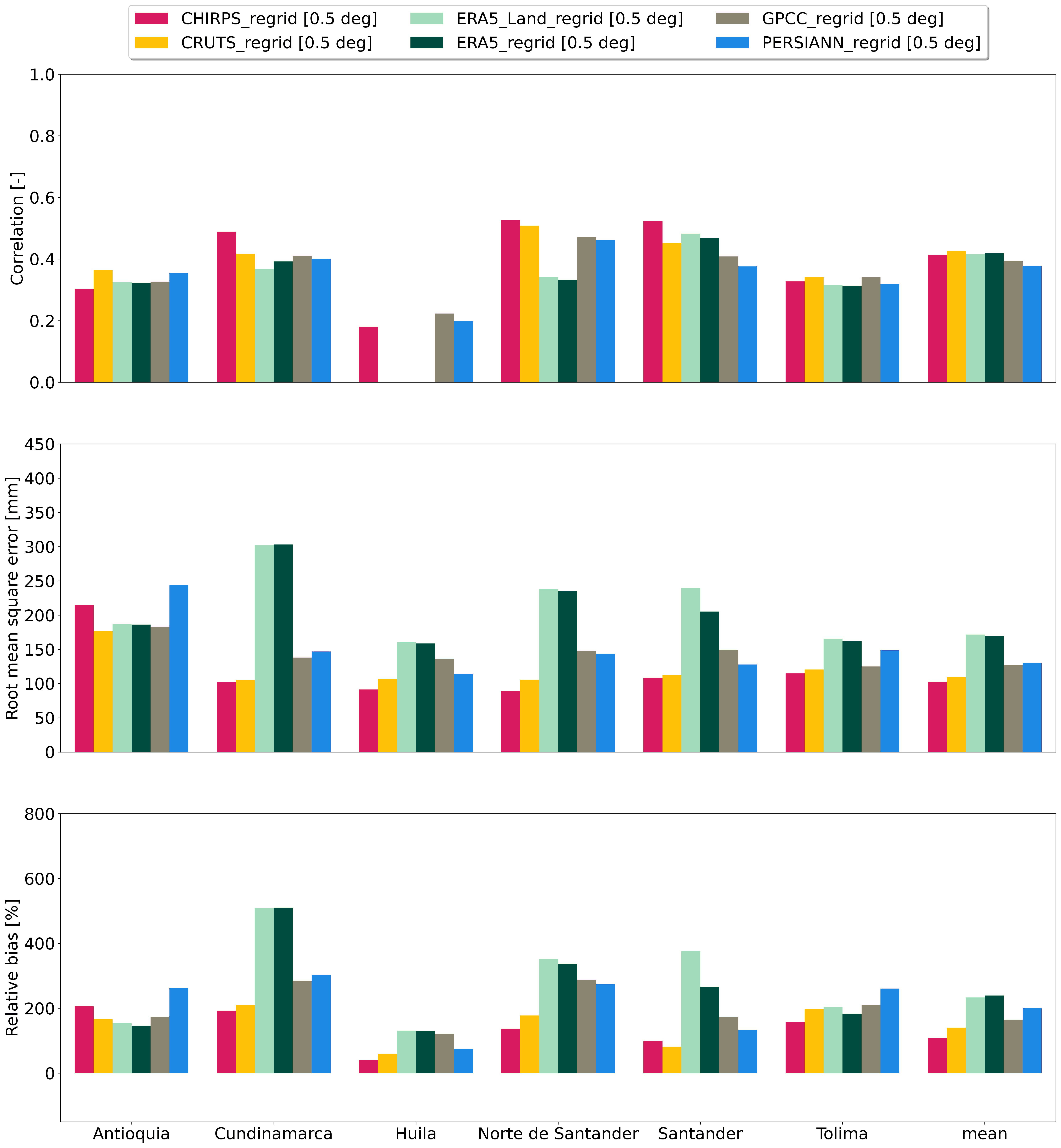


**Figure S20.** Statistical evaluation of monthly precipitation timeseries from GGPP pixels against ground observations: The evaluation presents the Pearson correlation (top), root mean square error (center), and relative bias (bottom) during the period 2011-2020 for each GGPP, averaged across each COL-VARAD1-P. The COL-VARAD1-P are depicted from left to right: Antioquia (1), Cundinamarca (2), Huila (6), Norte de Santander (9), Santander (10), and Tolima (11). Mean values represent averages across all COL-VARAD1. Please note that the number of weather stations evaluated varies across the different validation areas, as detailed in Tables S1 and S5. Only stations with significant correlations were included in the analysis. GGPPs in areas with no significant point-to-pixel correlations are not shown (e.g., for reanalysis in Huila (6)). Results are provided for all GGPPs on a common 0.5 deg grid.


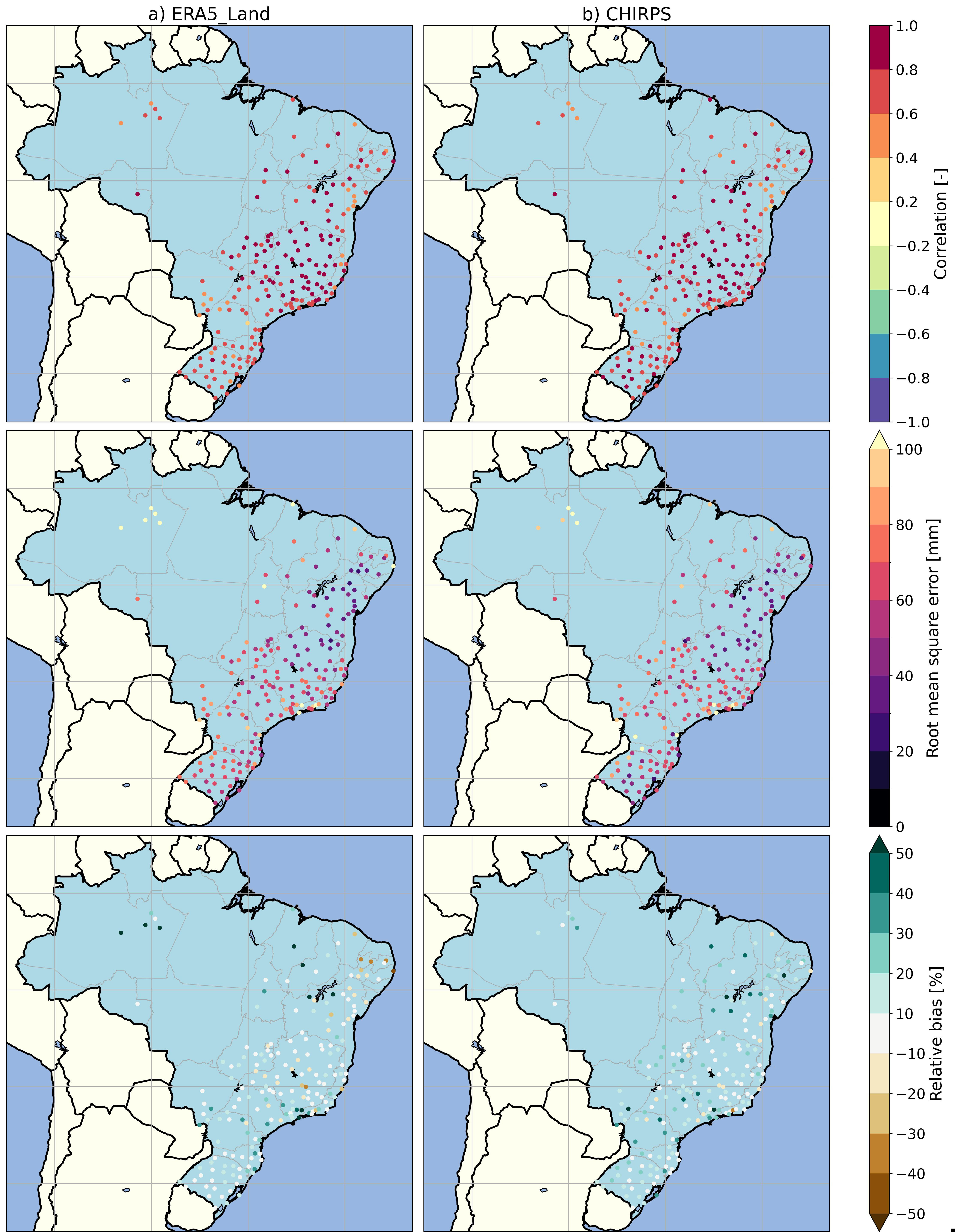


**Figure S21.** Comparison of Pearson correlation (top), root mean square error (center), and relative bias (bottom) between (a) ERA5-Land and (b) CHIRPS and ground-based observations at the corresponding station on a monthly time scale in Brazil.


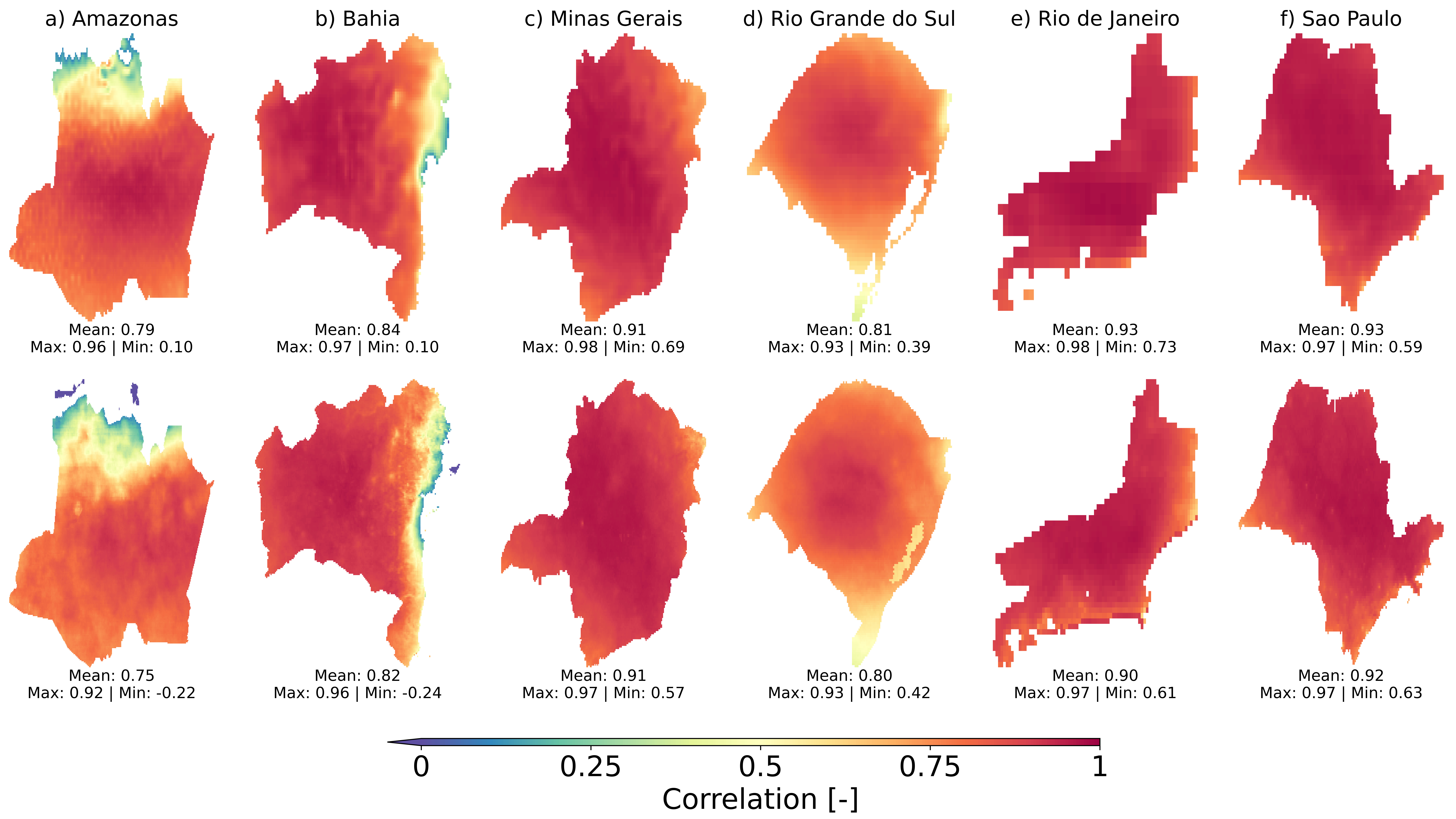


**Figure S22.** Pearson correlation derived by comparing the monthly precipitation in the base period observed at 0.1 deg resolution (ERA5-Land, top) and at 0.05 deg resolution (CHIRPS, bottom) against the aggregated area-level exposure estimates for each BRA-VARAD1-P. The top and bottom rows, from left to right, show (a) Amazonas (1), (b) Bahia (2), (c) Minas Gerais (9), (d) Rio Grande do Sul (14), (e) Rio de Janeiro (15), and (f) São Paulo (19). Pixels without significant correlations are shaded in grey. Within each map, text-based values indicate the minimum (min), mean, and maximum (max) correlation values for each BRA-VARAD1-P.


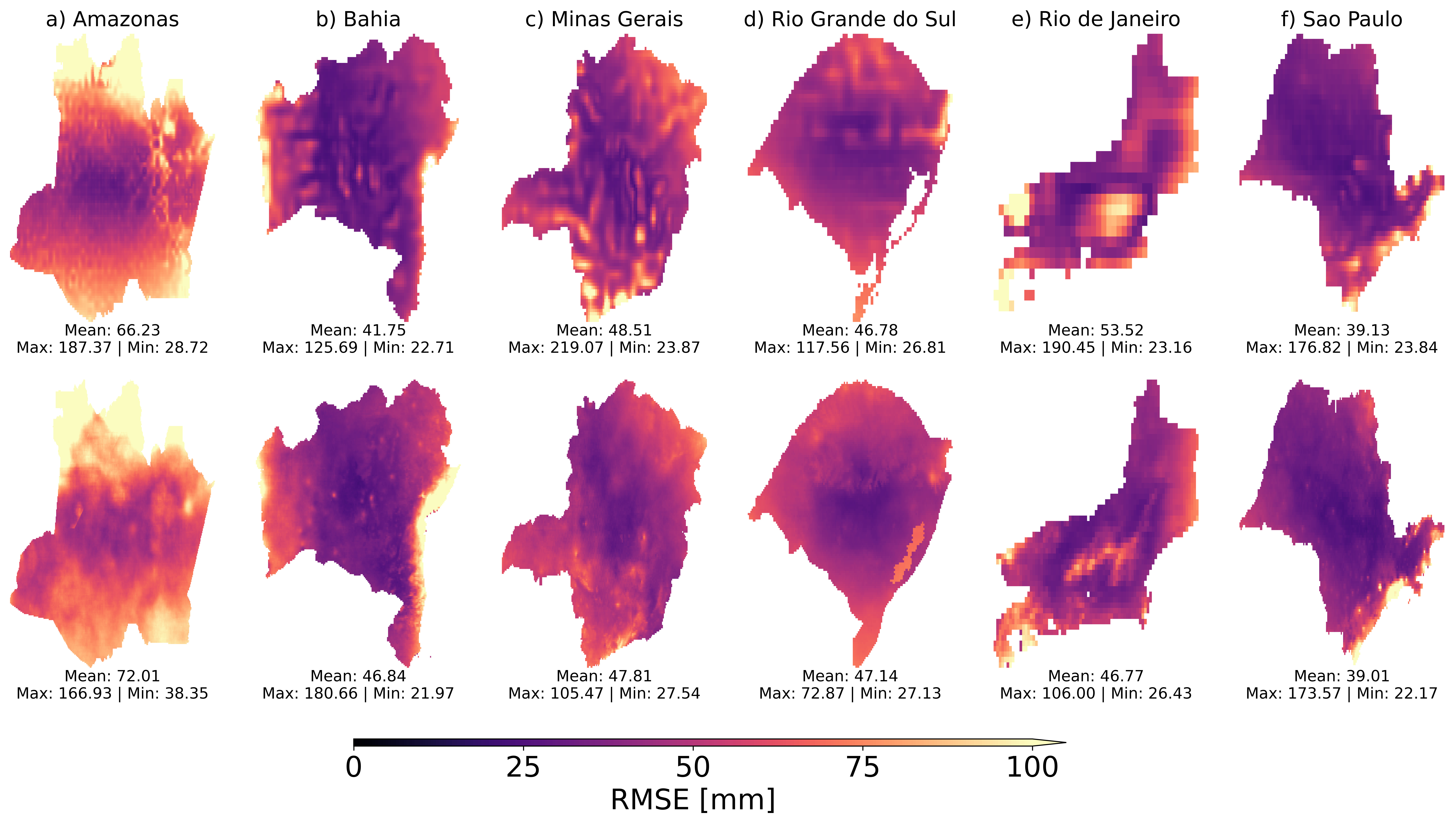


**Figure S23.** Root mean square error (RMSE) derived by comparing the monthly precipitation in the base period observed at 0.1 deg resolution (ERA5-Land, top) and at 0.05 deg resolution (CHIRPS, bottom) against the aggregated area-level exposure estimates for each BRA-VARAD1-P. The top and bottom rows, from left to right, show (a) Amazonas (1), (b) Bahia (2), (c) Minas Gerais (9), (d) Rio Grande do Sul (14), (e) Rio de Janeiro (15), and (f) São Paulo (19). Within each map, text-based values indicate the minimum (min), mean, and maximum (max) RMSE values for each BRA-VARAD1-P.


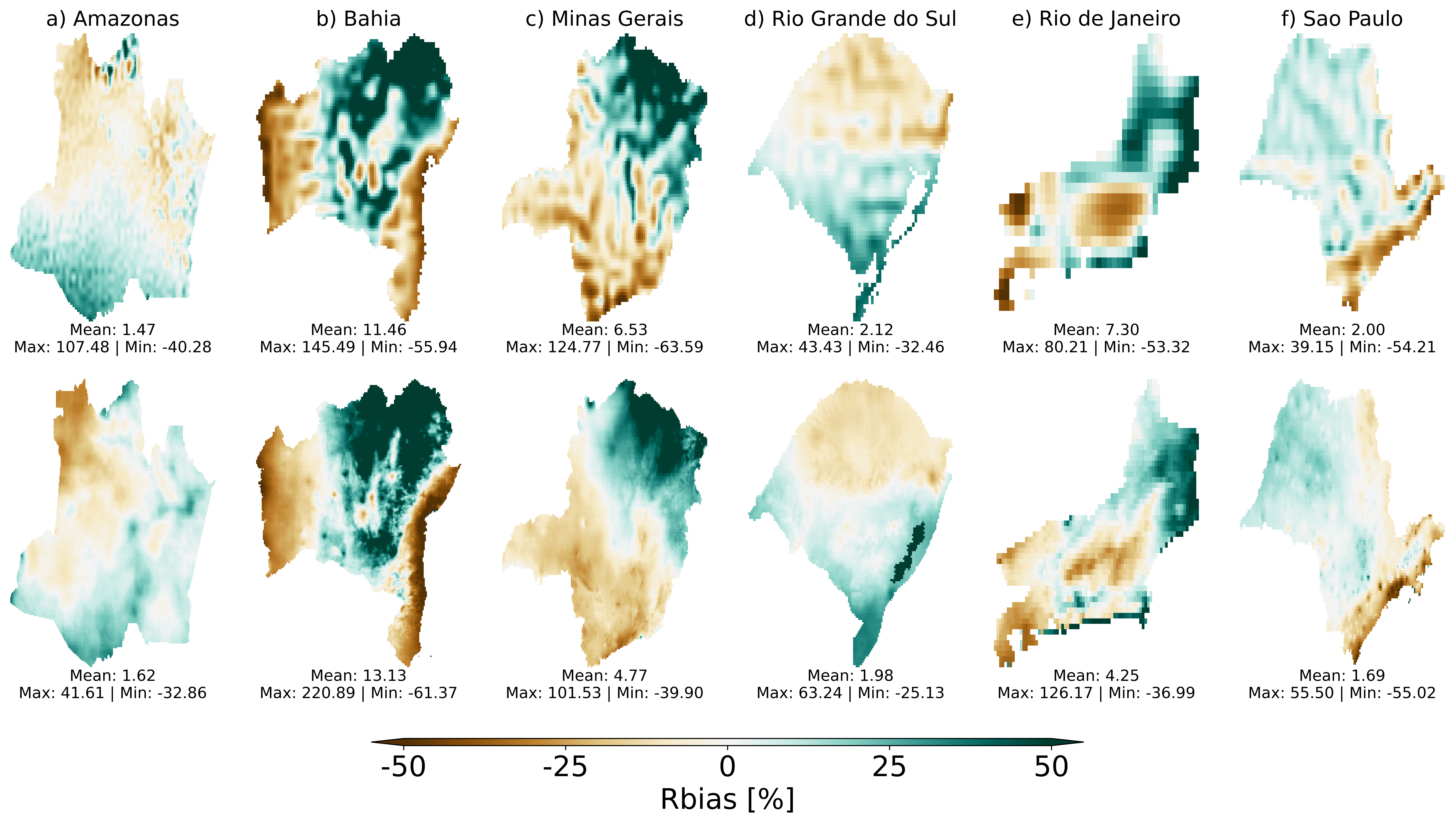


**Figure S24.** Relative bias (Rbias) derived by comparing the monthly precipitation in the base period observed at 0.1 deg resolution (ERA5-Land, top) and at 0.05 deg resolution (CHIRPS, bottom) against the aggregated area-level exposure estimates for each BRA-VARAD1-P. The top and bottom rows, from left to right, show (a) Amazonas (1), (b) Bahia (2), (c) Minas Gerais (9), (d) Rio Grande do Sul (14), (e) Rio de Janeiro (15), and (f) São Paulo (19). Within each map, text-based values indicate the minimum (min), mean, and maximum (max) Rbias values for each BRA-VARAD1-P.

**
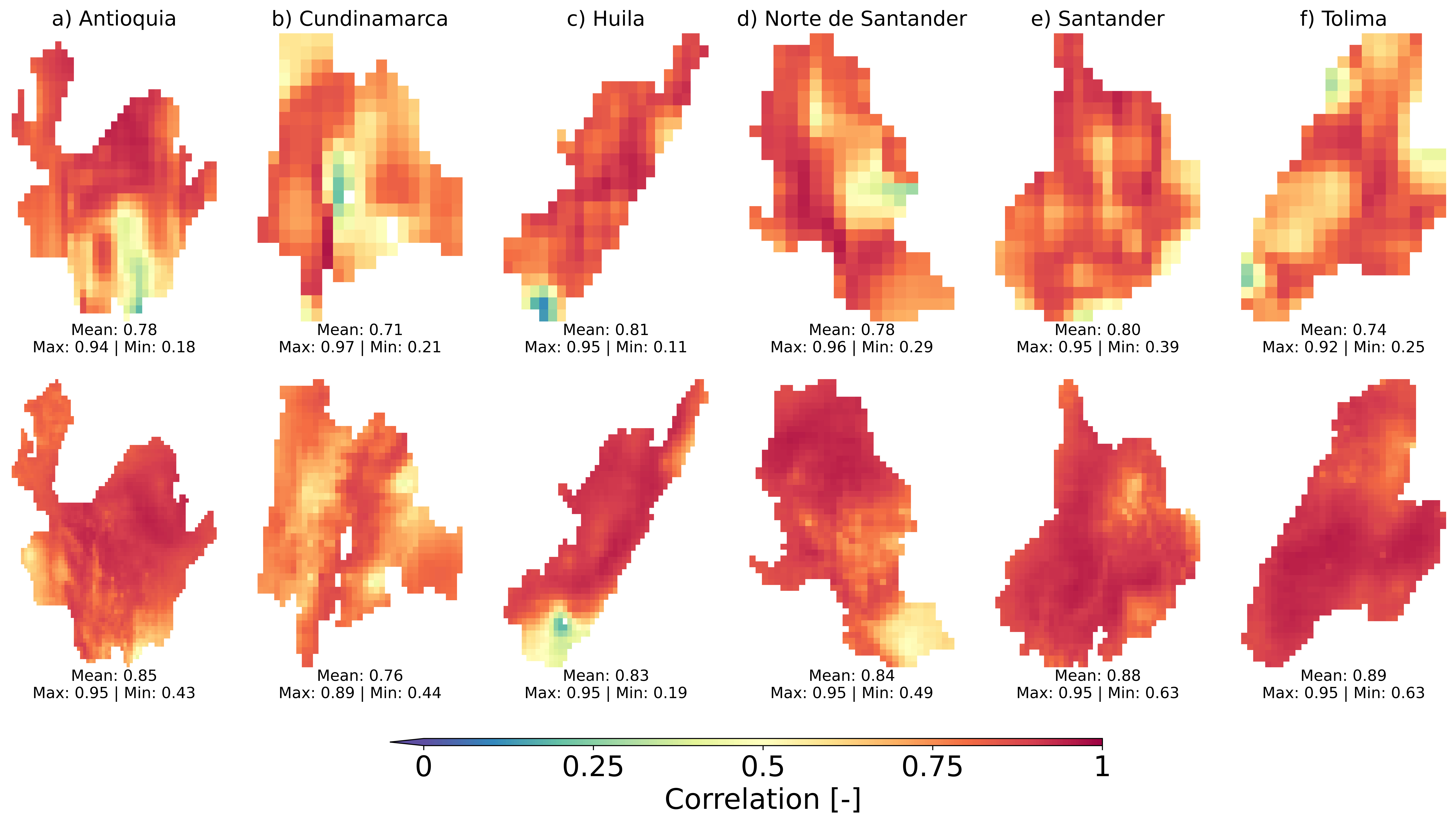
**

**Figure S25.** Pearson correlation derived by comparing the monthly precipitation in the base period observed at 0.1deg resolution (ERA5-Land, top) and at 0.05deg resolution (CHIRPS, bottom) against the aggregated area-level precipitation estimates for each COL-VARAD1-P area. The top and bottom rows, from left to right, show (a) Antioquia (1), (b) Cundinamarca (5), and (c) Huila (6), (d) Norte de Santander (9), (e) Santander (10), and (f) Tolima (11). Within each map, text-based values indicate the min, mean, and max correlation values for each COL-VARAD1-P.


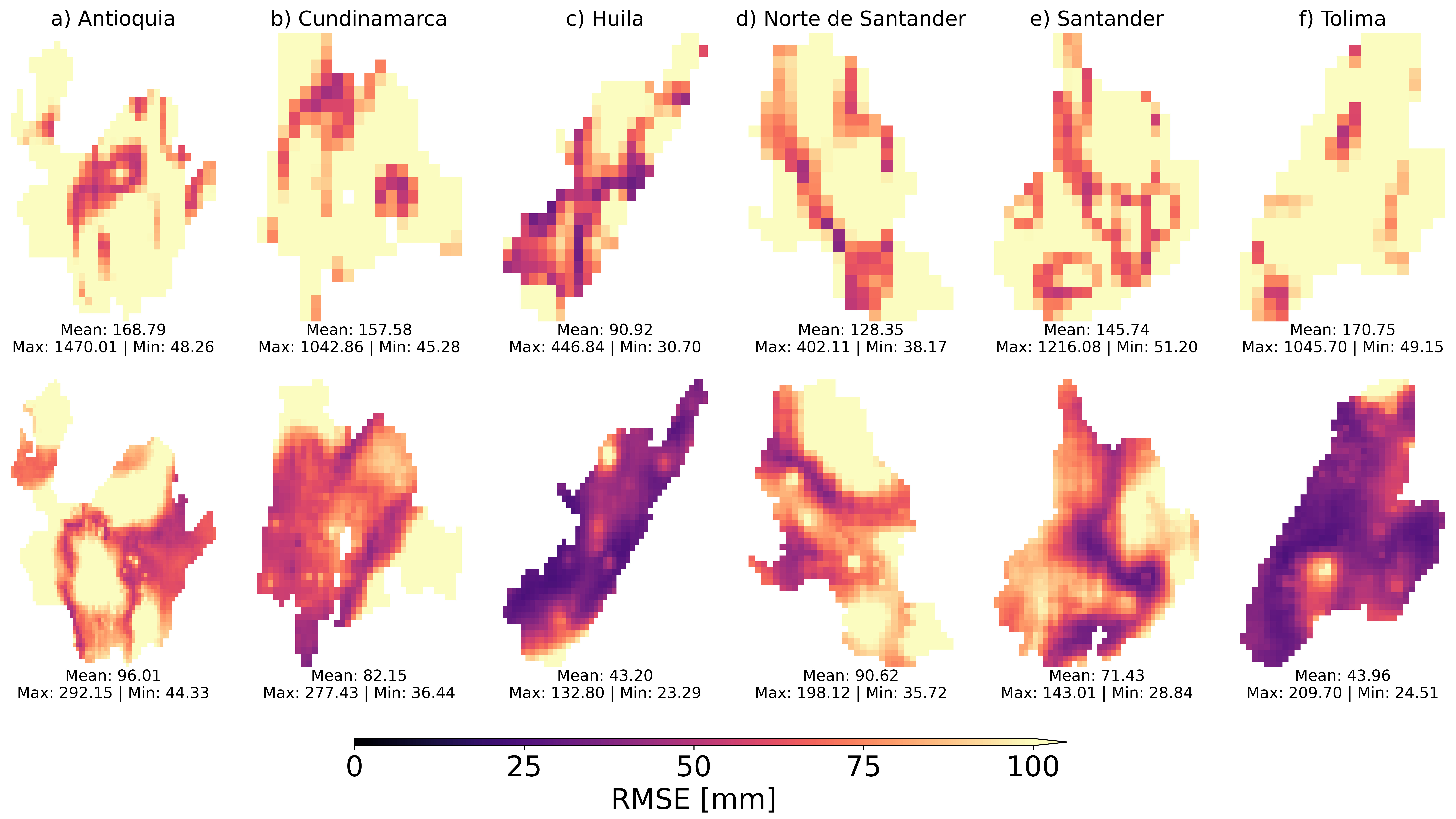


**Figure S26.** Root mean square error (RMSE) derived by comparing the monthly precipitation in the base period observed at 0.1 deg resolution (ERA5-Land, top) and at 0.05 deg resolution (CHIRPS, bottom) against the aggregated area-level exposure estimates for each COL-VARAD1-P. The top and bottom rows, from left to right, show (a) Antioquia (1), (b) Cundinamarca (5), and (c) Huila (6), (d) Norte de Santander (9), (e) Santander (10), and (f) Tolima (11). Within each map, text-based values indicate the minimum (min), mean, and maximum (max) RMSE values for each COL-VARAD1-P.

**Tables**

**Table S1.** Overview and descriptive statistics of the validation areas (VARAD1) for a) Brazil and b) Colombia, including the code and name of each area, number of weather stations, mean station elevation in meter (with minimum and maximum values in brackets) and total area size in km^2^. Areas marked with an asterisk (*) represent primary validation areas (VARAD1-P).

1. **Brazil**

| AdCod1 | Admin1 | Weather Stations | Station Elevation | Area |
| --- | --- | --- | --- | --- |
| 1* | Amazonas | 5 | 52.83 (19.9, 112.84) | 1570150 |
| 2* | Bahia | 22 | 467.94 (6.24, 1283.95) | 592969 |
| 3 | Ceará | 2 | 581.86 (298.19, 865.53) | 150137 |
| 4 | Distrito Federal | 2 | 1095.66 (1030.36, 1160.96) | 6217.88 |
| 5 | Espírito Santo | 6 | 73.47 (9.0, 156.02) | 51875.9 |
| 6 | Goiás | 12 | 782.88 (491.17, 1211.08) | 368516 |
| 7 | Maranhão | 3 | 157.91 (35.86, 283.69) | 332604 |
| 8 | Mato Grosso do Sul | 7 | 405.18 (324.31, 528.47) | 406035 |
| 9* | Minas Gerais | 43 | 747.79 (189.11, 1544.89) | 652280 |
| 10 | Paraná | 9 | 568.37 (50.31, 1016.0) | 241045 |
| 11 | Paraíba | 4 | 405.08 (237.04, 573.45) | 57345.9 |
| 12 | Pernambuco | 6 | 407.08 (11.3, 827.78) | 100161 |
| 13 | Piauí | 3 | 360.97 (312.07, 398.83) | 256228 |
| 14* | Rio Grande do Sul | 28 | 349.38 (3.82, 1228.59) | 373701 |
| 15* | Rio de Janeiro | 13 | 208.76 (3.0, 1070.0) | 50989.9 |
| 16 | Rondônia | 1 | 183.53 (183.53, 183.53) | 246349 |
| 17 | Santa Catarina | 12 | 455.68 (2.0, 1400.06) | 120875 |
| 18 | Sergipe | 1 | 204.8 (204.8, 204.8) | 22700.6 |
| 19* | São Paulo | 17 | 651.84 (2.7, 1662.95) | 289707 |
| 20 | Tocantins | 3 | 243.57 (189.71, 274.0) | 286587 |

1. **Colombia**

| AdCod1 | Admin1 | Weather Stations | Station Elevation | Area |
| --- | --- | --- | --- | --- |
| 1* | Antioquia | 5 | 887.6 (15, 2125) | 63857.4 |
| 2 | Bogotá D.C. | 3 | 2942.0 (2556, 3624) | 1644.06 |
| 3 | Boyacá | 2 | 2126.0 (742, 3510) | 23319 |
| 4 | Cauca | 1 | 1800.0 (1800, 1800) | 30573.5 |
| 5* | Cundinamarca | 14 | 1818.79 (170, 3257) | 22518.1 |
| 6* | Huila | 4 | 1867.5 (1550, 2101) | 18741.9 |
| 7 | La Guajira | 2 | 94.5 (45, 144) | 21292.8 |
| 8 | Magdalena | 1 | 7.0 (7, 7) | 23507.3 |
| 9* | Norte de Santander | 4 | 772.75 (240, 1320) | 22380 |
| 10* | Santander | 5 | 1767.6 (92, 3600) | 31009.9 |
| 11* | Tolima | 8 | 1339.75 (323, 3394) | 24094 |

**Table S2.** Definitions and calculations of a) climate change indices (ETCCDI) and b) bioclimatic variables (BCVs). Indices and variables were calculated based on GGPPs at their native spatial resolution as well as on a common 0.5 deg grid in order to consider uncertainties arising from different datasets. ETCCDI calculations were all based on timeseries of daily precipitation amounts. Hence, datasets providing daily resolution were selected to generate ETCCDI (CRUTS excluded). The input data to calculate BCVs is normally monthly, averaged across a prolonged time period (all GGPPs included). Hence, GGPP information with respect to the base period, i.e., a 30-year time slice (1991-2020) was used.

1. **ETCCDI**

| Index | Label | Definition and Calculation | Unit |
| --- | --- | --- | --- |
| Simple daily intensity | SDII | The value presents the mean precipitation amount at wet days (Precipitation threshold >= 1 mm) calculated per base period. | mm |
| Very heavy precipitation days | R20mm | The index is defined to get the number of days with precipitation exceeding 20 mm for a timeseries of daily precipitation amounts. | –  (number of days) |
| Consecutive  wet days | CWD | The index provides two output variables: A) the largest number of consecutive wet days of a timeseries of daily precipitation amounts where values are above a precipitation threshold of 1mm over the entire timeseries.; B) the number of wet periods of more than 5 days in a given time period. Here, only B) for the base period is considered. | –  (A: number of days  or  B: number of wet periods) |
| Consecutive  dry days | CDD | The index provides two output variables: A) the largest number of consecutive dry days of a timeseries of daily precipitation amounts where values are less than a precipitation threshold of 1mm; B) the number of dry periods of more than 5 days in a given time period . Here, only B) for the base period is considered. | –  (A: number of days  or  B: number of dry periods) |

1. **BCVs**

| Variable | Label | Definition and Calculation | Unit |
| --- | --- | --- | --- |
| Total (annual) precipitation amount | BCV12 | The value represents the average accumulated precipitation amount over one year. | mm |
| Precipitation amount of the wettest month | BCV13 | The variable defines the precipitation of the wettest month. | mm |
| Precipitation amount of the driest month | BCV14 | The variable defines the precipitation of the driest month. | mm |
| Precipitation seasonality | BCV15 | The value is based on the coefficient of variation, being the standard deviation of the monthly precipitation estimates. Seasonality is expressed as a fraction of the mean of those estimates (i.e., the annual mean). | –  (fraction) |
| Mean monthly precipitation amount of the wettest quarter | BCV16 | The variable defines the precipitation of the wettest quarter of the year. | mm |
| Mean monthly precipitation amount of the driest quarter | BCV17 | The variable defines the precipitation of the driest quarter of the year. | mm |

**Table S3.** Descriptive statistics of monthly area-level precipitation timeseries (in mm) in the base period (1991-2020) in a) Brazil and b) Colombia, based on spatial aggregation of each GGPP at its native spatial resolution.

1. **Brazil**

| Metric | CHIRPS | CRUTS | ERA5 | ERA5-Land | GPCC | PERSIANN |
| --- | --- | --- | --- | --- | --- | --- |
| Mean | 147.5 | 149.62 | 149.06 | 149.13 | 145.27 | 151.79 |
| Min | 33.84 | 41.25 | 33.85 | 35.38 | 36.51 | 31.87 |
| 25% | 76.88 | 83.77 | 75.88 | 77 | 82.52 | 80.76 |
| 50% | 146.71 | 149.3 | 153.08 | 152.9 | 146 | 152.74 |
| 75% | 209.35 | 209.5 | 210.78 | 210.66 | 200.93 | 213.94 |
| 99% | 275.15 | 270.33 | 278.89 | 276.27 | 260.89 | 288.56 |
| Max | 281.68 | 276.26 | 293.42 | 290.55 | 269.18 | 299.22 |
| Std | 68.84 | 65.55 | 70.95 | 69.84 | 62.77 | 71.34 |

1. **Colombia**

| Metric | CHIRPS | CRUTS | ERA5 | ERA5-Land | GPCC | PERSIANN |
| --- | --- | --- | --- | --- | --- | --- |
| Mean | 237.28 | 213.05 | 282.28 | 279.88 | 225.56 | 240.53 |
| Min | 53.54 | 63.82 | 63.95 | 63.1 | 64.83 | 62.02 |
| 25% | 187.35 | 166.93 | 234.79 | 232.83 | 181.15 | 200.9 |
| 50% | 249.73 | 214.97 | 293.54 | 290.78 | 231.79 | 247.84 |
| 75% | 290.85 | 253.95 | 334.16 | 329.46 | 270.94 | 290.12 |
| 99% | 368.32 | 355.85 | 431.18 | 430.32 | 370.85 | 380.54 |
| Max | 393.14 | 379.01 | 464.25 | 464.66 | 396.2 | 419.32 |
| Std | 73.75 | 68.14 | 77.86 | 78.27 | 65.08 | 71.52 |

**Table S4.** Descriptive statistics of ETCCDI and BCVs calculated from country-specific aggregated daily precipitation timeseries and climatological monthly means for Brazil and Colombia, respectively. The timeseries were obtained by spatially aggregating the GGPPs at their native spatial resolution. Units for the indices and variables are provided in Table S2. Climatological months (or quarters) for the wettest and driest periods are indicated in brackets for the respective BCVs.

1. **Brazil**

| Metric | CHIRPS | CRUTS | ERA5 | ERA5-Land | GPCC | PERSIANN |
| --- | --- | --- | --- | --- | --- | --- |
| SDII | 4.99 | - | 5.02 | 5.03 | 4.85 | 5.16 |
| R20mm | 0 | 0 | 0 | 0 | 0 | 0 |
| CDD | 12 | - | 8 | 13 | 0 | 15 |
| CWD | 132 | - | 106 | 113 | 101 | 124 |
| BCV12 | 1770.05 | 1795.44 | 1789.5 | 1788.77 | 1743.24 | 1824.08 |
| BCV13 | 243.48  (Mar) | 239.23  (Mar) | 238.23  (Mar) | 238.87  (Mar) | 229.64  (Mar) | 246.69  (Mar) |
| BCV14 | 53.3  (Aug) | 57.74  (Aug) | 52.29  (Aug) | 51.13  (Aug) | 56.55  (Aug) | 54.46  (Aug) |
| BCV15 | 47.13 | 44.44 | 47.11 | 47.87 | 43.62 | 47 |
| BCV16 | 698.09  (Jan-Mar) | 694.92  (Jan-Mar) | 696.51  (Jan-Mar) | 700  (Jan-Mar) | 666.98  (Jan-Mar) | 716.72  (Jan-  Mar) |
| BCV17 | 189.78  (Jul-Aug) | 205.71  (Jul-Aug) | 188.05  (Jul-Aug) | 184.55  (Jul-Aug) | 201.94  (Jul-Aug) | 195.63  (Jul-Aug) |

1. **Colombia**

| Metric | CHIRPS | CRUTS | ERA5 | ERA5-Land | GPCC | PERSIANN |
| --- | --- | --- | --- | --- | --- | --- |
| SDII | 8.06 | - | 9.29 | 9.22 | 7.55 | 8.14 |
| R20mm | 183 | - | 112 | 112 | 212 | 162 |
| CDD | 12 | - | 0 | 0 | 2 | 13 |
| CWD | 133 | - | 15 | 16 | 130 | 110 |
| BCV12 | 2847.42 | 2556.64 | 3387.4 | 3358.54 | 2706.77 | 2883.08 |
| BCV13 | 340.84  (May) | 297.07  (May) | 393.99  (May) | 394.06  (May) | 322.75  (May) | 346.4  (May) |
| BCV14 | 118.94  (Jan) | 108.49  (Jan) | 160.99  (Jan) | 157.18  (Jan) | 129.15  (Jan) | 125.55  (Jan) |
| BCV15 | 29.67 | 28.17 | 25.73 | 26.07 | 26.59 | 27.78 |
| BCV16 | 935.02  (May-Jul) | 837.4  (May-Jul) | 1091.73  (Apr-Jun) | 1092.59  (Apr-Jun) | 879.76  (Apr-Jun) | 955.85  (Apr-Jun) |
| BCV17 | 413.45  (Dec-Feb) | 382.53  (Dec-Feb) | 545.46  (Dec-Feb) | 537.18  (Dec-Feb) | 433.34  (Dec-Feb) | 443.42  (Dec-Feb) |

**Table S5.** Distribution of precipitation (in mm) based on monthly timeseries for each weather station in a) BRA-VARAD1 and b) COL-VARAD1 during the evaluation period (2011-2020). The stations’ average value represents the mean across all stations within a validation area. Additionally, the table includes latitude [deg], longitude [deg], and elevation [m] for each selected weather station. Station names were prefixed with the first three letters from their respective validation area and each station was numbered. Stations marked with an asterisk (*) were excluded from dataset evaluation because not all GGPPs at their native spatial resolution provided pixel-based information for these sites.

1. **Brazil (BRA-VARAD1)**

| Station | Lat | Lon | Elev | Mean | Min | 25% | 50% | 75% | Max | Std |
| --- | --- | --- | --- | --- | --- | --- | --- | --- | --- | --- |
| **Individual Stations** | | | | | | | | | | |
| Ama1 | -2.06 | -60.03 | 60.99 | 177.76 | 0 | 94.5 | 168.2 | 260.8 | 474.4 | 123.41 |
| Ama2 | -2.63 | -59.6 | 112.84 | 178.17 | 0 | 81.45 | 156.6 | 266.5 | 504 | 124.13 |
| Ama3 | -3.58 | -59.13 | 19.9 | 136.54 | 0 | 53.5 | 116.6 | 215.7 | 391.2 | 106.03 |
| Ama4 | -3.29 | -60.63 | 36.57 | 165.63 | 0 | 50.9 | 143.4 | 246.2 | 551.8 | 128.94 |
| Ama5 | -4.1 | -63.15 | 33.84 | 155.1 | 0 | 62.55 | 145.1 | 233.25 | 570.6 | 116.03 |
| Bah6* | -13.01 | -38.51 | 47.56 | 127.8 | 0 | 41.05 | 94.4 | 190.8 | 527 | 106.61 |
| Bah7 | -15.24 | -40.23 | 271.48 | 59.53 | 0 | 16.5 | 44.5 | 77.25 | 279.6 | 59.33 |
| Bah8 | -10.46 | -41.21 | 646.24 | 28.08 | 0 | 2.6 | 10 | 40.6 | 320 | 47.34 |
| Bah9 | -10.54 | -39 | 431.96 | 44.88 | 0 | 6.35 | 25.5 | 72.9 | 210.2 | 47.3 |
| Bah10 | -11.66 | -39.02 | 337.79 | 44.12 | 0 | 10.9 | 37.3 | 67.05 | 236.4 | 40.69 |
| Bah11 | -12.19 | -43.21 | 425.12 | 43.81 | 0 | 0 | 7 | 57.65 | 363.2 | 69.49 |
| Bah12* | -13.91 | -38.97 | 6.24 | 167.54 | 0 | 91.55 | 145.3 | 236.5 | 463.2 | 107.69 |
| Bah13 | -10.98 | -39.62 | 310.11 | 38.92 | 0 | 7 | 27 | 50.65 | 245.4 | 44.95 |
| Bah14 | -9.83 | -39.5 | 450.6 | 29.46 | 0 | 3.6 | 12.5 | 40.7 | 330.8 | 47.71 |
| Bah15 | -14.18 | -41.67 | 473.37 | 44.96 | 0 | 4.9 | 12.3 | 68.15 | 481.8 | 70.77 |
| Bah16 | -10.72 | -43.65 | 506.06 | 30.72 | 0 | 0 | 5 | 45.95 | 187.6 | 46.05 |
| Bah17 | -13.16 | -41.77 | 1283.95 | 64.12 | 0 | 12 | 46.6 | 87.95 | 302 | 64.81 |
| Bah18 | -11.08 | -43.14 | 407.5 | 44.54 | 0 | 0 | 5.1 | 46.4 | 495 | 82.61 |
| Bah19 | -10.44 | -40.15 | 532.09 | 44.87 | 0 | 10.5 | 28 | 67.55 | 239.4 | 48.33 |
| Bah20 | -12.56 | -41.39 | 438.09 | 79.01 | 0 | 21.6 | 53.4 | 101.75 | 591.6 | 84.79 |
| Bah21 | -11.33 | -41.86 | 768.42 | 35.18 | 0 | 0.75 | 9.5 | 51.7 | 279 | 56.74 |
| Bah22 | -14.89 | -40.8 | 879.38 | 47.17 | 0 | 11.55 | 28 | 57.25 | 344.8 | 56.25 |
| Bah23 | -12.2 | -38.97 | 229.64 | 49.3 | 0 | 14.75 | 37.6 | 71.3 | 185.6 | 43.12 |
| Bah24 | -13.53 | -40.12 | 757.42 | 61.86 | 0 | 30.9 | 55 | 79.85 | 197.2 | 43.48 |
| Bah25 | -12.68 | -39.09 | 219.76 | 72.11 | 0 | 32.3 | 61 | 108.65 | 199.4 | 53.16 |
| Bah26 | -12.12 | -45.03 | 474.17 | 63.94 | 0 | 0 | 11.9 | 105.7 | 590.2 | 96.13 |
| Bah27 | -13.01 | -39.62 | 397.8 | 70.58 | 0 | 33.15 | 55.7 | 100.1 | 243.4 | 51.32 |
| Cea28 | -4.23 | -38.96 | 865.53 | 94.9 | 0 | 17.45 | 60.7 | 146.7 | 360.2 | 93.47 |
| Cea29 | -5.19 | -40.67 | 298.19 | 48.72 | 0 | 0.2 | 11.8 | 71.2 | 430.2 | 72.61 |
| Dis30 | -15.6 | -47.63 | 1030.36 | 111.25 | 0 | 5.4 | 80.8 | 191.85 | 461.2 | 112.65 |
| Dis31 | -15.79 | -47.93 | 1160.96 | 116.57 | 0 | 7.9 | 86 | 196 | 440.4 | 113.99 |
| Esp32 | -18.68 | -39.86 | 28.66 | 92.66 | 1.6 | 33.95 | 68.9 | 128.75 | 366.2 | 75.67 |
| Esp33 | -18.7 | -40.39 | 156.02 | 63.75 | 0 | 12.05 | 44.7 | 88.7 | 378.2 | 69.77 |
| Esp34 | -21.1 | -41.04 | 69.12 | 72.43 | 0 | 15.05 | 57.2 | 105.45 | 393.4 | 76.21 |
| Esp35 | -20.75 | -41.49 | 138 | 95.44 | 0 | 24.6 | 53.4 | 144.95 | 482.6 | 104.41 |
| Esp36 | -19.36 | -40.07 | 40 | 80.59 | 0 | 27.8 | 54.3 | 92.7 | 648.2 | 90.12 |
| Esp37 | -20.27 | -40.31 | 9 | 116.26 | 0 | 35.5 | 70.8 | 148.95 | 746.6 | 124.42 |
| Goi38 | -15.94 | -50.14 | 512.86 | 118.11 | 0 | 1.85 | 72.8 | 196.15 | 670.4 | 138.3 |
| Goi39 | -16.78 | -47.61 | 1211.08 | 126.47 | 0 | 4.9 | 85.2 | 193.65 | 590.4 | 141.88 |
| Goi40 | -17.75 | -49.1 | 751.09 | 95.87 | 0 | 4.05 | 71.5 | 157.7 | 354.8 | 98.53 |
| Goi41 | -17.92 | -51.72 | 670.08 | 117 | 0 | 14.95 | 89 | 209.3 | 353.8 | 104.52 |
| Goi42 | -16.26 | -47.97 | 1000.82 | 99.24 | 0 | 1.5 | 62.2 | 167.15 | 475.2 | 111.7 |
| Goi43 | -17.79 | -50.96 | 780.11 | 101.52 | 0 | 0.4 | 56.9 | 174.7 | 523.6 | 117.93 |
| Goi44 | -16.68 | -48.62 | 952 | 105.33 | 0 | 2 | 60.4 | 172.35 | 534.8 | 122.46 |
| Goi45 | -16.96 | -50.43 | 678.74 | 93.13 | 0 | 0.8 | 44.5 | 167.45 | 370 | 104.79 |
| Goi46 | -17.3 | -48.28 | 757.27 | 93.49 | 0 | 0 | 49.9 | 171.05 | 461.8 | 107.74 |
| Goi47 | -18.41 | -49.19 | 491.17 | 96.8 | 0 | 5.2 | 51.3 | 155.15 | 567.2 | 117.06 |
| Goi48 | -16.64 | -49.22 | 727.3 | 113.23 | 0 | 7.75 | 82.6 | 184.55 | 458.4 | 114.14 |
| Goi49 | -17.45 | -52.6 | 862 | 98.4 | 0 | 4.85 | 62.7 | 159.15 | 414 | 106.9 |
| Mar50 | -9.11 | -45.93 | 283.69 | 83.67 | 0 | 0 | 40.5 | 155.05 | 458.8 | 97.6 |
| Mar51 | -5.51 | -45.24 | 154.19 | 61.73 | 0 | 0.55 | 29.2 | 104.6 | 260.6 | 75.58 |
| Mar52 | -1.66 | -45.37 | 35.86 | 141.39 | 0 | 6.05 | 84.3 | 221.75 | 671.4 | 162.4 |
| Mat53 | -22.3 | -53.82 | 377.36 | 87.38 | 0 | 5.15 | 76.4 | 146.05 | 331.4 | 79.85 |
| Mat54 | -20.45 | -54.72 | 528.47 | 114.8 | 0 | 40.45 | 96.6 | 183.85 | 372.4 | 86.78 |
| Mat55 | -21.77 | -54.53 | 324.31 | 107.37 | 0 | 43.95 | 90.1 | 171.45 | 297.2 | 80.18 |
| Mat56 | -22.86 | -54.61 | 375.18 | 114.39 | 0 | 19.7 | 120.8 | 167.45 | 403 | 98.69 |
| Mat57 | -23.97 | -55.02 | 398.4 | 114.1 | 0 | 20.35 | 99.4 | 164.6 | 456.6 | 105.27 |
| Mat58 | -23.45 | -54.18 | 337.82 | 118.44 | 0 | 46.7 | 110.8 | 166.95 | 422 | 90.16 |
| Mat59 | -19.12 | -51.72 | 494.72 | 101.82 | 0 | 9.1 | 62.4 | 155.8 | 513.4 | 107.87 |
| Min60 | -22.31 | -45.37 | 1281.43 | 127.9 | 0.4 | 44.9 | 96.9 | 191.15 | 532.6 | 107.59 |
| Min61 | -22.4 | -44.96 | 1017.1 | 86.3 | 0 | 21.3 | 61.3 | 125.65 | 464.8 | 84.63 |
| Min62 | -18.2 | -45.46 | 931.01 | 97.92 | 0 | 2.4 | 50.4 | 152 | 494.8 | 117.17 |
| Min63 | -14.41 | -44.4 | 519.52 | 63.27 | 0 | 0 | 7.6 | 95.8 | 590.8 | 98 |
| Min64 | -20.45 | -45.45 | 878.14 | 100.39 | 0 | 19.1 | 55.3 | 143.15 | 515.6 | 111.28 |
| Min65 | -19 | -46.99 | 978.11 | 108.35 | 0 | 4.7 | 79.4 | 184.05 | 529 | 119.21 |
| Min66 | -17.8 | -40.25 | 211.97 | 69.88 | 0 | 19.1 | 45.4 | 89.7 | 341.6 | 72.82 |
| Min67 | -19.99 | -48.15 | 572.54 | 93.56 | 0 | 4.95 | 62.2 | 178.65 | 465.4 | 102.3 |
| Min68 | -19.54 | -49.52 | 559.07 | 112.23 | 0 | 12.4 | 85.6 | 165.05 | 536.2 | 117.49 |
| Min69 | -17.89 | -41.52 | 467.17 | 73.74 | 0 | 16.05 | 41.6 | 109.05 | 513.6 | 89.73 |
| Min70 | -21.77 | -43.36 | 936.88 | 121.59 | 0.2 | 28.6 | 72.9 | 180.25 | 512.4 | 121.1 |
| Min71 | -17.26 | -44.84 | 505.32 | 78.49 | 0 | 0.15 | 32.3 | 128.8 | 511.8 | 107.4 |
| Min72 | -16.55 | -46.88 | 640.85 | 96.22 | 0 | 1.2 | 39.3 | 158.4 | 513.4 | 117.89 |
| Min73 | -18.78 | -40.99 | 254.91 | 83.4 | 0 | 18.9 | 50.3 | 114.65 | 618.4 | 97.38 |
| Min74 | -15.09 | -44.02 | 454 | 53.03 | 0 | 0 | 11.5 | 81.4 | 423.6 | 82.68 |
| Min75 | -18.75 | -44.45 | 669.48 | 87.78 | 0 | 1.75 | 50.5 | 133.25 | 525.8 | 103.21 |
| Min76 | -15.3 | -45.62 | 873.2 | 86.46 | 0 | 0 | 34.5 | 152.3 | 439.4 | 106.56 |
| Min77 | -15.75 | -41.46 | 754.07 | 52.98 | 0 | 3.75 | 21.5 | 70.15 | 430.4 | 73.45 |
| Min78 | -16.58 | -41.49 | 271.63 | 45.04 | 0 | 3 | 12.6 | 53.95 | 351 | 69.21 |
| Min79 | -16.36 | -45.12 | 490.29 | 67.43 | 0 | 0 | 22.6 | 102.3 | 454.6 | 90.38 |
| Min80 | -15.72 | -42.44 | 850.06 | 57.53 | 0 | 4.15 | 25.3 | 79.2 | 350.8 | 76.72 |
| Min81 | -19.89 | -44.42 | 753.5 | 125.5 | 0 | 16.2 | 72.4 | 190.3 | 790.8 | 142.65 |
| Min82 | -18.79 | -42.94 | 852.68 | 84.1 | 0 | 9.5 | 49.3 | 122.35 | 573 | 100.89 |
| Min83 | -18.83 | -41.98 | 198.24 | 73.28 | 0 | 6 | 28.7 | 99.4 | 485.6 | 98.98 |
| Min84 | -16.16 | -42.31 | 487.14 | 56.86 | 0 | 3.15 | 15 | 74.8 | 354.2 | 81.6 |
| Min85 | -17.78 | -46.12 | 877.05 | 86.29 | 0 | 0.4 | 49.5 | 120.55 | 526 | 109.05 |
| Min86 | -19.74 | -42.14 | 609.25 | 83.72 | 0 | 4.8 | 40.7 | 133.5 | 520.4 | 104.09 |
| Min87 | -20.03 | -44.01 | 1198.82 | 127.46 | 0 | 19.35 | 79.4 | 200.35 | 803 | 140.75 |
| Min88 | -18.23 | -43.65 | 1359.25 | 109.42 | 0 | 12.2 | 59.8 | 178.25 | 635.8 | 128.3 |
| Min89 | -21.1 | -42.38 | 282.79 | 115.92 | 0 | 20.5 | 78.8 | 177.7 | 652 | 125.34 |
| Min90 | -21.92 | -46.38 | 1077.34 | 110.87 | 0 | 24.95 | 79.6 | 174.5 | 457.6 | 103.76 |
| Min91 | -21.57 | -45.4 | 949.78 | 92.2 | 0 | 18.65 | 65.4 | 133.8 | 471.8 | 97.91 |
| Min92 | -21.23 | -43.77 | 1168.76 | 117.81 | 0 | 23.7 | 80.1 | 179.7 | 723.6 | 121.24 |
| Min93 | -19.61 | -46.95 | 1018.32 | 120.29 | 0 | 14.3 | 89.4 | 185.9 | 532.2 | 122.02 |
| Min94 | -16.69 | -43.84 | 645.87 | 61.64 | 0 | 0.8 | 25.1 | 85.4 | 469.8 | 88.72 |
| Min95 | -18.92 | -48.26 | 874.77 | 124.28 | 0 | 16.7 | 89.5 | 208.1 | 596 | 122.52 |
| Min96 | -16.17 | -40.69 | 189.11 | 55.67 | 0 | 6.25 | 33.4 | 74.2 | 317.4 | 69.89 |
| Min97 | -20.75 | -46.63 | 781.7 | 85.51 | 0 | 5.25 | 53.4 | 149.45 | 367.2 | 92.74 |
| Min98 | -20.76 | -42.86 | 697.64 | 105.99 | 0 | 19.5 | 66.9 | 174.05 | 496 | 107.92 |
| Min99 | -19.57 | -42.62 | 493.42 | 106.6 | 0 | 13.05 | 61.4 | 145.1 | 721.2 | 128.54 |
| Min100 | -20.56 | -43.76 | 1048.2 | 106.07 | 0 | 13.9 | 58.3 | 180.6 | 509.6 | 116.71 |
| Min101 | -21.11 | -44.25 | 929.88 | 117.99 | 0 | 17 | 74.9 | 181.15 | 595.6 | 121.62 |
| Min102 | -22.86 | -46.04 | 1544.89 | 138.37 | 0 | 46.15 | 112.7 | 209.95 | 471.2 | 110.47 |
| Par103 | -24.78 | -50.05 | 1016 | 101.82 | 0 | 30.15 | 95.1 | 143.75 | 373.6 | 84.72 |
| Par104 | -23.41 | -51.93 | 548.51 | 130.22 | 0 | 57.8 | 127.7 | 183.05 | 373.2 | 91.29 |
| Par105 | -23.42 | -50.58 | 664.31 | 86.49 | 0 | 17.9 | 75.6 | 125.7 | 295.2 | 75.22 |
| Par106 | -25.7 | -53.1 | 545.95 | 123.88 | 0 | 35.85 | 117.5 | 186.3 | 509.2 | 102.36 |
| Par107 | -22.66 | -52.13 | 308.7 | 88.91 | 0 | 18.75 | 76.6 | 138.15 | 328.2 | 79.35 |
| Par108 | -23.36 | -52.93 | 365.79 | 100.08 | 0 | 27.1 | 83.9 | 153.95 | 433.8 | 89.31 |
| Par109 | -23.77 | -50.18 | 692.88 | 117.89 | 0 | 60.75 | 108.1 | 171.65 | 427.2 | 81.05 |
| Par110 | -25.51 | -48.81 | 50.31 | 141.58 | 0 | 63.75 | 120.4 | 186 | 540.6 | 110.56 |
| Par111 | -25.45 | -49.23 | 922.91 | 122.42 | 0 | 59.55 | 107.9 | 179.25 | 383.4 | 82.42 |
| Par112 | -7.23 | -35.9 | 546.17 | 51.52 | 0 | 8.1 | 29.5 | 86.35 | 342.8 | 59.57 |
| Par113 | -6.84 | -38.31 | 237.04 | 56.66 | 0 | 0.4 | 15.9 | 83.75 | 380 | 80.43 |
| Par114 | -6.98 | -35.72 | 573.45 | 89.21 | 0 | 24.75 | 54 | 150.15 | 459 | 88.61 |
| Par115 | -7.08 | -37.27 | 263.66 | 44.24 | 0 | 0.15 | 9.3 | 61.65 | 428.4 | 72.51 |
| Per116 | -8.06 | -34.96 | 11.3 | 163.03 | 1.8 | 59.2 | 119.2 | 246 | 677.6 | 139.09 |
| Per117 | -8.91 | -36.49 | 827.78 | 56.62 | 0 | 11.65 | 41.2 | 81.65 | 282.2 | 57.16 |
| Per118 | -8.5 | -39.32 | 342.74 | 28.1 | 0 | 1.75 | 8.9 | 38.95 | 204.2 | 43.59 |
| Per119 | -8.51 | -37.71 | 434.23 | 29.06 | 0 | 1.55 | 12.5 | 39.25 | 207.6 | 40.39 |
| Per120 | -8.6 | -38.58 | 327.42 | 28.79 | 0 | 1.7 | 12.8 | 37.25 | 185 | 40.46 |
| Per121 | -7.95 | -38.29 | 499.02 | 45.17 | 0 | 1.8 | 14.8 | 61.05 | 437.6 | 66.04 |
| Pia122 | -7.44 | -44.34 | 398.83 | 51.58 | 0 | 0 | 14.1 | 82.1 | 372.4 | 77.31 |
| Pia123 | -6.42 | -41.75 | 372 | 55.65 | 0 | 0.8 | 14.4 | 86.95 | 464 | 81.37 |
| Pia124 | -8.12 | -42.98 | 312.07 | 54.7 | 0 | 0 | 15.8 | 83.25 | 565 | 82.85 |
| Rio125 | -22.76 | -43.68 | 35 | 91.97 | 0 | 39.1 | 78.4 | 117.25 | 363.2 | 70.57 |
| Rio126 | -31.4 | -52.7 | 446.81 | 115.64 | 0 | 57.65 | 108.9 | 161.9 | 344.8 | 79.9 |
| Rio127 | -30.55 | -53.47 | 420.82 | 138.65 | 0 | 70 | 120.5 | 209.4 | 455.8 | 86.67 |
| Rio128* | -23.05 | -43.6 | 12 | 88.73 | 4 | 42 | 74.4 | 123.15 | 472 | 72.48 |
| Rio129 | -22.59 | -43.28 | 22 | 152.59 | 0 | 64.45 | 113.8 | 212.25 | 650 | 132 |
| Rio130 | -27.85 | -53.79 | 489.67 | 131.98 | 0 | 62.55 | 117.9 | 180.4 | 384 | 95.38 |
| Rio131* | -22.98 | -42.02 | 5 | 68.41 | 0 | 26.6 | 48.8 | 103.35 | 352 | 60.74 |
| Rio132 | -30.05 | -51.17 | 41.18 | 128.6 | 6.8 | 79 | 116 | 165.5 | 314 | 68.86 |
| Rio133 | -32.08 | -52.17 | 4.92 | 95.96 | 0 | 41.15 | 82.5 | 136.85 | 281.4 | 65.64 |
| Rio134 | -29.72 | -53.72 | 103.1 | 145.55 | 0 | 79.25 | 131.5 | 190.35 | 459.4 | 90.81 |
| Rio135* | -29.35 | -49.73 | 8.44 | 124.69 | 0 | 61.3 | 104.2 | 176.55 | 419.6 | 91.4 |
| Rio136 | -29.84 | -57.08 | 74.29 | 111.23 | 0 | 52.45 | 86.8 | 143.8 | 685.4 | 97.74 |
| Rio137 | -21.59 | -41.96 | 46 | 88.42 | 0 | 23.8 | 55.1 | 124.4 | 545 | 97.66 |
| Rio138* | -22.99 | -43.19 | 25.59 | 85.11 | 5.6 | 35.75 | 64.2 | 112 | 396.2 | 70.36 |
| Rio139 | -22.86 | -43.41 | 30.43 | 90.84 | 0 | 33.75 | 65.6 | 130.2 | 314.4 | 77.7 |
| Rio140 | -23.22 | -44.73 | 3 | 131.53 | 0 | 51.6 | 96.2 | 192.2 | 500.8 | 107.37 |
| Rio141 | -22.45 | -42.99 | 981 | 235.06 | 20.4 | 119.2 | 194.9 | 328.05 | 763.6 | 154.96 |
| Rio142 | -22.45 | -44.44 | 438.83 | 106.64 | 0 | 19.25 | 74.7 | 169.35 | 561.2 | 105.35 |
| Rio143 | -22.38 | -41.81 | 28 | 96.77 | 0 | 34.75 | 77.2 | 129.5 | 364.6 | 83.33 |
| Rio144 | -21.71 | -41.34 | 17 | 76.96 | 0 | 23.4 | 58.1 | 113.9 | 412.8 | 72.97 |
| Rio145 | -22.33 | -42.68 | 1070 | 140.21 | 0 | 48.45 | 98.5 | 194 | 539.2 | 122.16 |
| Rio146 | -29.87 | -52.38 | 106.99 | 115.41 | 0 | 60.1 | 104.3 | 158.45 | 410 | 77.69 |
| Rio147 | -31.35 | -54.01 | 226.19 | 117.38 | 0 | 57.3 | 99.5 | 165.4 | 471.2 | 84.63 |
| Rio148 | -28.75 | -50.06 | 1228.59 | 125.17 | 0 | 66.75 | 115.5 | 170 | 366.2 | 77.47 |
| Rio149 | -28.51 | -50.88 | 969.89 | 146.59 | 0 | 80.2 | 147 | 196.05 | 422 | 86.07 |
| Rio150 | -29.37 | -50.83 | 830.93 | 172.59 | 0 | 113.15 | 170.8 | 219.6 | 468 | 86.1 |
| Rio151 | -31.25 | -50.91 | 3.82 | 89.78 | 0 | 35.5 | 84.2 | 131.55 | 313.8 | 69.57 |
| Rio152 | -29.71 | -55.53 | 120.88 | 123.51 | 0 | 63.55 | 110.4 | 169.9 | 507.6 | 87.13 |
| Rio153 | -28.6 | -53.67 | 426.69 | 147.91 | 0 | 76.55 | 129.7 | 197.95 | 511.2 | 95.82 |
| Rio154 | -28.42 | -54.96 | 245.5 | 141.14 | 0 | 57.55 | 118.3 | 198.1 | 622.8 | 112.21 |
| Rio155 | -28.22 | -51.51 | 833.83 | 139.43 | 0 | 84.05 | 129 | 185.6 | 383 | 81.64 |
| Rio156 | -29.16 | -51.53 | 623.27 | 137.63 | 0 | 81.4 | 132.5 | 182.05 | 367 | 77.23 |
| Rio157 | -28.23 | -52.4 | 680.67 | 148.27 | 0.6 | 81.95 | 136.6 | 201.8 | 362.4 | 84.68 |
| Rio158 | -30.81 | -51.83 | 92.3 | 121.82 | 0 | 69.7 | 109.7 | 165.85 | 447.6 | 76.07 |
| Rio159 | -28.86 | -52.54 | 660.44 | 134.73 | 0 | 67.65 | 117.8 | 206.85 | 364.8 | 92.32 |
| Rio160 | -32.53 | -53.38 | 31.48 | 105.05 | 0 | 58.35 | 93.4 | 141.05 | 417.6 | 73.41 |
| Rio161* | -30.01 | -50.14 | 4.56 | 115.79 | 0 | 54 | 101.4 | 170.25 | 354.8 | 80.49 |
| Rio162 | -29.19 | -54.89 | 390.03 | 149.3 | 0 | 81.35 | 128.9 | 201.05 | 482 | 98.42 |
| Rio163 | -27.4 | -53.43 | 489.42 | 163.34 | 0 | 85 | 145.9 | 216.3 | 560.4 | 105.06 |
| Rio164 | -30.37 | -56.44 | 113.05 | 114.74 | 0 | 42.55 | 90.2 | 150.5 | 572 | 104.48 |
| Rio165 | -30.34 | -54.31 | 114.89 | 123.7 | 0 | 73.4 | 100.3 | 159.2 | 448.4 | 84.78 |
| Ron166 | -11.45 | -61.43 | 183.53 | 165.35 | 0 | 18.15 | 142.3 | 280.95 | 514.2 | 145.43 |
| San167 | -28.53 | -49.32 | 40.56 | 126.95 | 0 | 58.55 | 106.7 | 187.7 | 422.2 | 91.07 |
| San168 | -26.95 | -48.76 | 9.76 | 142.31 | 0 | 82.8 | 129.5 | 186.05 | 349.6 | 78.62 |
| San169 | -28.93 | -49.5 | 2 | 111.44 | 0 | 51.15 | 105.1 | 156.25 | 411.4 | 79.34 |
| San170 | -27.6 | -48.62 | 4.87 | 139.87 | 0 | 81.75 | 118.4 | 187.5 | 377.6 | 81.33 |
| San171 | -27.42 | -49.65 | 479.79 | 124.43 | 1 | 69.15 | 114.6 | 159.85 | 340 | 73.26 |
| San172 | -26.25 | -49.57 | 800 | 113.36 | 0 | 58.4 | 98.4 | 152.95 | 337.2 | 75.52 |
| San173 | -27.29 | -50.6 | 978.1 | 132.97 | 0 | 75.25 | 121.8 | 191.1 | 384.8 | 78.42 |
| San174 | -26.94 | -52.4 | 878.74 | 163.22 | 0 | 93.2 | 144.2 | 219.65 | 434.6 | 100.35 |
| San175 | -27.17 | -51.56 | 767.63 | 152.39 | 0 | 77.55 | 138.3 | 213.3 | 461.4 | 92.35 |
| San176 | -26.91 | -49.27 | 72.24 | 124.7 | 0 | 70.1 | 110.9 | 173.7 | 378 | 77.33 |
| San177* | -28.6 | -48.81 | 34.36 | 101.34 | 0 | 51.05 | 89.3 | 142.75 | 482.6 | 75.84 |
| San178 | -28.28 | -49.93 | 1400.06 | 141.75 | 0 | 84.6 | 137.8 | 182.4 | 409.2 | 78.46 |
| Sao179 | -23.43 | -47.59 | 609.33 | 91.48 | 0 | 20.1 | 75.3 | 149.5 | 396.6 | 79.65 |
| Sao180 | -23.89 | -48 | 710 | 90.94 | 0 | 28.25 | 76 | 137.4 | 310.6 | 75.54 |
| Sao181 | -23.04 | -45.52 | 582.26 | 93.6 | 0 | 24.45 | 63 | 145.65 | 361 | 86.4 |
| Sao182 | -20.16 | -50.59 | 460.44 | 97.81 | 0 | 13.75 | 73.5 | 148.7 | 451.4 | 101.61 |
| Sao183 | -23.23 | -45.42 | 862.3 | 86.51 | 0 | 17.85 | 55.1 | 144.2 | 429.2 | 86.84 |
| Sao184 | -21.09 | -49.92 | 408.35 | 86.37 | 0 | 8.15 | 60.5 | 134.95 | 414.2 | 86.87 |
| Sao185 | -21.34 | -48.11 | 540.41 | 77.96 | 0 | 9.75 | 53 | 129.1 | 368.8 | 81.97 |
| Sao186 | -20.56 | -48.54 | 534.36 | 93.25 | 0 | 14.5 | 68.4 | 145.05 | 337.2 | 92.59 |
| Sao187* | -24.67 | -47.55 | 2.7 | 164.79 | 0 | 70.95 | 144 | 234.55 | 671 | 126.76 |
| Sao188 | -21.32 | -50.93 | 381.9 | 113.77 | 0 | 32.35 | 86.6 | 168.55 | 413.6 | 100.49 |
| Sao189 | -21.98 | -47.88 | 859.29 | 115.63 | 0 | 23.2 | 84.2 | 189.6 | 458 | 105.43 |
| Sao190 | -22.12 | -51.41 | 431.92 | 108.94 | 0 | 42.1 | 99.8 | 155.1 | 347.2 | 83.91 |
| Sao191 | -22.75 | -45.6 | 1662.95 | 121.05 | 0 | 41 | 92.2 | 183 | 406.2 | 97.14 |
| Sao192 | -22.36 | -49.03 | 636.17 | 97.96 | 0 | 24.15 | 81.5 | 145.9 | 440.2 | 87.95 |
| Sao193 | -23.5 | -46.62 | 785.64 | 130.99 | 0.6 | 47.9 | 115.3 | 189.6 | 493.8 | 107.78 |
| Sao194 | -20.36 | -47.78 | 610.58 | 101.93 | 0 | 8.8 | 74 | 167.15 | 541.6 | 109.98 |
| Sao195 | -20.58 | -47.38 | 1002.74 | 113.25 | 0 | 13.45 | 81.5 | 179.7 | 417 | 110.29 |
| Ser196 | -11.27 | -37.79 | 204.8 | 77.43 | 0 | 25.2 | 60.6 | 114.85 | 318.2 | 69.63 |
| Toc197 | -8.97 | -48.18 | 189.71 | 135.46 | 0 | 2.9 | 117.5 | 228 | 588.2 | 132.14 |
| Toc198 | -10.15 | -48.31 | 267 | 108.05 | 0 | 0.15 | 74.2 | 185.75 | 499.8 | 118.78 |
| Toc199 | -11.74 | -49.05 | 274 | 113.03 | 0 | 0.15 | 67.6 | 201.8 | 455.2 | 122.07 |
| **Stations’ Average per VARAD1** | | | | | | | | | | |
| Ama-  zonas |  |  |  | 194.41 | 23.2 | 115.94 | 175.36 | 269.74 | 409.56 | 96.07 |
| Bahia |  |  |  | 66.33 | 10.47 | 34.72 | 59 | 80.4 | 290.99 | 44.94 |
| Ceará |  |  |  | 81.26 | 0 | 17.27 | 61.85 | 118.32 | 430.2 | 79.91 |
| Distrito Federal |  |  |  | 115.86 | 0 | 7.08 | 87.9 | 193.68 | 403.1 | 112.73 |
| Espírito Santo |  |  |  | 96.31 | 13.28 | 42.12 | 70.78 | 123.26 | 514.7 | 80.42 |
| Goiás |  |  |  | 114.56 | 0 | 12.54 | 98.97 | 193.04 | 394.79 | 105.84 |
| Maran-hão |  |  |  | 112.9 | 0 | 33.22 | 76.82 | 168.93 | 474.1 | 102.57 |
| Mato Grosso do Sul |  |  |  | 123.9 | 0.87 | 63.96 | 131.43 | 174.37 | 314.17 | 73.75 |
| Minas Gerais |  |  |  | 98.61 | 3.95 | 19.5 | 66.53 | 162.19 | 385.11 | 91.3 |
| Paraná |  |  |  | 128.38 | 5.3 | 81.72 | 119.29 | 174.18 | 326.02 | 68.74 |
| Paraíba |  |  |  | 68.23 | 0.5 | 19.31 | 53.55 | 97.98 | 314.27 | 61.35 |
| Pernam-buco |  |  |  | 64.34 | 4.29 | 24.19 | 52.31 | 101.05 | 204.53 | 47.51 |
| Piauí |  |  |  | 64.06 | 0 | 2.4 | 38.77 | 102.68 | 370.8 | 77.31 |
| Rio Grande do Sul |  |  |  | 119.15 | 10.25 | 59.93 | 97.38 | 174.66 | 318.25 | 76.89 |
| Rio de Janeiro |  |  |  | 140.54 | 25.48 | 94.45 | 137.46 | 175.72 | 307.73 | 60.42 |
| Rondônia |  |  |  | 165.35 | 0 | 18.15 | 142.3 | 280.95 | 514.2 | 145.43 |
| Santa Catarina |  |  |  | 140.97 | 13.39 | 90.31 | 140.3 | 187.17 | 303.86 | 63.78 |
| Sergipe |  |  |  | 115.71 | 3.11 | 51.21 | 99.09 | 165.74 | 312.15 | 80.64 |
| São Paulo |  |  |  | 77.43 | 0 | 25.2 | 60.6 | 114.85 | 318.2 | 69.63 |
| Tocantins |  |  |  | 131.27 | 0 | 6.33 | 106.3 | 218.17 | 675.3 | 128.31 |

1. **Colombia (COL-VARAD1)**

| Station | Lat | Lon | Elev | Mean | Min | 25% | 50% | 75% | Max | Std |
| --- | --- | --- | --- | --- | --- | --- | --- | --- | --- | --- |
| **Individual Stations** | | | | | | | | | | |
| Ant1 | 7.94 | -76.7 | 15 | 139.51 | 0 | 18.32 | 146.3 | 233.42 | 481.7 | 115.89 |
| Ant2 | 7.67 | -76.69 | 44 | 124.93 | 0 | 0.2 | 80.05 | 229.3 | 467.5 | 131.52 |
| Ant3 | 6.13 | -75.41 | 2125 | 67.72 | 0 | 0 | 37.25 | 115.08 | 338.3 | 80.36 |
| Ant4 | 6.28 | -74.67 | 1274 | 72.88 | 0 | 0 | 9.05 | 131.82 | 337.6 | 99.28 |
| Ant5 | 6.57 | -74.79 | 980 | 52.44 | 0 | 0 | 0 | 78.82 | 410.1 | 92.98 |
| Bog6 | 4.64 | -74.09 | 2556 | 59.44 | 0 | 12 | 47.5 | 88.72 | 207.7 | 54.55 |
| Bog7 | 4.35 | -74.15 | 3624 | 51.86 | 0 | 21.75 | 51.3 | 71.72 | 168.1 | 38.32 |
| Bog8 | 4.6 | -74.07 | 2646 | 57.09 | 0 | 24.5 | 48.85 | 76.4 | 238.8 | 45.1 |
| Boy9 | 5.65 | -74.07 | 742 | 118.98 | 0 | 35.52 | 102.85 | 175.52 | 473.9 | 106.56 |
| Boy10 | 6.51 | -72.45 | 3510 | 151.06 | 0 | 88.25 | 141.25 | 193.65 | 405.8 | 81.63 |
| Cau11 | 2.55 | -76.06 | 1800 | 73.68 | 0 | 18.47 | 63.5 | 114.65 | 269.1 | 64.94 |
| Cun12 | 4.31 | -74.81 | 309 | 51.09 | 0 | 5.42 | 27.25 | 77.4 | 249.4 | 56.01 |
| Cun13 | 5.33 | -74.02 | 2807 | 55.78 | 0 | 0 | 18.85 | 99.22 | 289.8 | 73.56 |
| Cun14 | 5.02 | -74.47 | 878 | 66.1 | 0 | 9.15 | 38.45 | 113.72 | 419.3 | 73.17 |
| Cun15 | 5.47 | -74.66 | 186 | 25.07 | 0 | 0 | 1.65 | 19.12 | 272.8 | 53.59 |
| Cun16 | 5.49 | -74.66 | 170 | 91.69 | 0 | 4.97 | 54.4 | 136.2 | 510.1 | 109.92 |
| Cun17 | 5.2 | -74.73 | 222 | 102.6 | 0 | 31.22 | 94.7 | 158.52 | 446.5 | 84.28 |
| Cun18 | 5.19 | -73.78 | 2618 | 31.79 | 0 | 6.18 | 22.2 | 44.48 | 252.5 | 36.52 |
| Cun19 | 4.78 | -73.87 | 3100 | 68.68 | 0 | 16.22 | 50.7 | 98.98 | 298.4 | 67.61 |
| Cun20 | 3.99 | -74.4 | 2957 | 72.24 | 0 | 27.75 | 59.2 | 107.25 | 296.9 | 63.04 |
| Cun21 | 4.31 | -74.31 | 2256 | 45.97 | 0 | 12.05 | 37.05 | 70.88 | 182.8 | 41.77 |
| Cun22 | 4.69 | -74.21 | 2543 | 13.56 | 0 | 0 | 2.85 | 21.4 | 128.7 | 21.05 |
| Cun23 | 5.09 | -74.02 | 3257 | 68.63 | 0 | 33.52 | 64.9 | 105.15 | 200.6 | 46.12 |
| Cun24 | 5.24 | -73.53 | 2300 | 82.6 | 0 | 36.28 | 76.4 | 114.28 | 310.7 | 62.88 |
| Cun25 | 4.01 | -74.5 | 1860 | 45.91 | 0 | 0 | 41.45 | 66.7 | 236.2 | 46.49 |
| Hui26 | 2.02 | -76.11 | 1919 | 88.63 | 0 | 0.78 | 90.8 | 136.1 | 244.2 | 72.01 |
| Hui27 | 1.93 | -76.43 | 1900 | 91.6 | 0 | 0 | 89.7 | 145.48 | 378.6 | 87.91 |
| Hui28 | 2.76 | -75.07 | 2101 | 83.66 | 0 | 42.58 | 84.3 | 120.22 | 309.4 | 61.02 |
| Hui29 | 2.22 | -76.12 | 1550 | 69.64 | 0 | 6.75 | 67.95 | 109.68 | 515.3 | 68.99 |
| LaG30 | 11.59 | -72.33 | 45 | 10.34 | 0 | 0 | 0 | 2.17 | 279.4 | 34.7 |
| LaG31 | 12.08 | -71.21 | 144 | 10 | 0 | 0 | 0 | 2.02 | 284.6 | 35.96 |
| Mag32* | 11.22 | -74.19 | 7 | 43.76 | 0 | 0 | 14.3 | 68.43 | 421.7 | 64.98 |
| Nor33 | 8.2 | -73.32 | 1220 | 55.21 | 0 | 2.28 | 33.7 | 92.5 | 270 | 61.34 |
| Nor34 | 7.51 | -72.49 | 240 | 59.28 | 0 | 7.78 | 54.25 | 93.38 | 284.9 | 55.17 |
| Nor35 | 7.9 | -72.49 | 311 | 21.9 | 0 | 0.1 | 9.85 | 31 | 222.1 | 34.01 |
| Nor36 | 7.54 | -72.77 | 1320 | 103.21 | 0 | 11.48 | 61.45 | 154.25 | 580.1 | 121.05 |
| San37 | 6.53 | -74.09 | 92 | 98.9 | 0 | 6.32 | 71.65 | 163.9 | 519.2 | 103.88 |
| San38 | 7.1 | -73.11 | 970 | 39.35 | 0 | 0.6 | 18.1 | 53.95 | 210.61 | 50.21 |
| San39 | 6.47 | -72.97 | 1673 | 166.04 | 0 | 71.02 | 156.85 | 254 | 506.1 | 121.91 |
| San40 | 5.99 | -73 | 2503 | 48.76 | 0 | 15.9 | 33.1 | 60.65 | 251.2 | 47.58 |
| San41 | 6.95 | -72.7 | 3600 | 103.14 | 0 | 36.1 | 90.8 | 156.27 | 287.7 | 75.89 |
| Tol42 | 4.87 | -75.17 | 323 | 92.52 | 0 | 36.03 | 71.25 | 132.1 | 370.5 | 82.21 |
| Tol43 | 4.44 | -75.5 | 2530 | 71.02 | 0 | 31.68 | 68.3 | 102.6 | 218.2 | 52.5 |
| Tol44 | 4.42 | -75.25 | 1323 | 65.74 | 0 | 0.18 | 38.5 | 114.4 | 376.4 | 78.88 |
| Tol45 | 3.44 | -75.12 | 345 | 91.58 | 0 | 11.35 | 54.7 | 163.65 | 425.8 | 94.02 |
| Tol46 | 4.19 | -74.96 | 393 | 16.09 | 0 | 0 | 0 | 2.12 | 400 | 50.69 |
| Tol47 | 3.73 | -74.83 | 464 | 39.49 | 0 | 0 | 1.5 | 51.62 | 417.3 | 71.62 |
| Tol48 | 4.08 | -75.7 | 3394 | 71.82 | 0 | 27.68 | 78.45 | 104.15 | 300.9 | 57.56 |
| Tol49 | 4.47 | -75.24 | 1946 | 97.03 | 0 | 0 | 58.5 | 172 | 395.1 | 108.2 |
| **Stations’ Average per VARAD1** | | | | | | | | | | |
| Antioquia |  |  |  | 93.51 | 3.92 | 50.84 | 88.34 | 123.36 | 275.42 | 55.25 |
| Bogotá D.C. |  |  |  | 57.46 | 3.93 | 33.48 | 51.85 | 75.47 | 159.33 | 33.34 |
| Boyacá |  |  |  | 135.05 | 0 | 69.16 | 118.72 | 177.26 | 397.8 | 84.94 |
| Cauca |  |  |  | 73.68 | 0 | 18.47 | 63.5 | 114.65 | 269.1 | 64.94 |
| Cundina-marca |  |  |  | 60.75 | 8.12 | 38.33 | 52.74 | 78.65 | 201.14 | 34.13 |
| Huila |  |  |  | 83.45 | 0 | 53.73 | 84.54 | 110.71 | 228.45 | 49.71 |
| La Guajira |  |  |  | 11.74 | 0 | 0 | 0.32 | 4.96 | 239.9 | 33.98 |
| Magda-lena |  |  |  | 43.76 | 0 | 0 | 14.3 | 68.43 | 421.7 | 64.98 |
| Norte de Santan-der |  |  |  | 60.22 | 0 | 24.21 | 47.61 | 81.3 | 272.4 | 51.02 |
| Santan-der |  |  |  | 93.4 | 8.45 | 53.84 | 95.3 | 128.54 | 196.66 | 46.43 |
| Tolima |  |  |  | 73.65 | 5.96 | 40.73 | 67.76 | 98.78 | 231.51 | 39.26 |
